# Using the phospha-Michael reaction for making phosphonium phenolate zwitterions

**DOI:** 10.3762/bjoc.20.6

**Published:** 2024-01-10

**Authors:** Matthias R Steiner, Max Schmallegger, Larissa Donner, Johann A Hlina, Christoph Marschner, Judith Baumgartner, Christian Slugovc

**Affiliations:** 1 Institute for Chemistry and Technology of Materials, Graz University of Technology, Stremayrgasse 9, 8010 Graz, Austria; 2 Christian Doppler Laboratory for Organocatalysis in Polymerization, Stremayrgasse 9, 8010 Graz, Austria; 3 Institute of Physical and Theoretical Chemistry, Graz University of Technology, Stremayrgasse 9, 8010 Graz, Austria; 4 Institute of Chemistry, Inorganic Chemistry, University of Graz, Schubertstraße 1, 8010 Graz, Austria; 5 Institute of Inorganic Chemistry, Graz University of Technology, Stremayrgasse 9, 8010 Graz, Austria

**Keywords:** Lewis-base catalysis, Michael acceptor reactivity, phospha-Michael reaction, phosphonium phenolate zwitterion

## Abstract

The reactions of 2,4-di-*tert*-butyl-6-(diphenylphosphino)phenol and various Michael acceptors (acrylonitrile, acrylamide, methyl vinyl ketone, several acrylates, methyl vinyl sulfone) yield the respective phosphonium phenolate zwitterions at room temperature. Nine different zwitterions were synthesized and fully characterized. Zwitterions with the poor Michael acceptors methyl methacrylate and methyl crotonate formed, but could not be isolated in pure form. The solid-state structures of two phosphonium phenolate molecules were determined by single-crystal X-ray crystallography. The bonding situation in the solid state together with NMR data suggests an important contribution of an ylidic resonance structure in these molecules. The phosphonium phenolates are characterized by UV–vis absorptions peaking around 360 nm and exhibit a negative solvatochromism. An analysis of the kinetics of the zwitterion formation was performed for three Michael acceptors (acrylonitrile, methyl acrylate, and acrylamide) in two different solvents (chloroform and methanol). The results revealed the proton transfer step necessary to stabilize the initially formed carbanion as the rate-determining step. A preorganization of the carbonyl bearing Michael acceptors allowed for reasonable fast direct proton transfer from the phenol in aprotic solvents. In contrast, acrylonitrile, not capable of forming a similar preorganization, is hardly reactive in chloroform solution, while in methanol the corresponding phosphonium phenolate is formed.

## Introduction

Organocatalysis has emerged in recent years as a valuable and powerful tool for performing organic reactions [1] and polymerizations [2]. In this context phosphines have proven to be potent Lewis-base catalysts [3–4] for a variety of reactions [5], including but not limited to Rauhut–Currier [6], Morita–Baylis–Hillman [7], and Michael reactions [8–10]. In all the mentioned reactions, the first step of the catalytic cycle is the nucleophilic attack of the phosphine on the electrophile, in many cases an electron-deficient olefin. The zwitterion formed from this conjugate addition can subsequently act as a nucleophile or as a base [3–5]. The efficiency of this zwitterion formation is of great importance since it is the initiation step for the catalytic cycle in Michael reactions [8]. Generally, the conjugate addition is favored for strong nucleophiles, which is why electron-rich trialkylphosphines were among the first catalysts used in this type of reaction [11–12]. Recently, our working group has investigated electron-rich triarylphosphines [13–15] as viable alternatives to alkylphosphines, which often suffer from their pronounced susceptibility to oxidation. In this regard, we wanted to explore hydroxy-substituted arylphosphines as potential candidates as well. *Ortho*-hydroxy-substituted phosphines have been mainly used as chelating ligands for metal complexes until recently [16–18]. Further, *ortho*-hydroxy phosphines have been used for the synthesis of probes in metabolic labeling [19], as a photocatalyst in the defluoroalkylation of trifluoromethyl groups [20] and the cross-coupling of aryl halides [21]. Like phosphonium salts in general are used as catalysts [22–23], phosphonium salts based on *ortho*-hydroxy-substituted phosphines received particular attention because of their zwitterionic nature and have been used as catalysts in the synthesis of carbonates from CO_2_ [24–26] and the synthesis of oxazolidines from isocyanates and epoxides [27]. Furthermore, their application in primary hydroxy group selective acylation of diols [28] and their use as organophotoredox catalysts [29–30] is known. The latter mentioned catalysts are regarded as stable phosphonium enolate zwitterions. The first zwitterions of this type were published in 1955 [31], but the first crystal structure of a phosphonium enolate zwitterion was reported only in 2007 by Zhu et al., who synthesized the compound via a three-component coupling between an alkylphosphine, an aldehyde and an alkyne [32]. Another example resulting from phosphine addition to α,β-unsaturated aldehydes was published shortly afterwards [33]. Phosphonium carboxylate zwitterions have been obtained by the reaction of phosphines with acrylic acid [8] and *ortho*-carboxylated arylphosphines with several Michael acceptors [34].

In this work we present the formation of stable zwitterions from the reaction of 2,4-di-*tert*-butyl-6-(diphenylphosphino)phenol (**1**) and a variety of different Michael acceptors and disclose kinetic investigations on the zwitterion formation with carbonyl and non-carbonyl-based Michael acceptors.

## Results and Discussion

### Synthesis

**Scheme 1:**
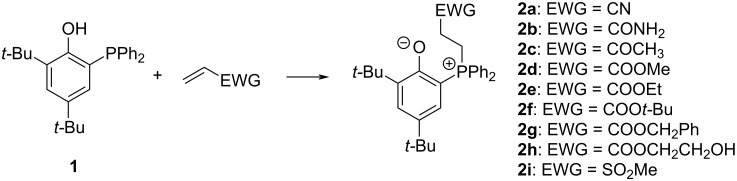
Reaction of **1** with various Michael acceptors (EWG = electron-withdrawing group) forming the zwitterions **2a–i**; the reactions were performed in dichloromethane at room temperature.

During our endeavors to identify potent Lewis-base catalysts for the oxa-Michael reaction [13–14], the triarylphosphine **1** was tested in a model reaction (2 equiv allyl alcohol, 1 equiv acrylonitrile, 0.05 equiv **1**). However, no conversion toward the desired product 3-(allyloxy)propanenitrile was observed after stirring the reaction mixture for 24 h at room temperature. Analyzing the reaction mixture with ^1^H NMR spectroscopy revealed the formation of a minor amount of a novel compound characterized by two multiplets centered at 3.31 and 3.09 ppm, respectively, and two novel signals for tertiary butyl groups. Accordingly, we reasoned that the phosphine has reacted presumably with acrylonitrile forming a stable species not suited to catalyze the oxa-Michael reaction. In order to identify this compound, we reacted **1** with acrylonitrile or with allyl alcohol (in both cases using a molar ratio of 1:1.05 and dichloromethane as the solvent). While in the latter case only the starting materials were observed after 24 h at room temperature, the reaction of **1** with acrylonitrile turned yellow during the same time and exclusively yielded the product of interest **2a**. Compound **2a** was identified by a combination of NMR spectroscopic methods and single-crystal X-ray structure analysis (vide infra) as the zwitterionic phospha-Michael adduct of **1** and acrylonitrile, formally stabilized by proton transfer from the phenol group to the initially formed carbanion [13–14]. Also with other Michael acceptors such as methyl vinyl ketone, several acrylates as well as methyl vinyl sulfone the reaction proceeds smoothly under the same reaction conditions (Scheme 1).

**Figure 1:**
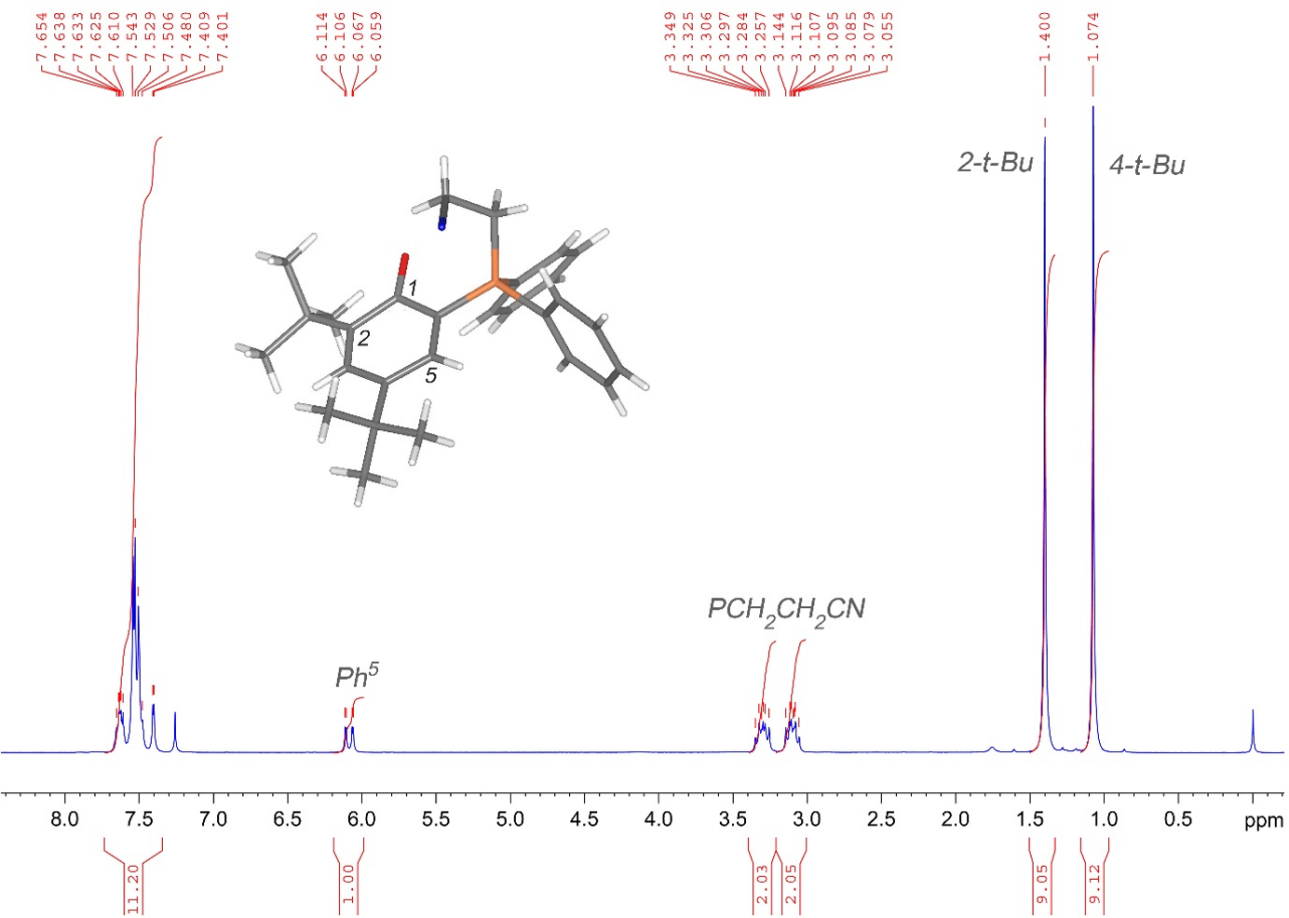
^1^H NMR spectrum of **2a** recorded on a 300 MHz spectrometer in CDCl_3_ at 23 °C; the inset shows a 3D-model based on the solid-state structure of **2a** and the numbering scheme of the phenolate moiety.

Conversions of **1** are usually quantitative within 24 h and all phosphonium phenolates can be purified by recrystallization whereby the solvents used vary depending on the parent Michael acceptor (for details, see Supporting Information File 1). Yields are not optimized and given in Table 1. The synthesized zwitterions were investigated via ^1^H, ^13^C and ^31^P NMR spectroscopy. All synthesized compounds exhibit similar features and characteristic resonances, like a set of two multiplets in the region between 3.00 and 2.70 as well as 3.50 and 3.10 ppm corresponding to the two methylene groups in between the phosphonium and the electron-withdrawing group (see Figure 1 for the case of **2a**). All compounds share a characteristic doublet of doublet pattern centered in the range of 6.21 to 6.09 ppm depending on the Michael acceptor used. This signal is attributed to the aromatic proton in position 5 of the 2,4-di-*tert*-butylphenol substituent that experiences a *meta*-coupling to the aromatic proton on position 3 (^4^*J*_HH_ ≈ 2.5 Hz) as well as coupling with the phosphonium center (^3^*J*_PH_ ≈ 14 Hz). In comparison to phosphine **1**, in which the same proton displays a resonance at 6.88 ppm (^4^*J*_HH_ = 2.5 Hz, ^3^*J*_PH_ = 5.8 Hz [35]), this signal is characteristically up-field shifted in every adduct **2a**–**j** (Table 1). The similar phosphonium salt 2,4-di-*tert*-butyl-6-(triphenylphosphonium)phenolate features this particular signal at 6.27 ppm (^4^*J*_HH_ = 2.7 Hz, ^3^*J*_PH_ = 14.4 Hz [30]). In the ^13^C NMR spectra, the chemical shifts of the carbon atoms in positions 1 and 6 of the phenolate unit are particularly noteworthy. In the adducts **2a**–**i**, the carbon atom 1, featuring the phenolate oxygen atom attached, shows a doublet (^2^*J*_PC_ ≈ 4 Hz) in the range of 175.0–173.9 ppm (Table 1). In the closely related 2,4-di-*tert*-butyl-6-(triphenylphosphonium)phenolate the chemical shift for the corresponding carbon atom appears at 173.8 ppm (^2^*J*_PC_ = 3.9 Hz) [30]. Compared to the parent phosphine **1** (155.9 ppm, ^2^*J*_PC_ = 19.3 Hz) [35] a pronounced down-field shift occurred upon adduct formation, which suggests a considerable contribution of a quinonic resonance structure as benzoquinones exhibit ^13^C NMR shifts of about 188 ppm and hydroquinones of about 150 ppm [36]. The opposite is true for the resonance of the carbon atom 6 having the phosphonium center attached, which is distinctly more shielded in **2a**–**i** (96.9–95.3 ppm, ^1^*J*_PC_ ≈ 100 Hz) then in **1** (119.9 ppm, ^1^*J*_PC_ = not observed) [35]. Likewise, the two *ipso*-carbons of the phenyl groups attached to the phosphonium center in **2a**–**i** are somewhat more shielded (≈124–125 ppm, ^1^*J*_PC_ ≈ 85–90 Hz) than in the starting material **1** (134.4 ppm, ^1^*J*_PC_ = 9 Hz). Comparison to alkyltriphenylphosphonium bromides reveals even more shielding of the *ipso*-carbons in these derivatives (117–116 ppm, ^1^*J*_PC_ ≈ 86 Hz) [37]. Concerning the ^13^C chemical shift for the aliphatic carbons directly attached to the phosphorus atom a slight down-field shift is found for **2a**–**i** (24–20 ppm, ^1^*J*_PC_ ≈ 64 Hz) when compared to similar alkyltriphenylphosphonium bromides (20–18 ppm, ^1^*J*_PC_ ≈ 54 Hz) [37]. Finally, the ^31^P NMR shift (against H_3_PO_4_, 85%) of the adducts is in the range of 20.9–18.9 ppm. Only the acrylamide-derived phosphonium phenolate **2b** is an exception, showing a ^31^P NMR shift of 25.1 ppm. The ^31^P NMR signal of 2,4-di-*tert*-butyl-6-(triphenylphosphonium)phenolate appears at 19.6 ppm [30]. Surprisingly, the ^31^P NMR shifts of the phosphonium phenolates are largely unaffected by changing a phenyl group for an alkyl group like in **2a**–**i**. Also other similarly substituted phosphonium salt species give the phosphorus signal in the range of 26–19 ppm [28,37–38]. For comparison, the phosphine **1** exhibits a ^31^P NMR shift of −29.7 ppm [35].

**Table 1:** Yields and characteristic ^1^H, ^13^C, and ^31^P NMR shifts of compounds **2a**–**i**.

Number	EWG	Yield [%]	^1^H NMR shift of Ph^5^ [ppm]	^13^C NMR shift of Ph^1^ [ppm]	^13^C NMR shift of Ph^6^ [ppm]	^31^P NMR shift [ppm]

**2a**	CN	85	6.09	175.0	95.5	18.9
**2b**	CONH_2_	42	6.14	174.1	96.6	25.1
**2c**	COCH_3_	61	6.13	174.8	96.9	20.7
**2d**	COOMe	46	6.20	174.9	96.1	19.4
**2e**	COOEt	49	6.19	174.9	96.2	19.4
**2f**	COO*t*-Bu	75	6.21	174.8	96.3	19.6
**2g**	COOCH_2_Ph	43	6.19	174.8	96.1	19.5
**2h**	COO(CH_2_)_2_OH	61	6.15	173.9	96.8	20.9
**2i**	SO_2_Me	18	6.14	175.0	95.3	19.7

In addition to the Michael acceptors presented in Scheme 1, the very weak Michael acceptors methyl methacrylate (electrophilicity parameter (E) for ethyl methacrylate is −22.77) and methyl crotonate (E for ethyl crotonate = −23.59) [39] were also tested as partner in the reaction with **1**. In these cases, the zwitterions formed to some extent (as evidenced by characteristic signals in the proton NMR spectra of the crude reaction mixtures) but could not be isolated in pure form. In case of methyl methacrylate, the reaction is accompanied by oligomerization of the Michael acceptor (Supporting Information File 1, Figure S47). A similar oligomerization reaction has been reported for cyanoacrylates [40]. Apart from the adducts of these two very weak Michael acceptors, the zwitterionic species **2a**–**i** described herein are quite stable. The stability of the zwitterions was evaluated exemplarily using the methyl acrylate adduct **2d**. Stability studies were conducted in the solid state and in solution. When storing the zwitterion under ambient conditions for a duration of two months, minor decomposition can be observed. The proton NMR gives rise to new signals appearing at 6.81 ppm (dd, *J* = 14.5, *J* = 2.2 Hz) and 11.23 ppm, which can be assigned to the phosphine oxide of **1** [41]. No free Michael acceptor (methyl acrylate) could be observed in the NMR spectrum (Supporting Information File 1, Figure S50). The ^31^P NMR spectrum (Supporting Information File 1, Figure S51) confirms the presence of the phosphine oxide of **1** as the main decomposition product, giving resonance at 41.4 ppm [42]. Besides, a number of new minor phosphorous species were detected. However, the total decomposition as indicated by ^31^P NMR can be estimated to be less than 5% (intensity of all phosphorous peaks from decomposition vs the intensity of the phosphorus signal for **2d**). Stability tests were also performed in two different deuterated solvents, CDCl_3_ and DMSO-*d*_6_. No air or moisture exclusion was applied. The tests were performed at room temperature and at 60 °C. ^1^H and ^31^P NMR spectra of the solutions were taken after 24, 48 and 72 h. At room temperature the observable decomposition after 72 h is very low in both solvents. NMR spectra (^1^H and ^31^P) show trace amounts of the phosphine oxide of **1**. The total amount of phosphine oxide is somewhat lower in DMSO-*d*_6_ when compared to CDCl_3_. Additionally, in CDCl_3_ further unknown decomposition products were detected in the ^31^P NMR, giving rise to peaks at 32.0 and 17.8 ppm. The overall decomposition at room temperature in both solvents after 72 h is less than 2%. At 60 °C, the decomposition is somewhat faster. After 72 h, the overall decomposition is less than 5% in both solvents according to ^31^P NMR spectra. Amongst the main decomposition product phosphine oxide, additional phosphorus signals point to the presence of free phosphine **1** and some unknown decomposition products (Supporting Information File 1, Figures S55 and S75). Moreover, small amounts of free methyl acrylate were detected in the ^1^H NMR spectra in these cases (Supporting Information File 1, Figures S54 and S74).

### Crystal structures

**Figure 2:**
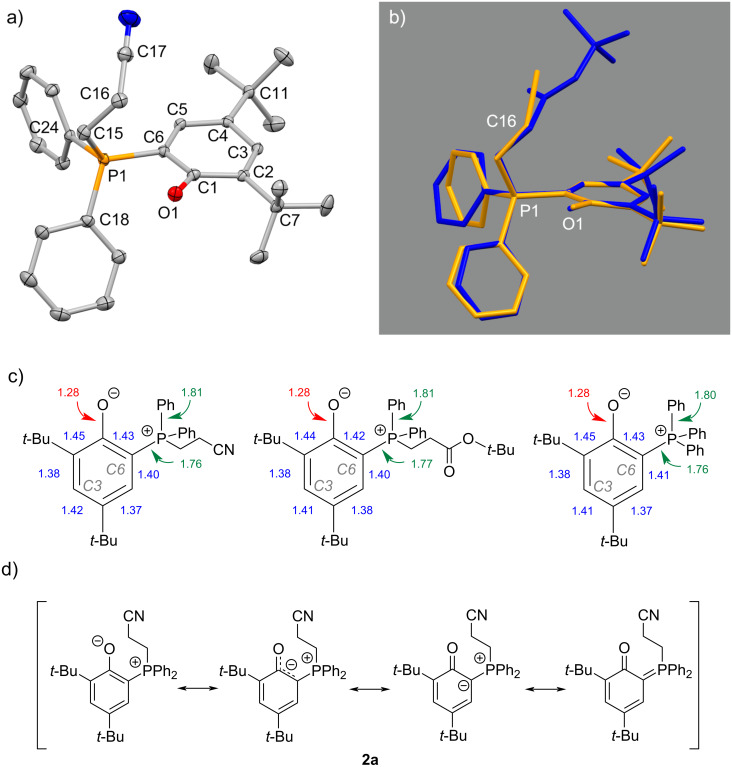
a) Molecular structure of **2a**, hydrogen atoms omitted for clarity, thermal ellipsoids drawn at 30% probability level. Selected distances (Å) and angles (deg): P1–C6 = 1.758(2), P1–C15 = 1.824(2), P1–C18 = 1.814(2), P1–C24 = 1.806(2), O1–C1 = 1.281(2), C15–C16 = 1.533(3), C16–C17 = 1.456(3), C17–N1 = 1.136(3), C6–C1–O1 = 118.6(2), C2–C1–O1 = 126.0(2), C6–P1–C24 = 107.64(8), C6–P1–C18 = 112.89(9), C15–P1–C24 = 108.88(9), C15–P1–C18 = 106.55(9), C15–P1–C6 = 115.33(9), C15–C16–C17 = 114.1(2); b) overlay of the molecular structures of **2a** (orange) and **2f** (blue); c) bond length of the phenolate substituent for **2a**, **2f** and 2,4-di-*tert*-butyl-6-(triphenylphosphonium)phenolate [30]; d) resonance structures for the description of the bonding situation in **2a**.

The solid-state structures of **2a** and **2f** were determined by single-crystal X-ray diffraction. The crystals were grown from concentrated solutions in toluene. A representation of the molecular structure of **2a** is shown in Figure 2a (for **2f** see Supporting Information File 1, Figure S1). Both molecules crystallize in a conformation in which the phenolate is oriented toward the methylene group in α-position (C16 in Figure 2b) to the electron-withdrawing group (either CN in case of **2a** or COO*t*-Bu in case of **2f**). The O1–C15 and O1–C16 distances are in **2a** 3.128(3) and 3.162.3(3) Å and in **2f** 3.098(4) and 3.019(4) Å, suggesting a weak hydrogen bonding interaction between O1 and the protons of the methylene groups [43]. The P1–O1 distances of 2.750(1) Å in **2a** and 2.693(3) Å in **2f** suggest an electrostatic interaction between the anionic phenolate and the cationic phosphonium center [32]. Other stable phosphonium enolate or phenolate zwitterions feature P–O distances in the range of 2.60–2.95 Å [28,32]. For comparison, in 1,2-oxaphosphetanes, the covalent bond between P–O is characterized by a distinctly shorter distance between the two atoms of 1.85 Å [44]. The bonding situation in the phenolate ring is of particular interest for understanding the zwitterions. The P1–C6 distances are with 1.758(2) Å in **2a** and 1.774(3) Å in **2f** significantly shorter than in the parent phosphine (1.825 Å) [35]. This points to a ylidic bonding situation in **2a** and **2f** similar to that observed in the related (triphenylphosphonium)phenolate [28,35] (Figure 2c). Also the O1–C1 distances (**2a**: 1.281(2) Å and **2f**: 1.279(3) Å) are very similar and between the values expected for a phenolate or a quinonic bonding [36]. In addition, the bond lengths pattern of the phenolate ring (see Figure 2c) and the torsion angle of P1–C6–C1–O1, which is 7.4(3)° in **2a** and 3.9(3)° in **2f**, suggests an electron delocalization within a ylidic system [27,30]. Accordingly, and based on the NMR spectroscopic investigation, the bonding situation in **2a**–**i** can be represented by the resonance structures shown in Figure 2d.

The phosphonium center exhibits a somewhat distorted tetrahedral conformation in both zwitterions. The largest angles found are 115.3(1)° in **2a** and 114.0(2)° in **2f** (in both cases C6–P1–C15) and the smallest angles are 105.0(1)° in **2a** and 104.3(2)° in **2f** (C18–P1–C24). A marginal shortening of about 0.02–0.03 Å of the bonds between P1 and the *ipso*-carbons of the aryl substituents in comparison to the parent phosphine **1** is observed. The alkyl groups attached to the phosphonium center do not show any special features. The distance between P1 and C15 is slightly longer (1.824(2) Å in **2a**; 1.828(3) in **2f**) when compared to the P–CH_2_ distance of a tetra-*n*-butylphosphonium cation [45].

### UV–vis spectroscopy

**Figure 3:**
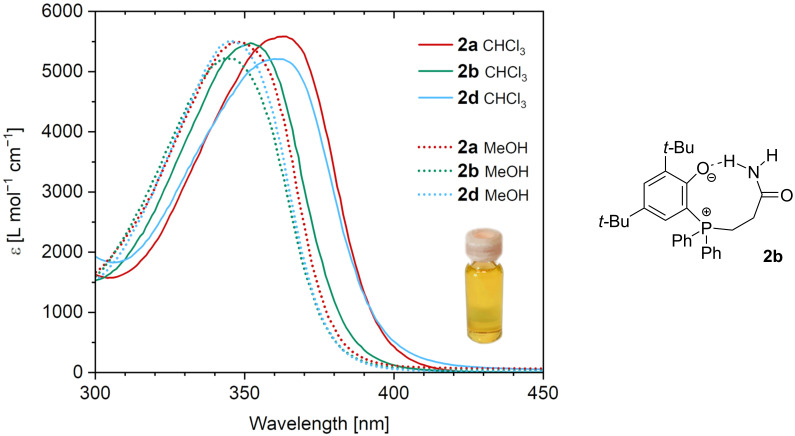
Left: UV–vis spectra of **2a**, **2b** and **2d** in chloroform (straight lines) and in methanol (dotted lines); the inset shows a photograph of a vial containing a solution of **2a** in chloroform; right: proposed hydrogen bonding interaction in **2b** in CHCl_3_.

All phosphonium phenolate compounds exhibit a bright yellow color in solution (see inset in Figure 3). Investigating the absorption properties in chloroform solution revealed an absorption feature ranging from about 310 to 420 nm peaking at 360 ± 3 nm (with molar absorption coefficients (ε) between 4000 and 6000 L mol^−1^ cm^−1^) for all zwitterions except **2b** and **2h** (Figure 3 and Supporting Information File 1, Figure S82). Compounds **2b**, the Michael adduct of acrylamide, and **2h**, the Michael adduct of 2-hydroxyethyl acrylate, show blue-shifted absorption maxima of 352 nm and 356 nm, respectively. Upon increasing the solvent polarity by using methanol instead of chloroform, a hypsochromic shift of the absorption maximum occurs (dotted lines in Figure 3). The blue shift is more pronounced for those zwitterions not bearing any hydrogen-bond-donating functional groups. Accordingly, it is plausible to explain the blue-shifted absorption maxima of **2b** and **2h** in chloroform by a more polar environment of the chromophore caused by the hydrogen-bond donors attached to the alkyl substituent of the phosphonium center (Figure 3). This hypothesis is further supported by the observation of two very different chemical shifts for the two amide protons in the ^1^H NMR spectrum of **2b** in CDCl_3_ giving resonance at 5.21 and 8.58 ppm.

### Kinetic studies

**Figure 4:**
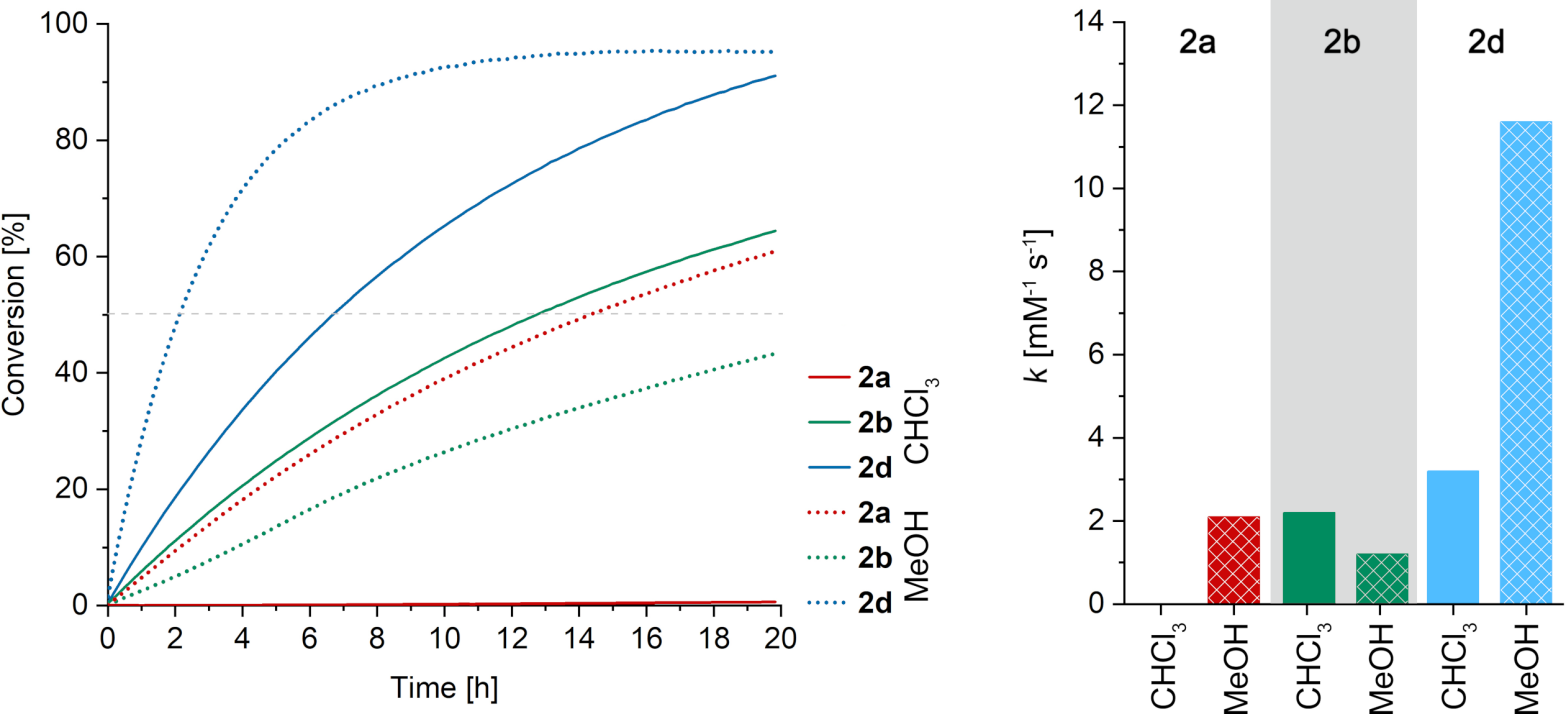
Conversion of **1** (initial *c* = 0.25 mM) toward **2a**, **2b**, or **2d** in the presence of the respective Michael acceptors (initial *c* = 7.5 mM) vs time as determined by the increase of the absorption band centered at 350–360 nm at 23 °C in either chloroform (solid lines) or methanol (dotted lines); right: 2nd order rate constants determined for the formation of **2a**, **2b**, and **2d** in chloroform (solid bars) and in methanol (checkered bars) at 23 °C.

In the next step the kinetics of the formation of the Michael adducts were studied. For this purpose, we used two strong and one weak Michael acceptors, which were selected according to their electrophilicity parameters (E) [39,46], their performance in previous testing [14] and the nature of the functional group. The strong Michael acceptors were methyl acrylate (E = −18.84) bearing a carbonyl-based electron-withdrawing group and acrylonitrile (E = −19.05) featuring a geometrically different electron-withdrawing group. Acrylamide was selected as a weak (E = −21.8), carbonyl-based Michael acceptor. The kinetic study was performed by monitoring the appearance of the zwitterion absorption by means of UV–vis spectroscopy in chloroform or in methanol as the solvent. The concentration of the respective Michael acceptor was varied ([Michael acceptor] = 2.5 mmol/L to 10 mmol/L) and was at least ten-fold higher than the concentration of the phosphine **1** ([**1**] = 0.25 mmol/L) to obtain pseudo first-order kinetics. Figure 4 shows typical time vs conversion plots for an initial Michael acceptor concentration of 7.5 mmol/L. Time conversion plots were then evaluated using COPASI [47]. To obtain second-order rate constants, we performed kinetic modelling (Supporting Information File 1, Figures S76–S81), fitting the experimental time traces by considering the second-order reaction shown in Scheme 1.

The strong Michael acceptor methyl acrylate quite readily yields the corresponding zwitterion **2d** in chloroform (*k*_CHCl3_ = 3.2 mM^−1^ s^−1^). Upon changing to methanol, the reaction becomes almost four times faster (*k*_MeOH_ = 11.6 mM^−1^ s^−1^). The reaction of **1** with the poor Michael acceptor acrylamide yielding **2b** in chloroform (*k*_CHCl3_ = 2.2 mM^−1^ s^−1^) is somewhat slower than the reaction with methyl acrylate. In this case, methanol has a detrimental effect on the reaction velocity as the rate constant (*k*_MeOH_ = 1.2 mM^−1^ s^−1^) is almost halved compared to chloroform. The strong Michael acceptor acrylonitrile reacts only very slowly in chloroform (*k*_CHCl3_ = 5.6 × 10^−3^ mM^−1^ s^−1^). In methanol, **2a** is formed with a similar rate constant (*k*_MeOH_ = 2.1 mM^−1^ s^−1^) as **2b**, the product of the poor Michael acceptor acrylamide in CHCl_3_. The results show no correlation of the rate constant with the electrophilicity parameter of the Michael acceptors.

**Scheme 2:**
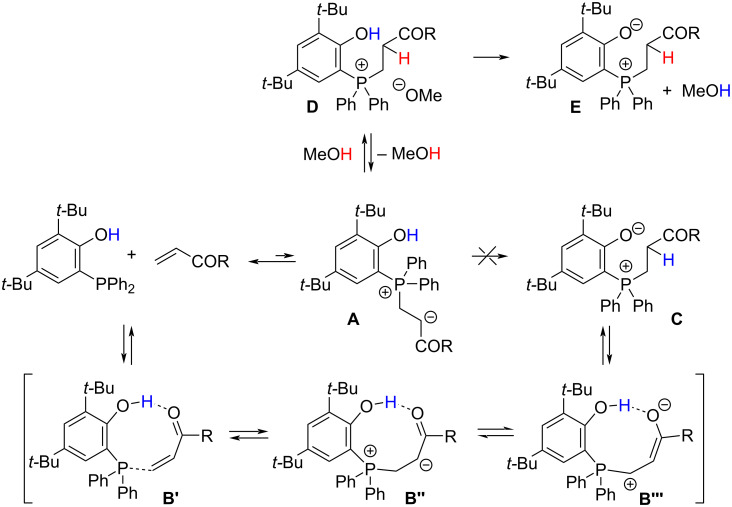
Proposed mechanism for intramolecular proton transfer in zwitterion formation with Michael acceptors bearing a carbonyl moiety and for the intermolecular proton transfer in the presence of the hydrogen-donor solvent methanol.

The low rate constant for the acrylonitrile reaction in chloroform suggests that the primary adduct **A** (see Scheme 2) is too short-lived that an intramolecular hydrogen transfer toward **2a** (**C**, in Scheme 2) is occurring [48]. Instead, in case of acrylonitrile another hydrogen bond donor, which is the solvent methanol [49], is necessary to trap intermediate **A** forming the ion pair **D**. Finally, deprotonation of the phenol moiety by the methoxide gives the final product **E** (**2a** when acrylonitrile is used as the Michael acceptor).

In this case, the proton at the α-position to the electron-withdrawing group is stemming from the protic solvent. Performing the reaction with methanol-*d*_4_ leads to incorporation of 0.7 equiv of deuterium in the α-position to the cyano group. However, this experiment does not allow for a conclusive distinction between the two postulated hydrogen transfer pathways as the phenolic hydrogen is quickly exchanged for deuterium under these conditions. The evenly strong Michael acceptor methyl acrylate reacts much faster in chloroform than acrylonitrile. A likely explanation is the preorganization of the Michael acceptor and donor by hydrogen bonding between the phosphine’s hydroxy group and the carbonyl group of the ester **B’**. Such a preorganization facilitates the proton transfer [34] from the hydroxy group to the initial zwitterion via **B’’** and **B’’’** resulting in **C**. In this case, the proton at the α-position to the electron-withdrawing group is stemming from the phenol moiety. Methyl acrylate in methanol is the fastest reaction presumably because both pathways, the intramolecular proton transfer and the methanol-mediated proton transfer, can occur. It has been described that intermediate **B** is more stable with enolizable electron-withdrawing groups such as esters [50] when compared to, e.g., a nitrile [49]. Accordingly, the intermolecular proton transfer pathway should be more accessible with methyl acrylate than with acrylonitrile. The lower reactivity of acrylamide in chloroform compared to methyl acrylate is in accordance with its lower electrophilicity. The observed rate reduction in methanol suggests the importance of the intramolecular hydrogen transfer pathway for the conversion of acrylamide, which is probably disturbed when the hydrogen bond donor solvent methanol is interacting with the amide group and/or the hydroxy group.

## Conclusion

The conjugate addition of 2,4-di-*tert*-butyl-6-(diphenylphosphino)phenol to Michael acceptor molecules allows for a facile modular synthesis of stable phosphonium phenolate zwitterions bearing additional functional groups. The bonding situation in the zwitterions was studied by NMR and UV–vis spectroscopies and single-crystal X-ray analysis of selected representatives. The zwitterions exhibit negative solvatochromism and feature considerable contribution of an ylidic resonance structure in the solid state and in aprotic solution. Kinetic studies revealed that the proton transfer from the phenolic hydroxy group to the initially formed zwitterionic adduct bearing the negative charge at the α-carbon to the electron-withdrawing group is the rate-determining step of the reaction. In an aprotic solvent, Michael acceptors bearing a carbonyl group allow for a preorganization of the reactants facilitating the proton transfer from the phenol and therefore a comparatively fast formation of the product. In protic solvents, the initial proton transfer stems predominantly from the solvent and Michael acceptors not suited for a preorganization react much faster compared to the aprotic solvent case.

## Experimental

All experiments were performed under ambient conditions. Chemicals were purchased from Sigma-Aldrich, Carl Roth, Merck, or TCI and were used as received. 2,4-Di-*tert*-butyl-6-(diphenylphosphino)phenol (**1**) was prepared according to a published procedure [51]. Stabilizers present in the Michael acceptors were not removed. NMR spectra were recorded on a Bruker AVANCE III 300 spectrometer or a JEOL JNM-ECZ 400 MHz spectrometer and are referenced to tetramethylsilane (^1^H, ^13^C), and 85% H_3_PO_4_ (^31^P). Deuterated solvents were obtained from Cambridge Isotope Laboratories Inc. UV–vis spectra were recorded on an Agilent Cary 60 UV–vis spectrophotometer. Kinetic evaluation was conducted assuming a second-order reaction as displayed in Scheme 1. All simulations were performed with COPASI, an open-source software [48]. The second-order rate constants were obtained by fitting the experimental time traces until a fully consistent data set, being valid for all experimental conditions, was established. For X-ray structure analyses the crystals were mounted onto the tips of glass fibres. Data collection was performed with a Bruker-AXS SMART APEX CCD diffractometer using graphite-monochromated Mo Kα radiation (0.71073 Å). The data were reduced to F_o_^2^ and corrected for absorption effects with SAINT (Version 6.45, Bruker AXS Inc., 1997–2003) and SADABS (Version 2.10. Bruker AXS Inc.), respectively [52]. The structures were solved by direct methods and refined by full-matrix least-squares method (SHELXL97 or SHELXL19) [53]. If not noted otherwise all non-hydrogen atoms were refined with anisotropic displacement parameters. All hydrogen atoms were located in calculated positions to correspond to standard bond lengths and angles. Figures of solid state molecular structures and the overlay of the molecular structures were generated using Mercury 2022.3.0 (Build 364735) [54]. Crystallographic data for the structures reported in this paper have been deposited with the Cambridge Crystallographic Data Centre as supplementary publication no. CCDC 2287962 (**2a**) and CCDC 2287963 (**2f**).

**Synthesis of zwitterions exemplarily given for 2b**. In a standard procedure **1** (0.2 mmol, 78 mg, 1 equiv) was dissolved in 0.5 mL dichloromethane in a 4 mL screw-cap vial. The Michael acceptor acrylamide (14.9 mg, 0.21 mmol, 1.05 equiv) was dissolved in 0.5 mL dichloromethane in a separate vial and then added dropwise to the solution of **1**. Zwitterion formation was indicated by a color change of the solution to yellow. The reaction mixture was stirred at room temperature for 24 h and the solvent evaporated. The product was recrystallized from a hot toluene/THF mixture. Yield: 38.8 mg (42%) off-white solid. ^1^H NMR (δ in ppm, 300 MHz, CDCl_3_, 298 K) 1.07 (s, 9H, C*H_3_*), 1.40 (s, 9H, C*H_3_*), 2.66–2.81 (m, 2H, C*H_2_*), 3.30–3.46 (m, 2H, C*H_2_*), 5.21 (br, 1H, NH_2_), 6.14 (dd, ^3^*J*_P-H_ = 14.4 Hz, ^4^*J*_H-H_ = 2.6 Hz, 1H, H5), 7.41–7.56 (m, 9H, Ar-*H*), 7.58–7.67 (m, 2H, Ar-*H*), 8.58 (br, 1H, NH_2_); ^13^C{^1^H} NMR (δ in ppm, 75 MHz, CDCl_3_, 298 K) 24.2 (d, ^1^*J*_P-C_ = 60.7 Hz, *C*H_2_), 29.4 (s, *C*H_3_), 30.9 (d, ^2^*J*_P-C_ = 4.0 Hz, *C*H_2_), 31.4 (s, *C*H_3_), 33.9 (d, ^4^*J*_P-C_ = 1.2 Hz, *C*CH_3_), 35.0 (d, ^4^*J*_P-C_ = 2.2 Hz, *C*CH_3_), 96.6 (d, ^1^*J*_P-C_ = 99.0 Hz, C6), 124.6 (d, ^1^*J*_P-C_ = 86.0 Hz, C_i-Ph_), 127.3 (d, ^2^*J*_P-C_ = 12.5 Hz, C5), 129.4 (d, ^3^*J*_P-C_ = 11.9 Hz, C4), 131.3 (d, ^4^*J*_P-C_ = 1.4 Hz, C3), 132.6 (d, ^3^*J*_P-C_ = 9.3 Hz, C2), 132.9 (d, ^3^*J*_P-C_ = 2.7 Hz, C_m-Ph_), 133.4 (d, ^2^*J*_P-C_ = 14.8 Hz, C_o-Ph_), 140.5 (d, ^4^*J*_P-C_ = 8.0 Hz, C_p-Ph_), 174.1 (d, ^2^*J*_P-C_ = 4.4 Hz, C1), 174.8 (d, ^3^*J*_P-C_ = 13.9 Hz, CO); ^31^P{^1^H} NMR (δ in ppm, 162 MHz, CDCl_3_, 298 K) 25.1; UV–vis (CHCl_3_): λ_max_ = 352 nm (ε = 5.53 × 10^3^ L mol^−1^ cm^−1^).

## Supporting Information

Accession codes CCDC 2287962 and 2287963 contain the supplementary crystallographic data for **2a** and **2f**, respectively. These data can be obtained free of charge via https://www.ccdc.cam.ac.uk/data_request/cif, or by emailing data_request@ccdc.cam.ac.uk, or by contacting Cambridge Crystallographic Data Centre, 12 Union Road, Cambridge CB2 1EZ, UK; fax: +44 1223 336033.

File 1 Experimental procedures, plot of the solid-state structure of **2f**, crystallographic data, NMR spectra, UV–vis spectra and experimental and simulated time conversion plots for the zwitterion formation.

## Data Availability

All data that supports the findings of this study is available in the published article and/or the supporting information to this article.

## References

[1] MacMillan D W C (2008). Nature.

[2] Bossion A, Heifferon K V, Meabe L, Zivic N, Taton D, Hedrick J L, Long T E, Sardon H (2019). Prog Polym Sci.

[3] Xie C, Smaligo A J, Song X-R, Kwon O (2021). ACS Cent Sci.

[4] Guo H, Fan Y C, Sun Z, Wu Y, Kwon O (2018). Chem Rev.

[5] Khong S, Venkatesh T, Kwon O (2021). Asian J Org Chem.

[6] Aroyan C E, Dermenci A, Miller S J (2009). Tetrahedron.

[7] Basavaiah D, Rao A J, Satyanarayana T (2003). Chem Rev.

[8] Salin A V, Shabanov A A (2023). Catal Rev: Sci Eng.

[9] Mather B D, Viswanathan K, Miller K M, Long T E (2006). Prog Polym Sci.

[10] Ratzenböck K, Fischer S M, Slugovc C (2023). Monatsh Chem.

[11] Horner L, Jurgeleit W, Klüpfel K (1955). Justus Liebigs Ann Chem.

[12] Morita K-i, Suzuki Z, Hirose H (1968). Bull Chem Soc Jpn.

[13] Fischer S M, Renner S, Boese A D, Slugovc C (2021). Beilstein J Org Chem.

[14] Fischer S M, Kaschnitz P, Slugovc C (2022). Catal Sci Technol.

[15] Fischer S M, Schallert V, Uher J M, Slugovc C (2023). Polym Chem.

[16] Rauchfuss T B (1977). Inorg Chem.

[17] Canestrari M, Chaudret B, Dahan F, Huang Y-S, Poilblanc R, Kim T-C, Sanchez M (1990). J Chem Soc, Dalton Trans.

[18] Hlina J A, Pankhurst J R, Kaltsoyannis N, Arnold P L (2016). J Am Chem Soc.

[19] Row R D, Shih H-W, Alexander A T, Mehl R A, Prescher J A (2017). J Am Chem Soc.

[20] Liu C, Shen N, Shang R (2022). Nat Commun.

[21] Shen N, Li R, Liu C, Shen X, Guan W, Shang R (2022). ACS Catal.

[22] Werner T (2009). Adv Synth Catal.

[23] Li H, Liu H, Guo H (2021). Adv Synth Catal.

[24] Büttner H, Steinbauer J, Wulf C, Dindaroglu M, Schmalz H-G, Werner T (2017). ChemSusChem.

[25] Toda Y, Hashimoto K, Mori Y, Suga H (2020). J Org Chem.

[26] Toda Y, Komiyama Y, Esaki H, Fukushima K, Suga H (2019). J Org Chem.

[27] Toda Y, Gomyou S, Tanaka S, Komiyama Y, Kikuchi A, Suga H (2017). Org Lett.

[28] Toda Y, Sakamoto T, Komiyama Y, Kikuchi A, Suga H (2017). ACS Catal.

[29] Toda Y, Tanaka K, Matsuda R, Sakamoto T, Katsumi S, Shimizu M, Ito F, Suga H (2021). Chem Commun.

[30] Toda Y, Kobayashi T, Hirai F, Yano T, Oikawa M, Sukegawa K, Shimizu M, Ito F, Suga H (2023). J Org Chem.

[31] Horner L, Klüpfel K (1955). Justus Liebigs Ann Chem.

[32] Zhu X-F, Henry C E, Kwon O (2007). J Am Chem Soc.

[33] Moiseev D V, Patrick B O, James B R, Hu T Q (2007). Inorg Chem.

[34] Salin A V, Fatkhutdinov A R, Il'in A V, Galkin V I (2014). Int J Chem Kinet.

[35] Heinicke J, Kadyrov R, Kindermann M K, Koesling M, Jones P G (1996). Chem Ber.

[36] Kim B, Storch G, Banerjee G, Mercado B Q, Castillo-Lora J, Brudvig G W, Mayer J M, Miller S J (2017). J Am Chem Soc.

[37] Haitham E, Yaccoubi F (2023). Phosphorus, Sulfur Silicon Relat Elem.

[38] Xu C, Li T, Jiang P, Zhang Y J (2020). Tetrahedron.

[39] Allgäuer D S, Jangra H, Asahara H, Li Z, Chen Q, Zipse H, Ofial A R, Mayr H (2017). J Am Chem Soc.

[40] Gololobov Y G, Gololobov M Y (2010). C R Chim.

[41] He L-P, Mu H-L, Li B-X, Li Y-S (2010). J Polym Sci, Part A: Polym Chem.

[42] Zhang M, Jia X, Zhu H, Fang X, Ji C, Zhao S, Han L-B, Shen R (2019). Org Biomol Chem.

[43] Desiraju G R (1991). Acc Chem Res.

[44] Hamaguchi M, Iyama Y, Mochizuki E, Oshima T (2005). Tetrahedron Lett.

[45] Kuotsu V, Nakro V, Yanger I, Lotha T N, Tzudir K, Sinha U B, Jamir L (2021). Green Chem Lett Rev.

[46] Mayer R J, Allihn P W A, Hampel N, Mayer P, Sieber S A, Ofial A R (2021). Chem Sci.

[47] Hoops S, Sahle S, Gauges R, Lee C, Pahle J, Simus N, Singhal M, Xu L, Mendes P, Kummer U (2006). Bioinformatics.

[48] Salin A V, Fatkhutdinov A R, Il'in A V, Sotov E I, Sobanov A A, Galkin V I, James B R (2013). J Phys Org Chem.

[49] Salin A V, Sobanov A A, Bakhtiyarova Y V, Khabibullin A A, Galkin V I, Cherkasov R A (2011). Phosphorus, Sulfur Silicon Relat Elem.

[50] Salin A V, Khisamova D R (2020). J Mol Liq.

[51] Thevenon A, Cyriac A, Myers D, White A J P, Durr C B, Williams C K (2018). J Am Chem Soc.

[52] Blessing R H (1995). Acta Crystallogr, Sect A: Found Crystallogr.

[53] Sheldrick G M (2008). Acta Crystallogr, Sect A: Found Crystallogr.

[54] Macrae C F, Sovago I, Cottrell S J, Galek P T A, McCabe P, Pidcock E, Platings M, Shields G P, Stevens J S, Towler M (2020). J Appl Crystallogr.

