# Supplementary material for: Using the phospha-Michael reaction for making phosphonium phenolate zwitterions
Source: Beilstein J Org Chem. 2024 Jan 10;20:41–51. doi: 10.3762/bjoc.20.6 (PMC10790659; doi:10.3762/bjoc.20.6)
Supplement: File 1 — Experimental procedures, plot of the solid-state structure of 2f, crystallographic data, NMR spectra, UV–vis spectra and experimental and simulated time conversion plots for the zwitterion formation. [file Beilstein_J_Org_Chem-20-41-s001.pdf]

BEILSTEIN JOURNAL OF ORGANIC CHEMISTRY

Supporting Information

for

Using the phospha-Michael reaction for making phosphonium phenolate zwitterions

Matthias R. Steiner, Max Schmallegger, Larissa Donner, Johann A. Hlina, Christoph Marschner, Judith Baumgartner and Christian Slugovc

Beilstein J. Org. Chem. **2024,** *20,* 41–51. doi:10.3762/bjoc.20.6

Experimental procedures, plot of the solid-state structure of 2f, crystallographic data, NMR spectra, UV–vis spectra and experimental and simulated time conversion plots for the zwitterion formation

License and Terms: This is a supporting information file under the terms of the Creative Commons Attribution License (https://creativecommons.org/licenses/by/4.0). Please note that the reuse, redistribution and reproduction in particular requires that the author(s) and source are credited and that individual graphics may be subject to special legal provisions.
The license is subject to the *Beilstein Journal of Organic Chemistry* terms and conditions: (https://www.beilstein-journals.org/bjoc/terms)

Synthesis of zwitterions. In a standard procedure **1** (0.2 mmol, 78 mg, 1 equiv) was dissolved in 0.5 mL dichloromethane in a 4 mL screw-cap vial. The Michael acceptor (0.21 mmol, 1.05 equiv) was dissolved in 0.5 mL dichloromethane in a separate vial and then added to the solution of **1** dropwise. Zwitterion formation was indicated by a color change to yellow of the solution. The reaction mixture was stirred at room temperature for 24 h and the solvent evaporated. The compounds were purified via recrystallization.

2a. This compound was synthesized via a different procedure. The appropriate amount of phosphine (0.20 mmol, 78 mg) was dissolved in 0.3 mL acrylonitrile and stirred over night at room temperature. The excess of acrylonitrile was evaporated and the remaining yellow solid washed three times with pentane (1 mL each) and then dried to obtain the pure product as a yellow powder. (Yield: 75 mg, 85%) ^{1}H NMR (δ in ppm, 300 MHz, $CDCl_3$, 298 K): 1.08 (s, 9H, CH_3), 1.40 (s, 9H, CH_3), 3.02-3.17 (m, 2H, CH_2), 3.22-3.38 (m, 2H, CH_2), 6.09 (dd, $^3J_{P\text{-}H}$ = 14.1 Hz, $^4J_{H\text{-}H}$ = 2.4 Hz, 1H, H5), 7.41 (d, $^4J_{H\text{-}H}$ = 2.4 Hz, 1H, H3), 7.46-7.59 (m, 8H, Ar-*H*), 7.60-7.69 (m, 2H, Ar-*H*). ^{13}C{^{1}H} NMR (δ in ppm, 75 MHz, $CDCl_3$, 298 K): 13.0 (s, *C*H$_2$-CN), 23.9 (d, $^1J_{P\text{-}C}$ = 64.3 Hz, *C*H$_2$), 28.9 (s, *C*H$_3$), 31.3 (s, *C*H$_3$), 33.9 (d, $^4J_{P\text{-}C}$ = 1.5 Hz, *C*CH$_3$), 34.9 (d, $^4J_{P\text{-}C}$ = 2.2 Hz, *C*CH$_3$), 95.5 (d, $^1J_{P\text{-}C}$ = 100.4 Hz, C6), 118.9 (d, $^3J_{P\text{-}C}$ = 18.3 Hz, *C*N), 124.4 (d, $^1J_{P\text{-}C}$ = 87.3 Hz, $C_{i\text{-}Ph}$), 126.4 (d, $^2J_{P\text{-}C}$ = 13.1 Hz, C5), 129.3 (d, $^3J_{P\text{-}C}$ = 12.0 Hz, C4), 131.3 (d, $^4J_{P\text{-}C}$ = 1.4 Hz, C3), 132.5 (s, $C_{o\text{-}Ph}$), 132.7 (d, $^3J_{P\text{-}C}$ = 9.3 Hz, C2), 132.9 (d, $^3J_{P\text{-}C}$ = 2.8 Hz, $C_{m\text{-}Ph}$), 140.1 (d, $^4J_{P\text{-}C}$ = 8.1 Hz, $C_{p\text{-}Ph}$), 175.0 (d, $^2J_{P\text{-}C}$ = 4.0 Hz, C1). ^{31}P{^{1}H} NMR (δ in ppm, 162 MHz, $CDCl_3$, 298 K): 18.9. UV-Vis ($CHCl_3$): λ_{max} = 363 nm (ε = 5.64 * 10^3 L mol^{-1} cm^{-1}).

2b. 0.21 mmol (14.9 mg) acrylamide was used. The product was recrystallized from a hot toluene/THF mixture (off-white solid, Yield: 38.8 mg, 42%). ^{1}H NMR (δ in ppm, 300 MHz, $CDCl_3$, 298 K): 1.07 (s, 9H, CH_3), 1.40 (s, 9H, CH_3), 2.66-2.81 (m, 2H, CH_2), 3.30-3.46 (m, 2H, CH_2), 5.21 (br, NH_2), 6.14 (dd, $^3J_{P\text{-}H}$ = 14.4 Hz, $^4J_{H\text{-}H}$ = 2.6 Hz, 1H, H5), 7.41-7.56 (m, 9H, Ar-*H*), 7.58-7.67 (m, 2H, Ar-*H*), 8.58 (br, NH_2). ^{13}C{^{1}H} NMR (δ in ppm, 75 MHz, $CDCl_3$, 298 K): 24.2 (d, $^1J_{P\text{-}C}$ = 60.7 Hz, *C*H$_2$), 29.4 (s, *C*H$_3$), 30.9 (d, $^2J_{P\text{-}C}$ = 4.0 Hz, *C*H$_2$), 31.4 (s, *C*H$_3$), 33.9 (d, $^4J_{P\text{-}C}$ = 1.2 Hz, *C*CH$_3$), 35.0 (d, $^4J_{P\text{-}C}$ = 2.2 Hz, *C*CH$_3$), 96.6 (d, $^1J_{P\text{-}C}$ = 99.0 Hz, C6), 124.6 (d, $^1J_{P\text{-}C}$ = 86.0 Hz, $C_{i\text{-}Ph}$), 127.3 (d, $^2J_{P\text{-}C}$ = 12.5 Hz, C5), 129.4 (d, $^3J_{P\text{-}C}$ = 11.9 Hz, C4), 131.3 (d, $^4J_{P\text{-}C}$ = 1.4 Hz, C3), 132.6 (d, $^3J_{P\text{-}C}$ = 9.3 Hz, C2), 132.9 (d, $^3J_{P\text{-}C}$ = 2.7 Hz, $C_{m\text{-}Ph}$), 133.4 (d, $^2J_{P\text{-}C}$ = 14.8 Hz, $C_{o\text{-}Ph}$), 140.5 (d, $^4J_{P\text{-}C}$ = 8.0 Hz, $C_{p\text{-}Ph}$), 174.1 (d, $^2J_{P\text{-}C}$ = 4.4 Hz, C1), 174.8 (d, $^3J_{P\text{-}C}$ = 13.9 Hz, CO). ^{31}P{^{1}H} NMR (δ in ppm, 162 MHz, $CDCl_3$, 298 K): 25.1. UV-Vis ($CHCl_3$): λ_{max} = 352 nm (ε = 5.53 * 10^3 L mol^{-1} cm^{-1}).

2c. 0.21 mmol (14.7 mg) methyl vinyl ketone was used. The product was recrystallized from hot hexane (yellow solid, Yield: 56.2 mg, 61%). ^{1}H NMR (δ in ppm, 300 MHz, $CDCl_3$, 298 K): 1.08 (s, 9H, CH_3), 1.41 (s, 9H, CH_3), 2.05 (s, 3H, CH_3), 3.03-3.16 (m, 2H, CH_2), 3.17-3.31 (m, 2H, CH_2), 6.13 (dd, $^3J_{P\text{-}H}$ = 14.1 Hz, $^4J_{H\text{-}H}$ = 2.6 Hz, 1H, H5), 7.38 (d, $^4J_{H\text{-}H}$ = 2.6 Hz, 1H, H3), 7.42-7.62 (m, 10H, Ar-*H*). ^{13}C{^{1}H} NMR (δ in ppm, 75 MHz, $CDCl_3$, 298 K): 20.5 (d, $^1J_{P\text{-}C}$ = 63.8 Hz, *C*H$_2$), 29.1 (s, *C*H$_3$), 29.9 (s, *C*H$_3$), 31.5 (s, *C*H$_3$), 34.0 (d, $^4J_{P\text{-}C}$ = 1.7 Hz, *C*CH$_3$), 35.1 (d, $^4J_{P\text{-}C}$ = 2.5 Hz, *C*CH$_3$), 38.1 (d, $^2J_{P\text{-}C}$ = 2.8 Hz, *C*H$_2$), 96.9 (d, $^1J_{P\text{-}C}$ = 99.9 Hz, C6), 125.5 (d, $^1J_{P\text{-}C}$ = 86.0 Hz, $C_{i\text{-}Ph}$), 126.7 (d, $^2J_{P\text{-}C}$ = 12.7 Hz, C5), 129.1 (d, $^3J_{P\text{-}C}$ = 11.8 Hz, C4), 130.6 (d, $^4J_{P\text{-}C}$ = 1.9 Hz, C3), 131.7 (d, $^2J_{P\text{-}C}$ = 14.9 Hz, $C_{o\text{-}Ph}$), 132.4 (d, $^3J_{P\text{-}C}$ = 3.0 Hz, $C_{m\text{-}Ph}$), 132.9 (d, $^3J_{P\text{-}C}$ = 9.1 Hz, C2), 140.0 (d, $^4J_{P\text{-}C}$ = 8.3 Hz, $C_{p\text{-}Ph}$), 174.8 (d, $^2J_{P\text{-}C}$ = 4.4 Hz, C1), 207.0 (d, $^3J_{P\text{-}C}$ = 13.2 Hz, CO). ^{31}P{^{1}H} NMR (δ in ppm, 162 MHz, $CDCl_3$, 298 K): 20.7. UV-Vis ($CHCl_3$): λ_{max} = 363 nm (ε = 5.44 * 10^3 L mol^{-1} cm^{-1}).

2d. 0.21 mmol (18.1 mg) methyl acrylate was used. The product was recrystallized from hot hexane (yellow solid, Yield: 44.0 mg, 46%). ^{1}H NMR (δ in ppm, 300 MHz, $CDCl_3$, 298 K): 1.10 (s, 9H, CH_3), 1.41 (s, 9H, CH_3), 2.81-2.98 (m, 2H, CH_2), 3.26-3.44 (m, 2H, CH_2), 3.62 ppm (s, 3H, CH_3), 6.20 (dd, $^3J_{P\text{-}H}$ = 13.9 Hz, $^4J_{H\text{-}H}$ = 1.9 Hz, 1H, H5), 7.39 (d, $^4J_{H\text{-}H}$ = 2.1 Hz, 1H, H3), 7.43-7.67 (m, 10H, Ar-*H*). ^{13}C{^{1}H} NMR (δ in

ppm, 75 MHz, $CDCl_3$, 298 K): 21.3 (d, $^1J_{P-C}$ = 63.9 Hz, CH_2), 28.6 (d, $^2J_{P-C}$ = 1.7 Hz, CH_2), 29.0 (s, CH_3), 31.5 (s, CH_3), 33.9 (d, $^4J_{P-C}$ = 1.4 Hz, CCH_3), 35.0 (d, $^4J_{P-C}$ = 2.1 Hz, CCH_3), 52.0 (s, O-CH_3), 96.1 (d, $^1J_{P-C}$ = 100.5 Hz, C6), 125.0 (d, $^1J_{P-C}$ = 86.9 Hz, C_{i-Ph}), 126.3 (d, $^2J_{P-C}$ = 13.1 Hz, C5), 129.0 (d, $^3J_{P-C}$ = 11.9 Hz, C4), 130.5 (d, $^4J_{P-C}$ = 1.2 Hz, C3), 131.5 (d, $^2J_{P-C}$ = 15.1 Hz, C_{o-Ph}), 132.4 (d, $^3J_{P-C}$ = 2.7 Hz, C_{m-Ph}), 132.8 (d, $^3J_{P-C}$ = 9.3 Hz, C2), 140.0 (d, $^4J_{P-C}$ = 8.1 Hz, C_{p-Ph}), 172.7 (d, $^3J_{P-C}$ = 18.1 Hz, CO), 174.9 (d, $^2J_{P-C}$ = 4.4 Hz, C1). $^{31}P\{^1H\}$ NMR (δ in ppm, 162 MHz, $CDCl_3$, 298 K): 19.4. UV-Vis ($CHCl_3$): λ_{max} = 359 nm (ε = 5.28 * 10^3 L mol^{-1} cm^{-1}).

2e. 0.21 mmol (21.0 mg) ethyl acrylate was used. The product was recrystallized from hot hexane (yellow solid, Yield: 48.5 mg, 49%). ^1H NMR (δ in ppm, 300 MHz, $CDCl_3$, 298 K): 1.10 (s, 9H, CH_3), 1.19 (t, $^4J_{H-H}$ = 7.1 Hz), 1.41 (s, 9H, CH_3), 2.79-2.95 (m, 2H, CH_2), 3.25-3.42 (m, 2H, CH_2), 4.08 (q, $^4J_{H-H}$ = 7.1 Hz), 6.19 (dd, $^3J_{P-H}$ = 13.9 Hz, $^4J_{H-H}$ = 2.3 Hz, 1H, H5), 7.38 (d, $^4J_{H-H}$ = 2.3 Hz, 1H, H3), 7.43-7.65 (m, 10H, Ar-*H*). $^{13}C\{^1H\}$ NMR (δ in ppm, 75 MHz, $CDCl_3$, 298 K): 14.2 (s, CH_3), 21.3 (d, $^1J_{P-C}$ = 63.8 Hz, CH_2), 28.9 (d, $^2J_{P-C}$ = 1.9 Hz, CH_2), 29.1 (s, CH_3), 31.5 (s, CH_3), 34.0 (d, $^4J_{P-C}$ = 1.4 Hz, CCH_3), 35.1 (d, $^4J_{P-C}$ = 2.1 Hz, CCH_3), 61.1 (O-CH_2), 96.2 (d, $^1J_{P-C}$ = 100.5 Hz, C6), 125.2 (d, $^1J_{P-C}$ = 86.9 Hz, C_{i-Ph}), 126.4 (d, $^2J_{P-C}$ = 12.6 Hz, C5), 129.1 (d, $^3J_{P-C}$ = 11.9 Hz, C4), 130.6 (d, $^4J_{P-C}$ = 1.3 Hz, C3), 131.6 (d, $^2J_{P-C}$ = 15.1 Hz, C_{o-Ph}), 132.5 (d, $^3J_{P-C}$ = 2.8 Hz, C_{m-Ph}), 132.9 (d, $^3J_{P-C}$ = 9.3 Hz, C2), 140.1 (d, $^4J_{P-C}$ = 8.1 Hz, C_{p-Ph}), 172.4 (d, $^3J_{P-C}$ = 17.9 Hz, CO), 174.9 (d, $^2J_{P-C}$ = 4.2 Hz, C1). $^{31}P\{^1H\}$ NMR (δ in ppm, 162 MHz, $CDCl_3$, 298 K): 19.4. UV-Vis ($CHCl_3$): λ_{max} = 361 nm (ε = 5.97 * 10^3 L mol^{-1} cm^{-1}).

2f. 0.21 mmol (26.9 mg) *tert*-butyl acrylate was used. The product was recrystallized from hot toluene (yellow crystals, Yield: 78.2 mg, 75%). ^1H NMR (δ in ppm, 300 MHz, $CDCl_3$, 298 K): 1.10 (s, 9H, CH_3), 1.39 (s, CH_3), 1.41 (s, 9H, CH_3), 2.69-2.87 (m, 2H, CH_2), 3.20-3.38 (m, 2H, CH_2), 6.21 (dd, $^3J_{P-H}$ = 13.9 Hz, $^4J_{H-H}$ = 1.9 Hz, 1H, H5), 7.38 (d, $^4J_{H-H}$ = 2.1 Hz, 1H, H3), 7.43-7.52 (m, 4H, Ar-*H*), 7.52-7.65 (m, 6H, Ar-*H*). $^{13}C\{^1H\}$ NMR (δ in ppm, 75 MHz, $CDCl_3$, 298 K): 21.3 (d, $^1J_{P-C}$ = 63.3 Hz, CH_2), 28.0 (s, CH_3), 29.0 (s, CH_3), 29.8 (d, $^2J_{P-C}$ = 1.9 Hz, CH_2), 31.5 (s, CH_3), 33.9 (d, $^4J_{P-C}$ = 1.3 Hz, CCH_3), 35.0 (d, $^4J_{P-C}$ = 2.1 Hz, CCH_3), 81.2 (s, O-CCH_3), 96.3 (d, $^1J_{P-C}$ = 100.3 Hz, C6), 125.1 (d, $^1J_{P-C}$ = 86.8 Hz, C_{i-Ph}), 126.4 (d, $^2J_{P-C}$ = 12.5 Hz, C5), 129.0 (d, $^3J_{P-C}$ = 11.9 Hz, C4), 130.5 (d, $^4J_{P-C}$ = 1.2 Hz, C3), 131.3 (d, $^2J_{P-C}$ = 15.1 Hz, C_{o-Ph}), 132.3 (d, $^3J_{P-C}$ = 2.7 Hz, C_{m-Ph}), 132.8 (d, $^3J_{P-C}$ = 9.3 Hz, C2), 140.0 (d, $^4J_{P-C}$ = 8.1 Hz, C_{p-Ph}), 171.5 (d, $^3J_{P-C}$ = 18.1 Hz, CO), 174.8 (d, $^2J_{P-C}$ = 4.4 Hz, C1). $^{31}P\{^1H\}$ NMR (δ in ppm, 162 MHz, $CDCl_3$, 298 K): 19.6. UV-Vis ($CHCl_3$): λ_{max} = 360 nm (ε = 5.38 * 10^3 L mol^{-1} cm^{-1}).

2g. 0.21 mmol (34.0 mg) benzyl acrylate was used. The product was recrystallized via vapor diffusion of pentane on a saturated solution in toluene (yellow solid, Yield: 47.3 mg, 43%). ^1H NMR (δ in ppm, 300 MHz, $CDCl_3$, 298 K): 1.09 (s, 9H, CH_3), 1.41 (s, 9H, CH_3), 2.86-3.01 (m, 2H, CH_2), 3.28-3.43 (m, 2H, CH_2), 5.06 (s, 2H, Ph-CH_2), 6.19 (dd, $^3J_{P-H}$ = 13.9 Hz, $^4J_{H-H}$ = 2.1 Hz, 1H, H5), 7.24-7.35 (m, 5H, Ar-*H*), 7.38 (d, $^4J_{H-H}$ = 2.1 Hz, 1H, H3), 7.42-7.63 (m, 10H, Ar-*H*). $^{13}C\{^1H\}$ NMR (δ in ppm, 75 MHz, $CDCl_3$, 298 K): 21.2 (d, $^1J_{P-C}$ = 63.9 Hz, CH_2), 28.8 (d, $^2J_{P-C}$ = 1.6 Hz, CH_2), 28.9 (s, CH_3), 31.4 (s, CH_3), 33.8 (d, $^4J_{P-C}$ = 1.4 Hz, CCH_3), 34.9 (d, $^4J_{P-C}$ = 2.1 Hz, CCH_3), 66.8 (s, O-CH_2), 96.1 (d, $^1J_{P-C}$ = 100.4 Hz, C6), 125.0 (d, $^1J_{P-C}$ = 86.9 Hz, C_{i-Ph}), 126.3 (d, $^2J_{P-C}$ = 12.6 Hz, C5), 128.2 (s, Ar-*C*), 128.3 (s, Ar-*C*), 128.5 (s, Ar-*C*), 129.0 (d, $^3J_{P-C}$ = 11.9 Hz, C4), 130.5 (d, $^4J_{P-C}$ = 1.2 Hz, C3), 131.5 (d, $^2J_{P-C}$ = 15.1 Hz, C_{o-Ph}), 132.4 (d, $^3J_{P-C}$ = 2.7 Hz, C_{m-Ph}), 132.7 (d, $^3J_{P-C}$ = 9.3 Hz, C2), 135.5 (s, Ar-*C*), 139.9 (d, $^4J_{P-C}$ = 8.1 Hz, C_{p-Ph}), 172.1 (d, $^3J_{P-C}$ = 18.1 Hz, CO), 174.8 (d, $^2J_{P-C}$ = 4.4 Hz, C1). $^{31}P\{^1H\}$ NMR (δ in ppm, 162 MHz, $CDCl_3$, 298 K): 19.5. UV-Vis ($CHCl_3$): λ_{max} = 362 nm (ε = 5.49 * 10^3 L mol^{-1} cm^{-1}).

2h. 0.21 mmol (24.3 mg) hydroxyethyl acrylate was used. The product was recrystallized from hot toluene (off-white powder, Yield: 62.1 mg, 61%). ^1H NMR (δ in ppm, 300 MHz, $CDCl_3$, 298 K): 1.08 (s,

9H, CH_3), 1.40 (s, 9H, CH_3), 2.82-2.98 (m, 2H, CH_2), 3.33-3.48 (m, 2H, CH_2), 3.54 (t, $^3J_{H-H}$ = 4.5 Hz), 4.03 (t, $^3J_{H-H}$ = 4.5 Hz), 6.15 (dd, $^3J_{P-H}$ = 14.1 Hz, $^4J_{H-H}$ = 2.1 Hz, 1H, H5), 7.40 (d, $^4J_{H-H}$ = 2.1 Hz, 1H, H3), 7.46-7.65 (m, 10H, Ar-*H*). $^{13}C\{^1H\}$ NMR (δ in ppm, 75 MHz, $CDCl_3$, 298 K): 20.9 (d, $^1J_{P-C}$ = 62.9 Hz, *C*H_2), 29.0 (s, *C*H_3), 31.4 (s, *C*H_3), 33.9 (d, $^4J_{P-C}$ = 1.2 Hz, *C*CH_3), 34.9 (d, $^4J_{P-C}$ = 2.1 Hz, *C*CH_3), 60.2 (s, O-*C*H_2). 66.8 (s, O-*C*H_2), 96.8 (d, $^1J_{P-C}$ = 100.3 Hz, C6), 124.5 (d, $^1J_{P-C}$ = 86.4 Hz, C_{i-Ph}), 126.5 (d, $^2J_{P-C}$ = 12.5 Hz, C5), 129.1 (d, $^3J_{P-C}$ = 11.9 Hz, C4), 130.8 (d, $^4J_{P-C}$ = 1.2 Hz, C3), 132.2 (d, $^2J_{P-C}$ = 15.0 Hz, C_{o-Ph}), 132.6 (d, $^3J_{P-C}$ = 2.8 Hz, C_{m-Ph}), 133.0 (d, $^3J_{P-C}$ = 9.3 Hz, C2), 140.1 (d, $^4J_{P-C}$ = 8.0 Hz, C_{p-Ph}), 171.8 (d, $^3J_{P-C}$ = 14.7 Hz, CO), 173.9 (d, $^2J_{P-C}$ = 4.0 Hz, C1). $^{31}P\{^1H\}$ NMR (δ in ppm, 162 MHz, $CDCl_3$, 298 K): 20.9. UV-Vis ($CHCl_3$): λ_{max} = 359 nm (ε = 5.80 * 10^3 L mol^{-1} cm^{-1}).

2i. 0.21 mmol (22.3 mg) methyl vinyl sulfone was used. The product was recrystallized from hot cyclohexane (light brown solid, Yield: 17.8 mg, 18%). ^1H NMR (δ in ppm, 300 MHz, $CDCl_3$, 298 K): 1.09 (s, 9H, CH_3), 1.41 (s, 9H, CH_3), 3.00 (s, 3H, S-CH_3), 3.39-3.65 (m, 4H, CH_2-CH_2), 6.14 (dd, $^3J_{P-H}$ = 14.1 Hz, $^4J_{H-H}$ = 2.2 Hz, 1H, H5), 7.43 (d, $^4J_{H-H}$ = 2.2 Hz, 1H, H3), 7.47-7.60 (m, 8H, Ar-*H*), 7.61-7.70 (m, 2H, Ar-*H*). $^{13}C\{^1H\}$ NMR (δ in ppm, 75 MHz, $CDCl_3$, 298 K): 21.2 (d, $^1J_{P-C}$ = 62.6 Hz, *C*H_2), 29.0 (s, *C*H_3), 31.3 (s, *C*H_3), 33.9 (d, $^4J_{P-C}$ = 1.4 Hz, *C*CH_3), 35.0 (d, $^4J_{P-C}$ = 2.1 Hz, *C*CH_3), 40.2 (s, S-*C*H_3), 50.2 (d, $^2J_{P-C}$ = 2.0 Hz, *C*H_2), 95.3 (d, $^1J_{P-C}$ = 97.5 Hz, C6), 124.0 (d, $^1J_{P-C}$ = 87.0 Hz, C_{i-Ph}), 126.5 (d, $^2J_{P-C}$ = 13.1 Hz, C5), 129.5 (d, $^3J_{P-C}$ = 12.1 Hz, C4), 131.4 (d, $^4J_{P-C}$ = 1.1 Hz, C3), 132.6 (d, $^3J_{P-C}$ = 9.5 Hz, C2), 133.0 (d, d, $^3J_{P-C}$ = 2.7 Hz, C_{m-Ph}), 140.2 (d, $^4J_{P-C}$ = 8.1 Hz, C_{p-Ph}), 175.0 (s, C1). $^{31}P\{^1H\}$ NMR (δ in ppm, 162 MHz, $CDCl_3$, 298 K): 19.7. UV-Vis ($CHCl_3$): λ_{max} = 359 nm (ε = 4.03 * 10^3 L mol^{-1} cm^{-1}).

*Methanol-d_4 assisted synthesis of **2a**.* Phosphine **1** (0.2 mmoL, 78 mg, 1 equiv) was dissolved in 0.3 mL diethyl ether in a 4 mL screw-cap vial. Acrylonitrile (0.3 mmoL, 15.9 mg, 1.5 equiv) and methanol-d_4 (1 mmol, 32 mg, 5 equiv) were dissolved in 0.3 mL diethyl ether in a separate vial. This solution was then added to the solution of **1** dropwise and stirred at room temperature for 24 h. Afterwards, the volatiles were removed in vacuo and the sample subjected to NMR analysis. A substantial decrease in the integral of the CH_2-CN group could be observed (1.3 instead of 2), thereby showing that this position is partially deuterated (see Figure S49).

Molecular Structure

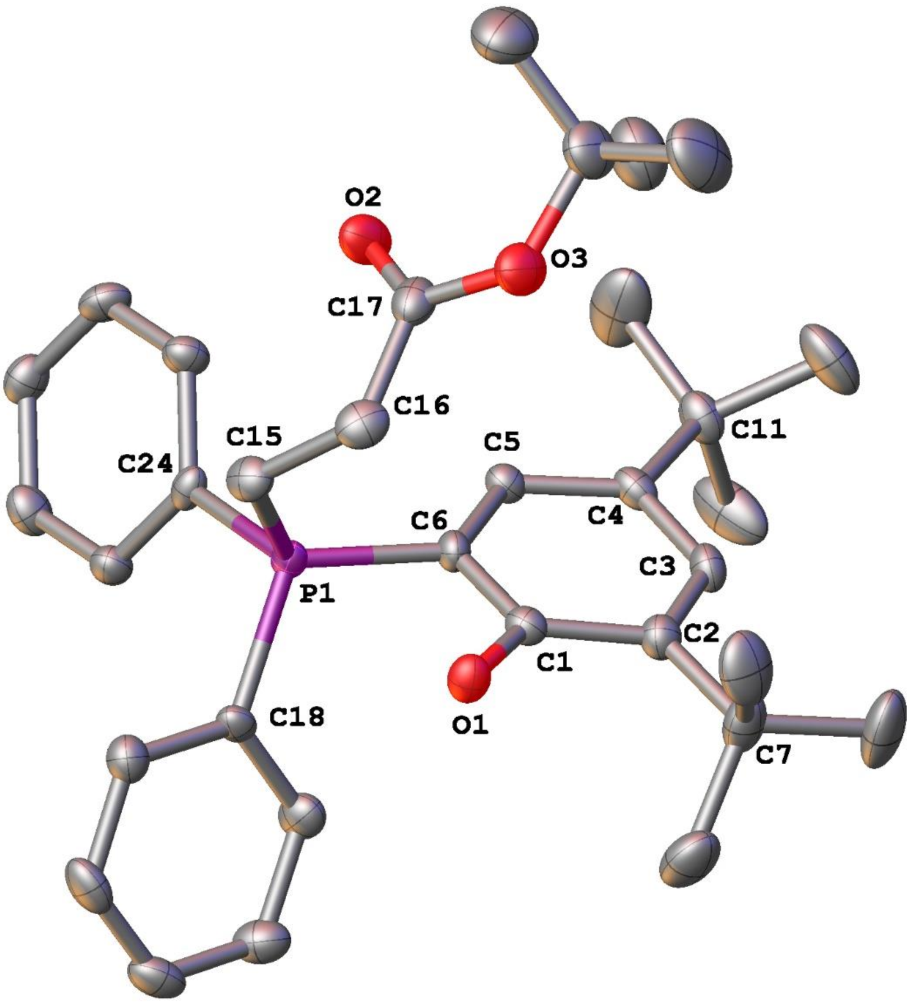

*Figure S1: Molecular structure of zwitterion **2f**, hydrogen atoms omitted for clarity, thermal ellipsoids drawn at 50% probability.*

Crystallographic Data

*Table S1: Crystallographic data for compounds **2a** and **2f***

	**2a**	**2f**
CCDC number	2287962	2287963
Empirical formula	$C_{29}H_{34}NOP$	$C_{33}H_{43}O_3P$
Formula weight	443.54	518.64
Temperature /K	150(2)	150(2)
Crystal system	monoclinic	monoclinic
Space group	*C2/c*	$P2_1/c$
a /Å	22.803(4)	14.510(3)
b /Å	12.656(2)	17.209(4)
c /Å	19.601(4)	12.229(3)
α /°	90	90
β /°	114.777(3)	95.933(3)
γ /°	90	90
Volume /Å^3	5147(2)	3037.3(11)
Z	8	4
ρ_{calc}g /cm^3	1.145	1.134
M /mm^{-1}	0.127	0.120
F(000)	1904	1120
Crystal size /mm^3	0.28 x 0.22 x 0.18	0.28 x 0.22 x 0.15
2Θ range for data collection /°	1.88 to 26.35	1.84 to 26.34
Index ranges	-28<=h<=28, -15<=k<=15, -24<=l<=24	-18<=h<=18, -21<=k<=21, -15<=l<=15
Reflections collected	13031	23787
Independent reflections	4547 [R(int) = 0.0345]	6191 [R(int) = 0.0349]
Data/restraints/parameters	4547 / 0 / 295	6191 / 0 / 343
Goodness-of-fit on F^2	1.093	1.338
Final R indexes [I>=2σ (I)]	R1 = 0.0559, wR2 = 0.1313	R1 = 0.0883, wR2 = 0.1676
Final R indexes [all data]	R1 = 0.0660, wR2 = 0.1374	R1 = 0.0912, wR2 = 0.1690
Largest diff. peak/hole / e Å^{-3}	0.422 and -0.186	0.515 and -0.439

NMR spectra

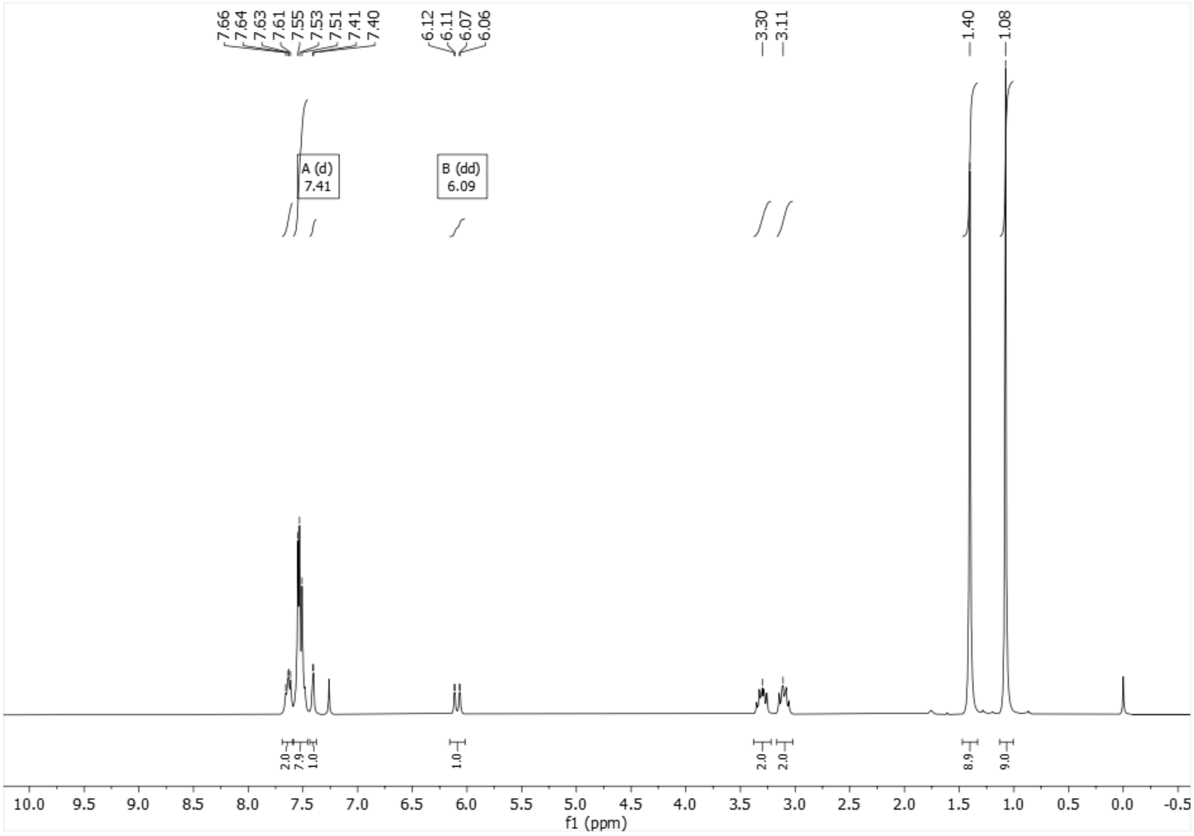

*Figure S2: ^{1}H NMR spectrum of **2a** recorded from a solution in $CDCl_3$ at 298 K.*

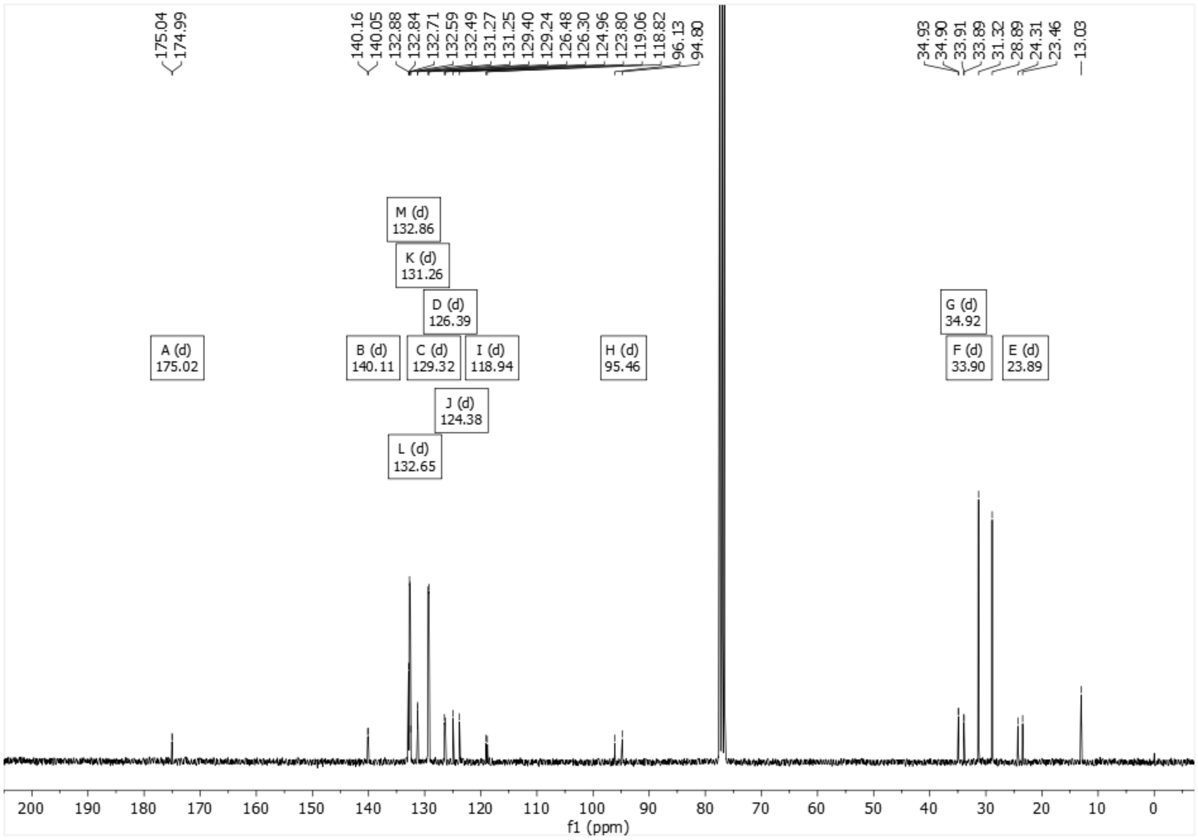

*Figure S3: ^{13}C{^{1}H} NMR spectrum of **2a** recorded from a solution in $CDCl_3$ at 298 K.*

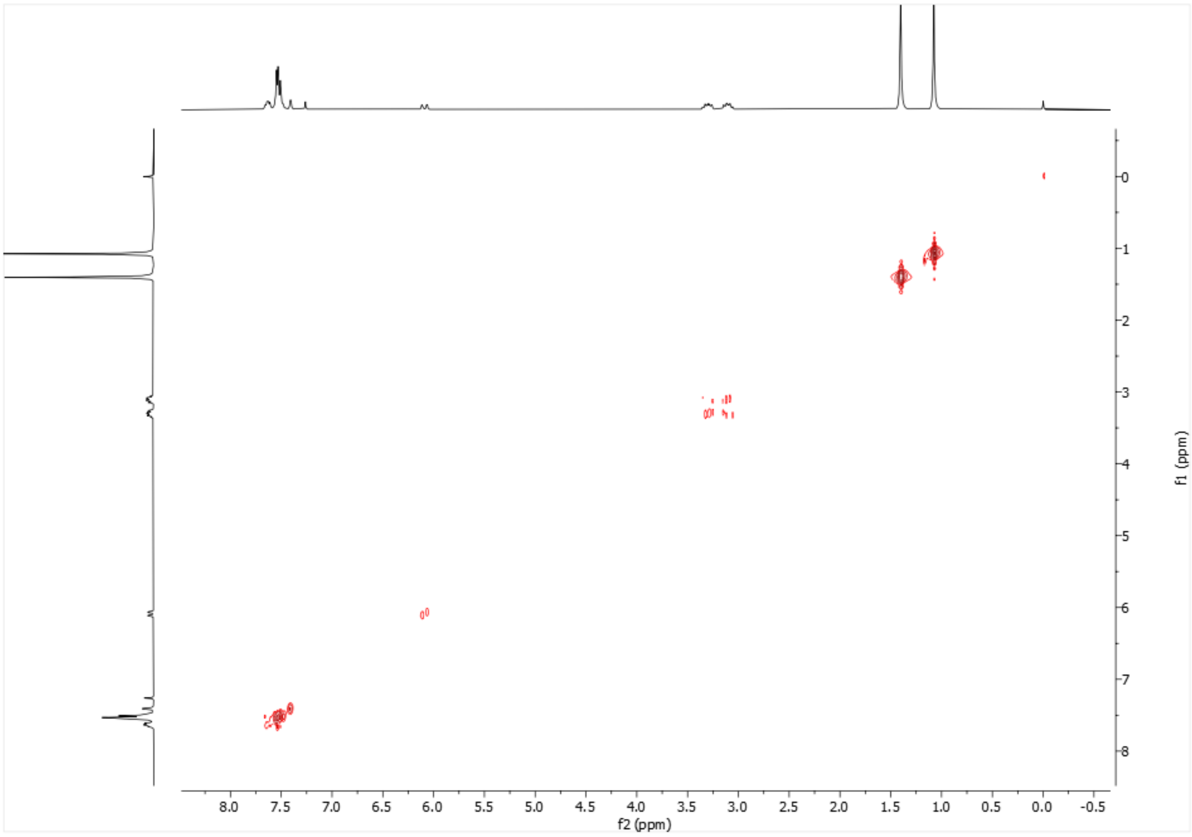

*Figure S4: ^{1}H-^{1}H COSY NMR spectrum of **2a** recorded from a solution in $CDCl_3$ at 298 K.*

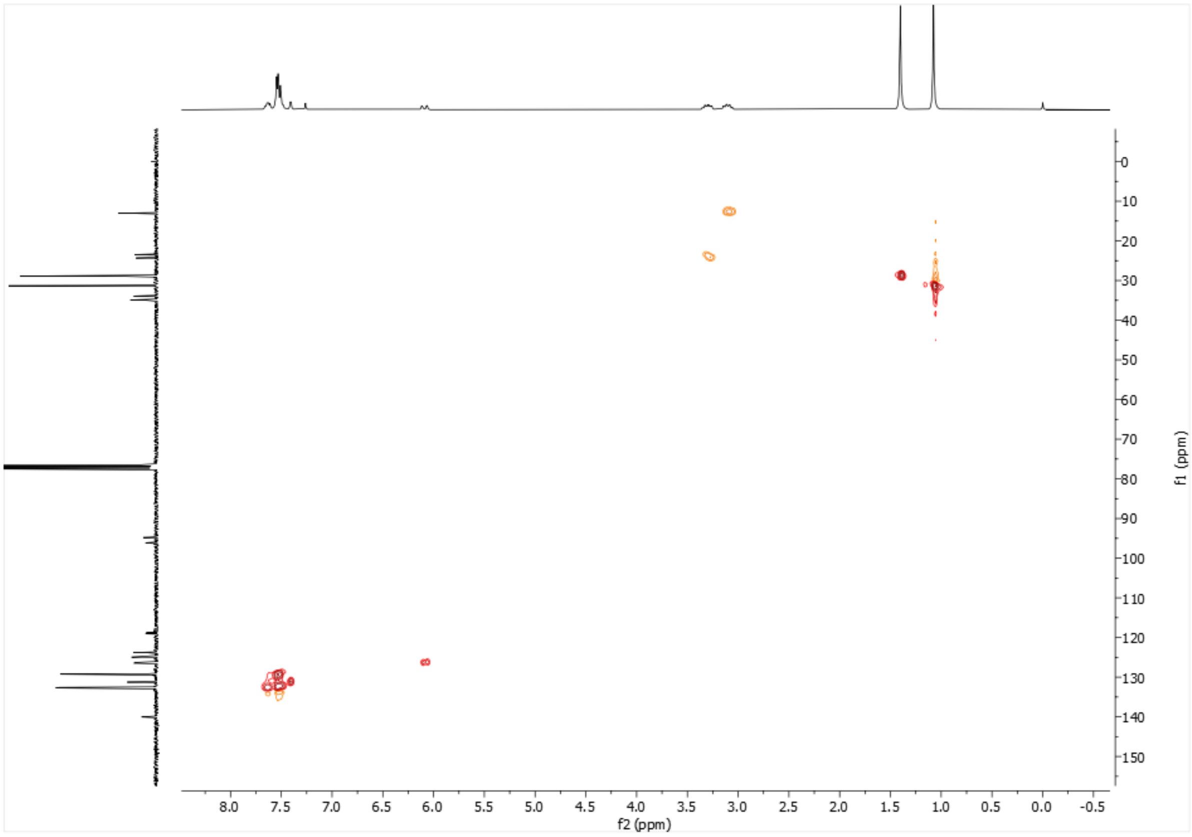

*Figure S5: ^{1}H-^{13}C HSQC NMR spectrum of **2a** recorded from a solution in $CDCl_3$ at 298 K.*

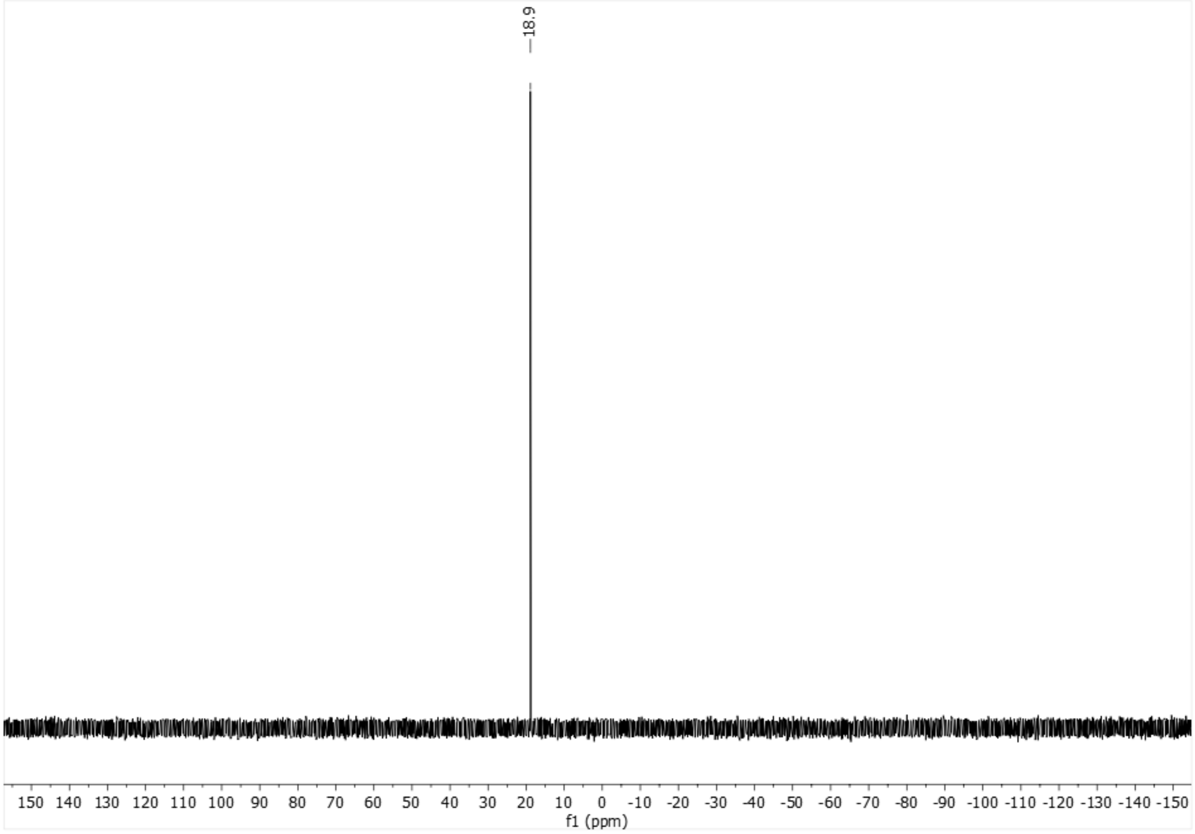

*Figure S6: $^{31}P\{^{1}H\}$ NMR spectrum of **2a** recorded from a solution in $CDCl_3$ at 298 K.*

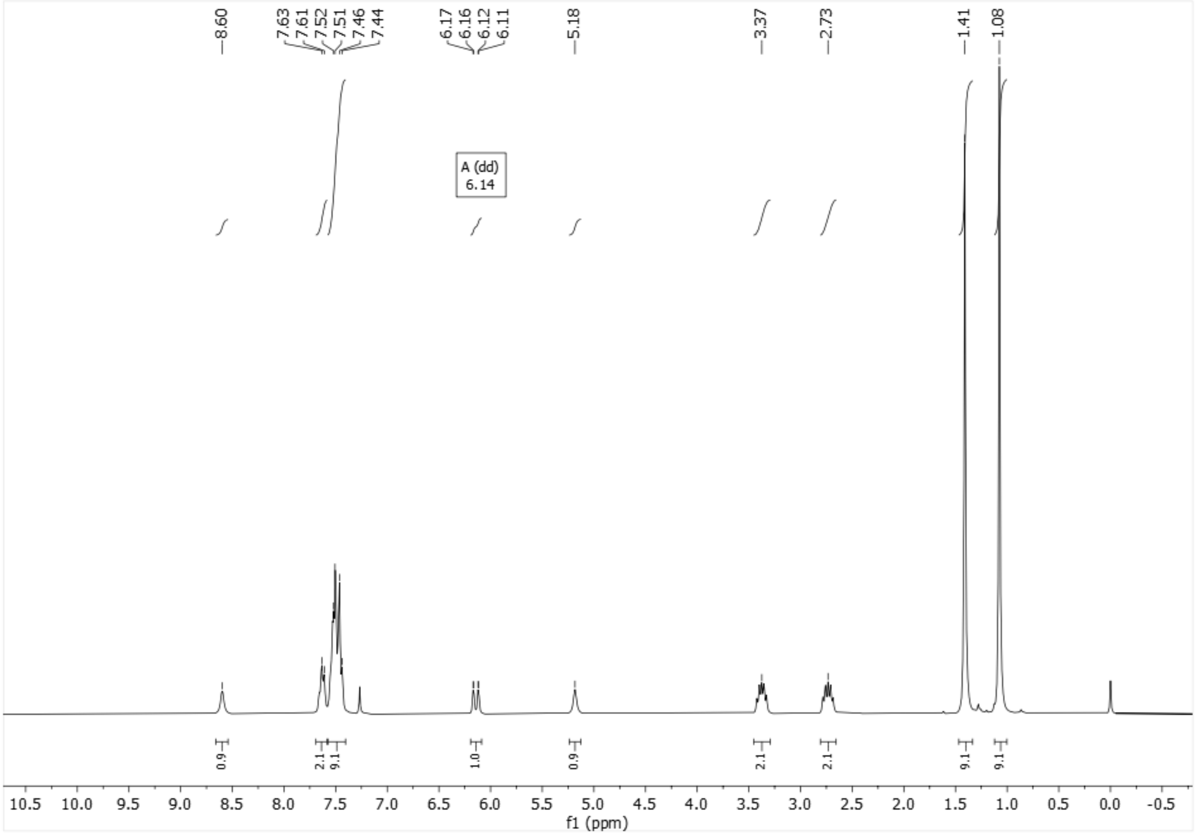

*Figure S7: ^{1}H NMR spectrum of **2b** recorded from a solution in $CDCl_3$ at 298 K.*

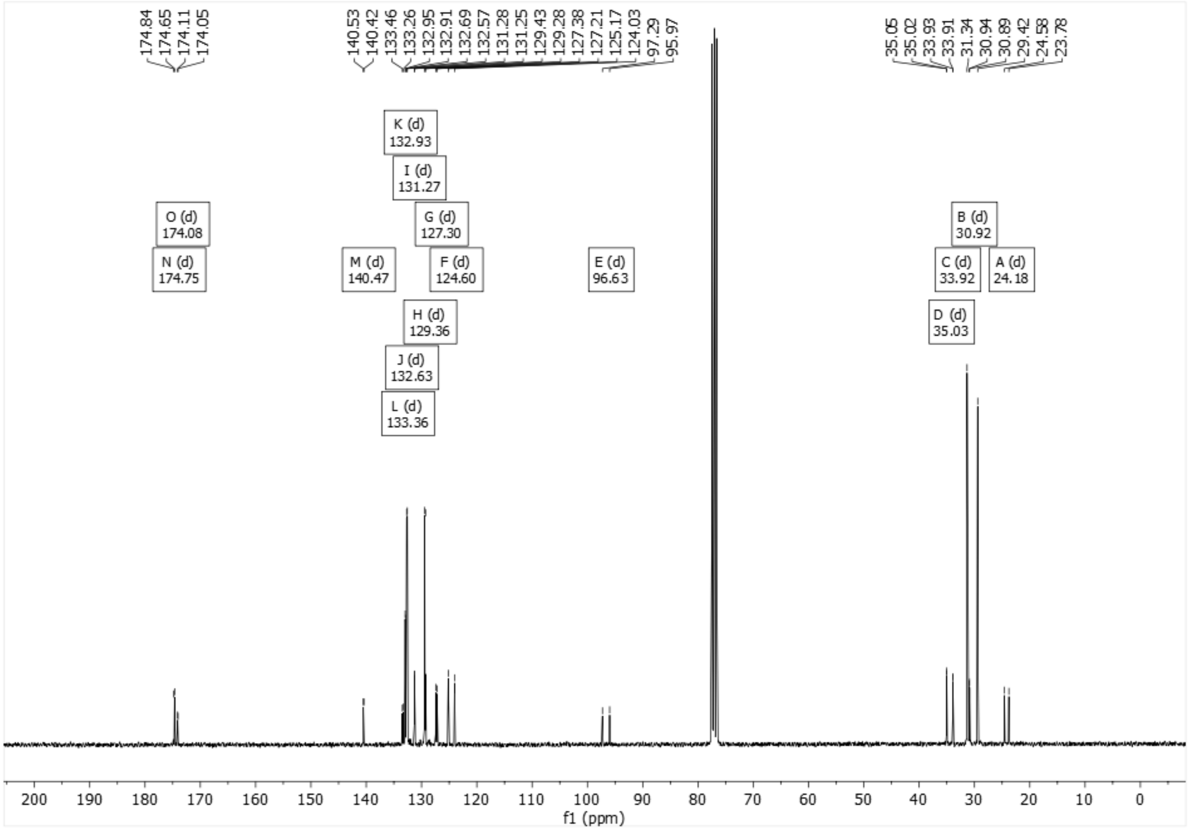

*Figure S8: $^{13}C\{^1H\}$ NMR spectrum of **2b** recorded from a solution in $CDCl_3$ at 298 K.*

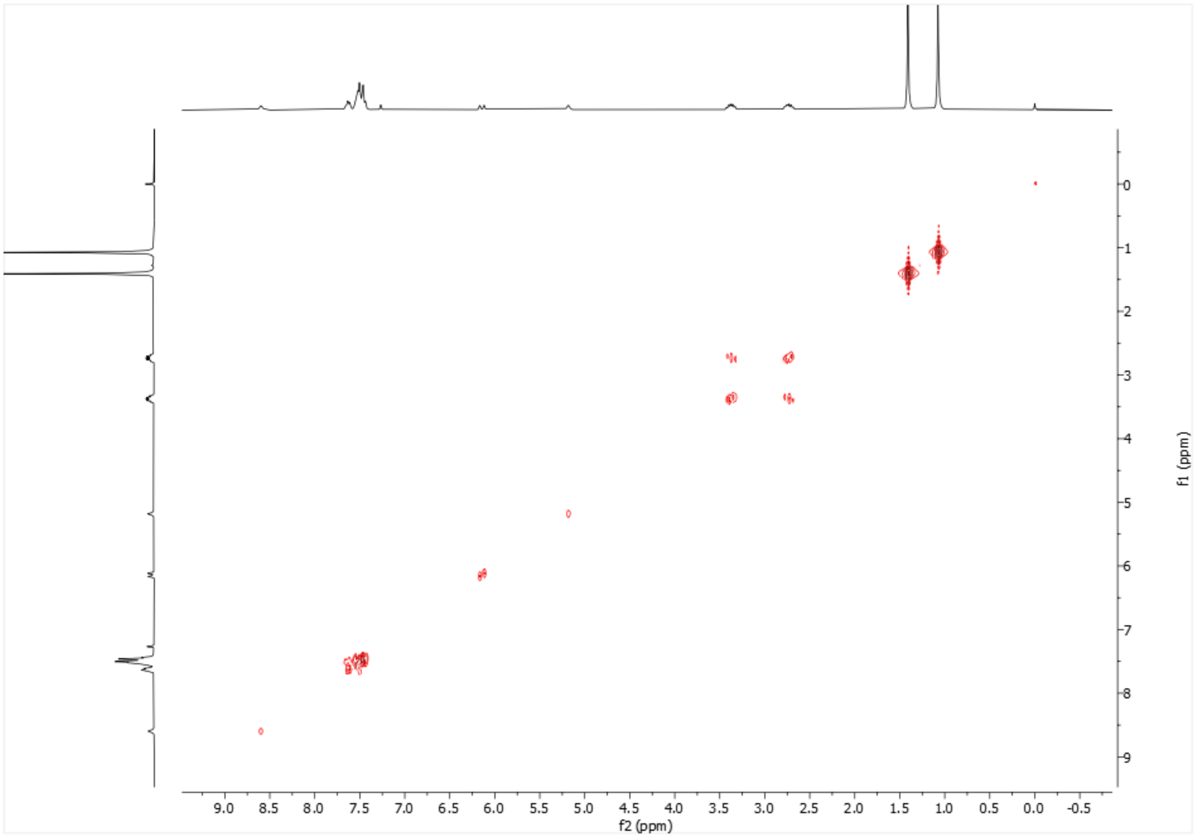

*Figure S9: ^1H-^1H COSY NMR spectrum of **2b** recorded from a solution in $CDCl_3$ at 298 K.*

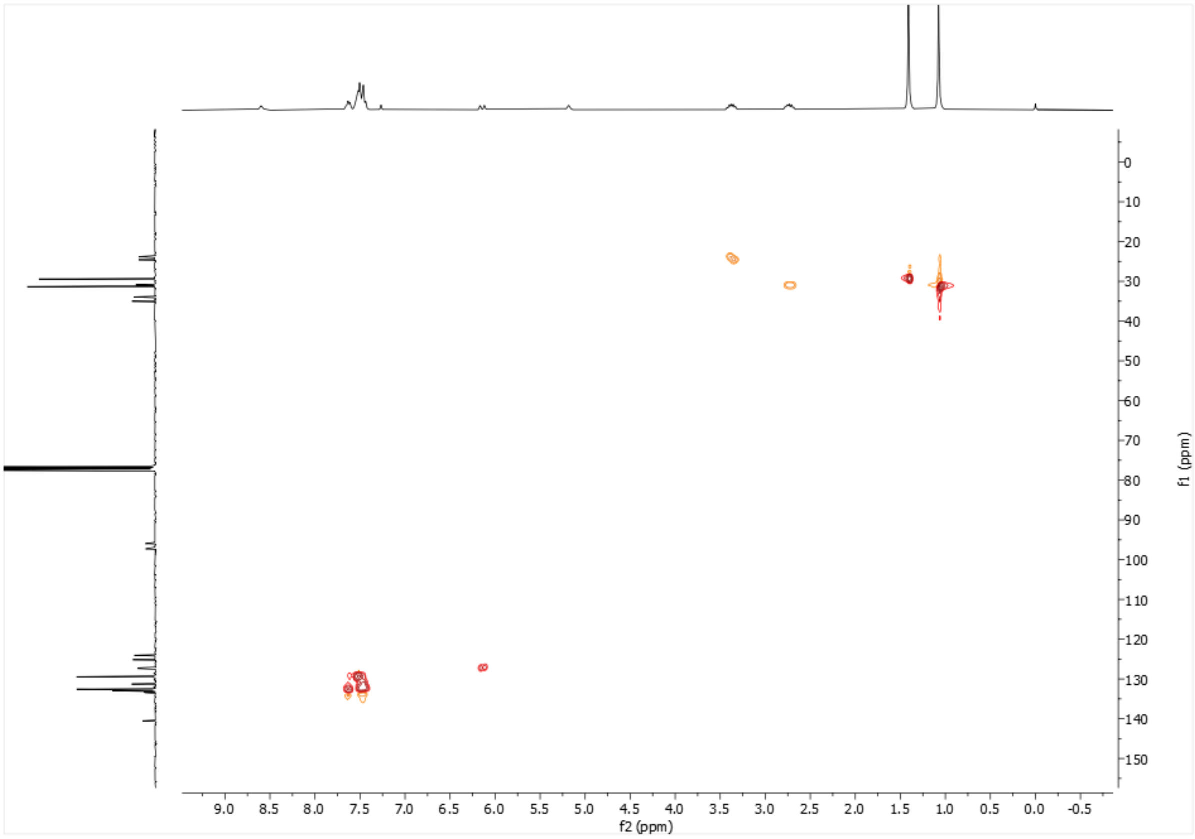

*Figure S10: ^{1}H-^{13}C HSQC NMR spectrum of **2b** recorded from a solution in $CDCl_3$ at 298 K.*

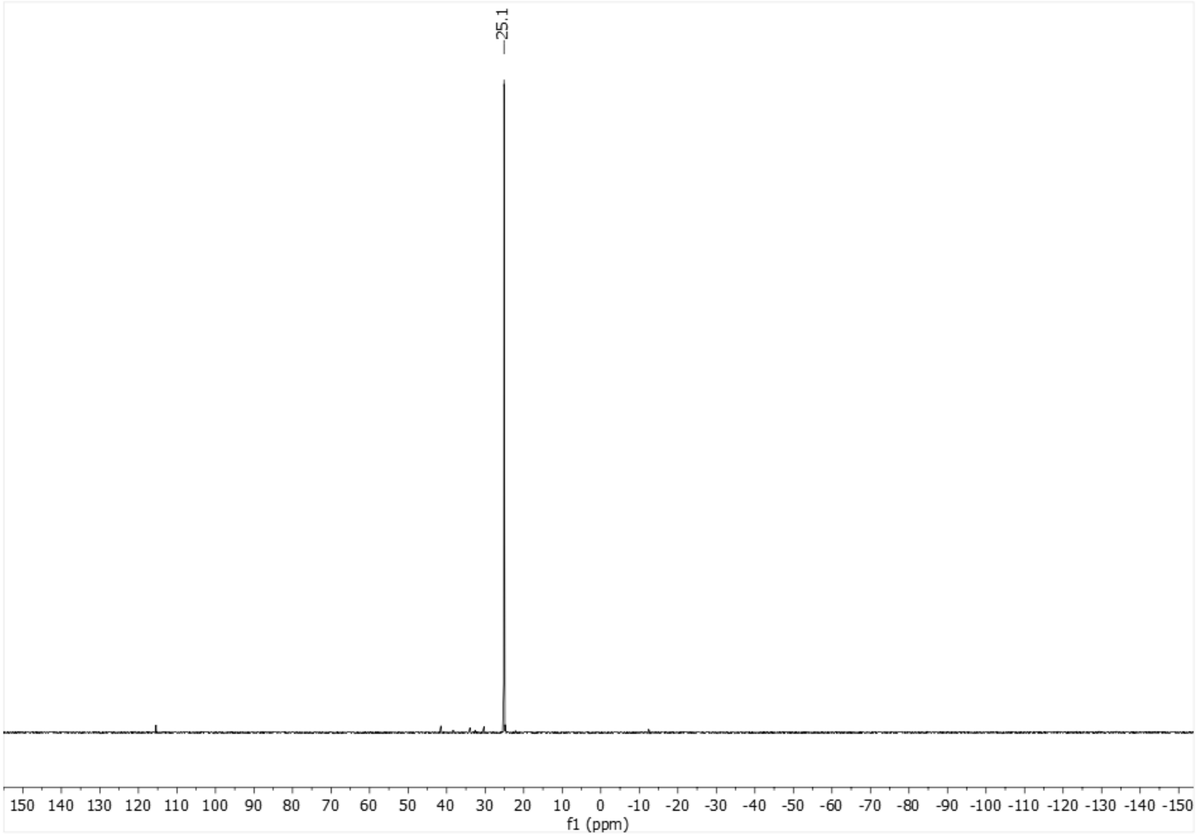

*Figure S11: $^{31}P\{^{1}H\}$ NMR spectrum of **2b** recorded from a solution in $CDCl_3$ at 298 K.*

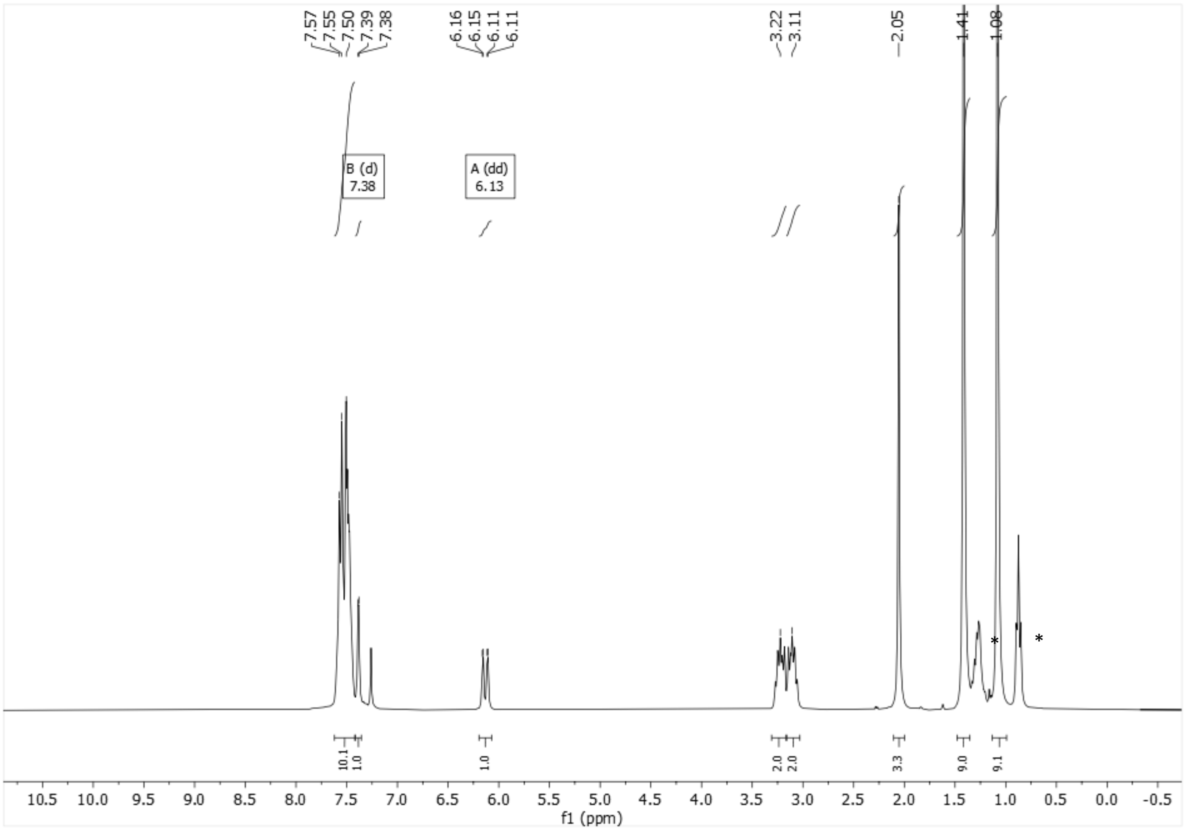

*Figure S12: ^{1}H NMR spectrum of **2c** recorded from a solution in $CDCl_3$ at 298 K. Trace impurities are marked with an asterisk.*

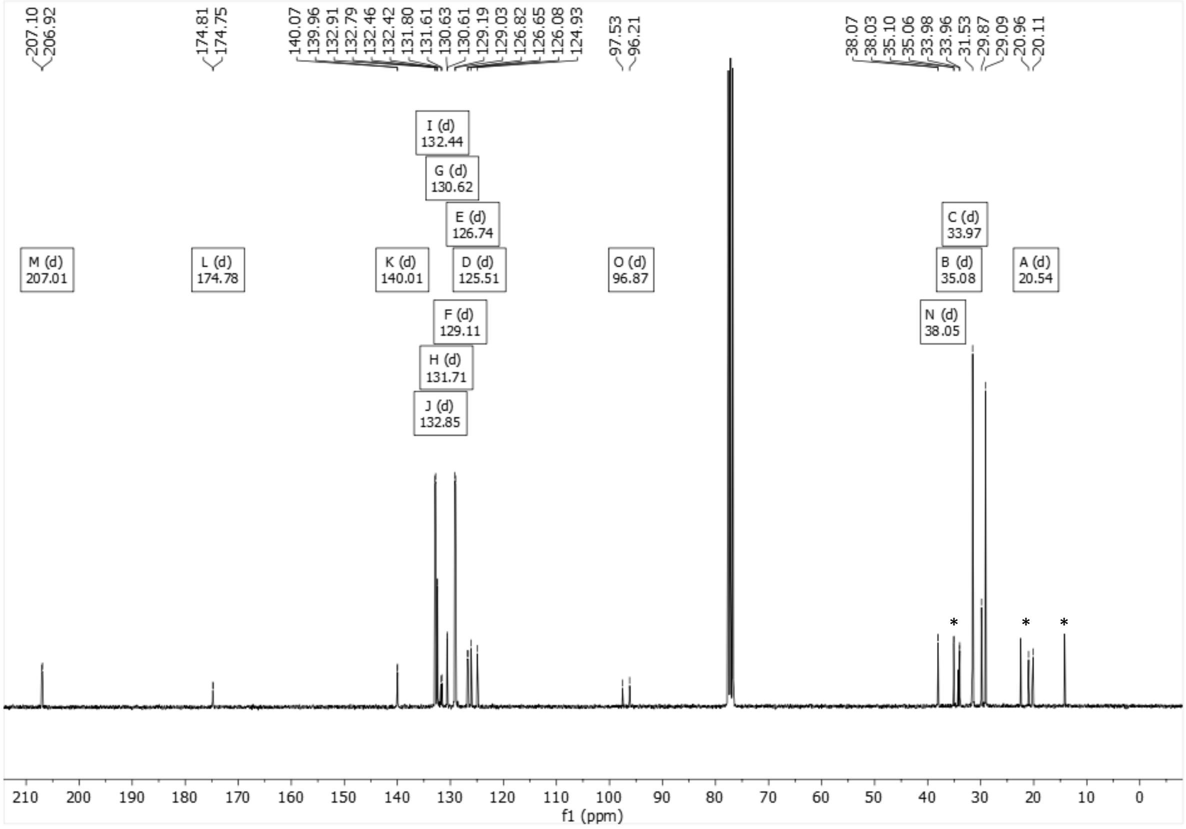

*Figure S13: ^{13}C{^{1}H} NMR spectrum of **2c** recorded from a solution in $CDCl_3$ at 298 K. Trace impurities are marked with an asterisk.*

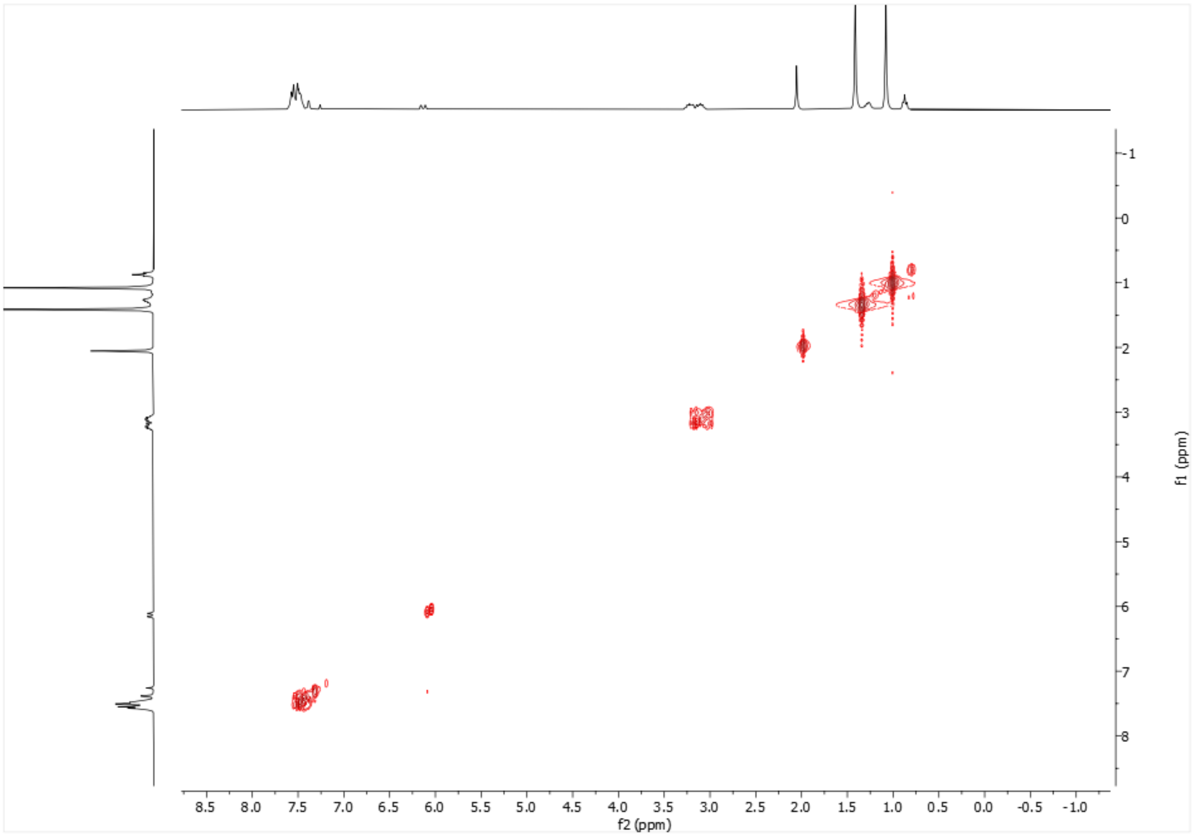

*Figure S14: ^{1}H-^{1}H COSY NMR spectrum of **2c** recorded from a solution in $CDCl_3$ at 298 K.*

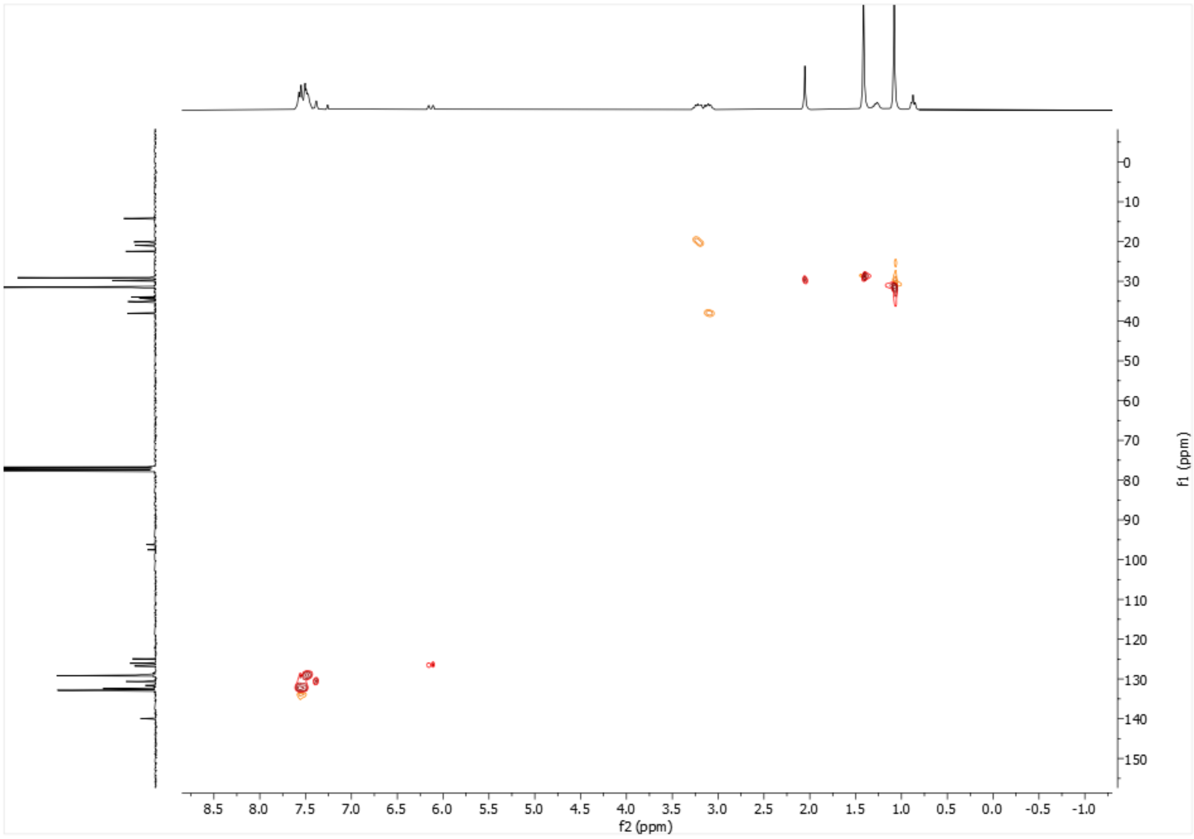

*Figure S15: ^{1}H-^{13}C HSQC NMR spectrum of **2c** recorded from a solution in $CDCl_3$ at 298 K.*

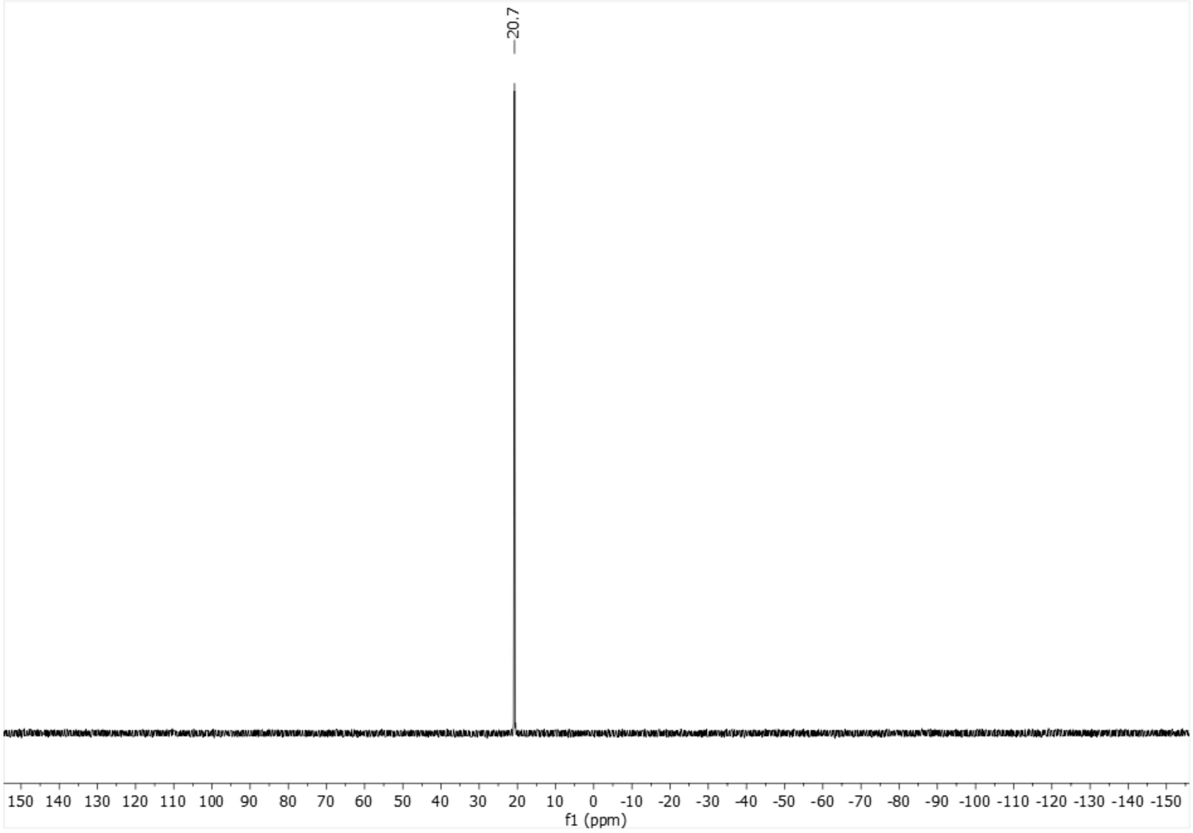

*Figure S16: $^{31}P\{^{1}H\}$ NMR spectrum of **2c** recorded from a solution in $CDCl_3$ at 298 K.*

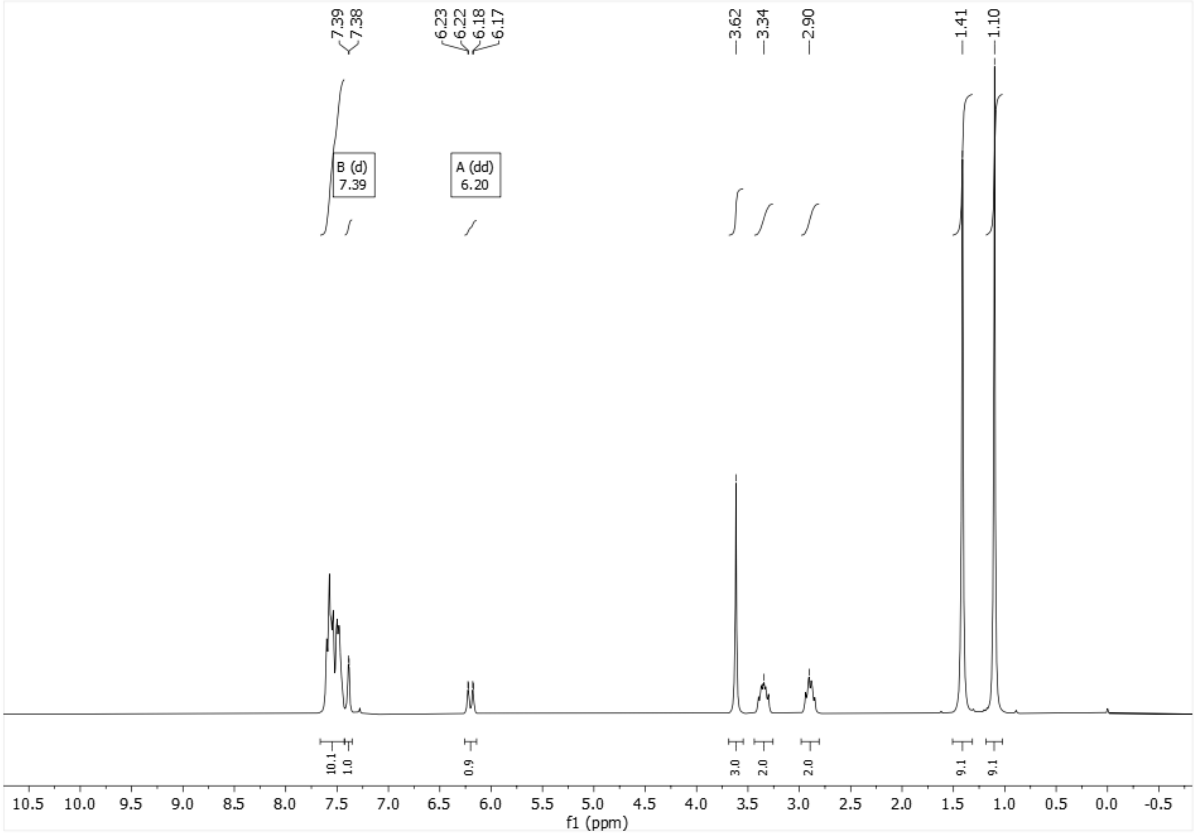

*Figure S17: ^{1}H NMR spectrum of **2d** recorded from a solution in $CDCl_3$ at 298 K.*

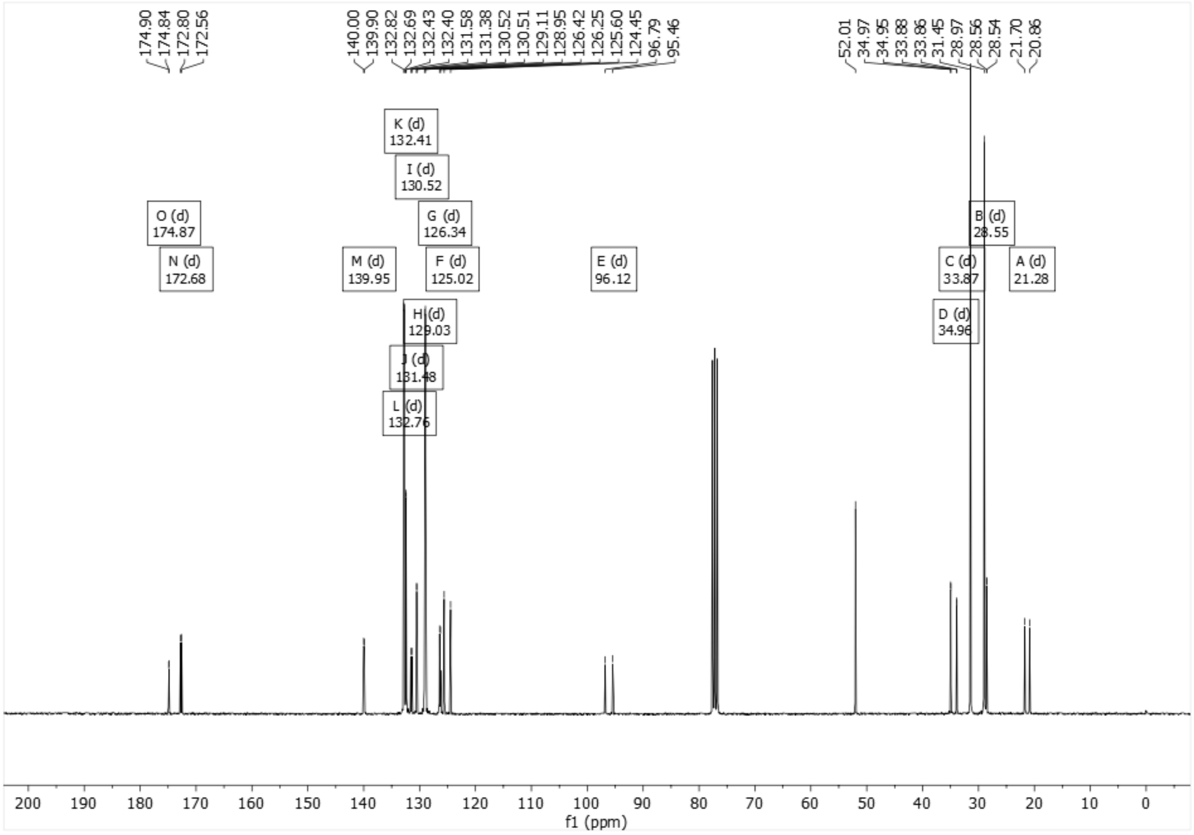

*Figure S18: $^{13}C\{^{1}H\}$ NMR spectrum of **2d** recorded from a solution in $CDCl_3$ at 298 K.*

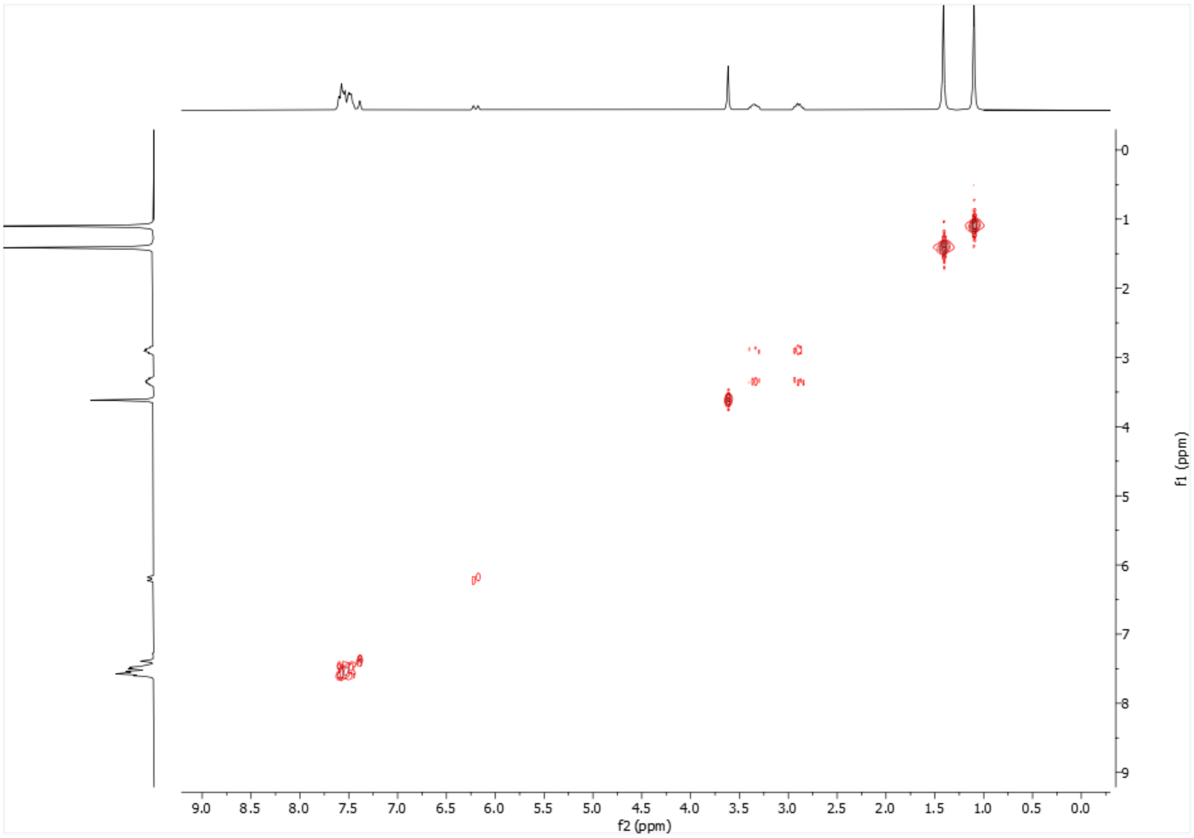

*Figure S19: ^{1}H-^{1}H COSY NMR spectrum of **2d** recorded from a solution in $CDCl_3$ at 298 K.*

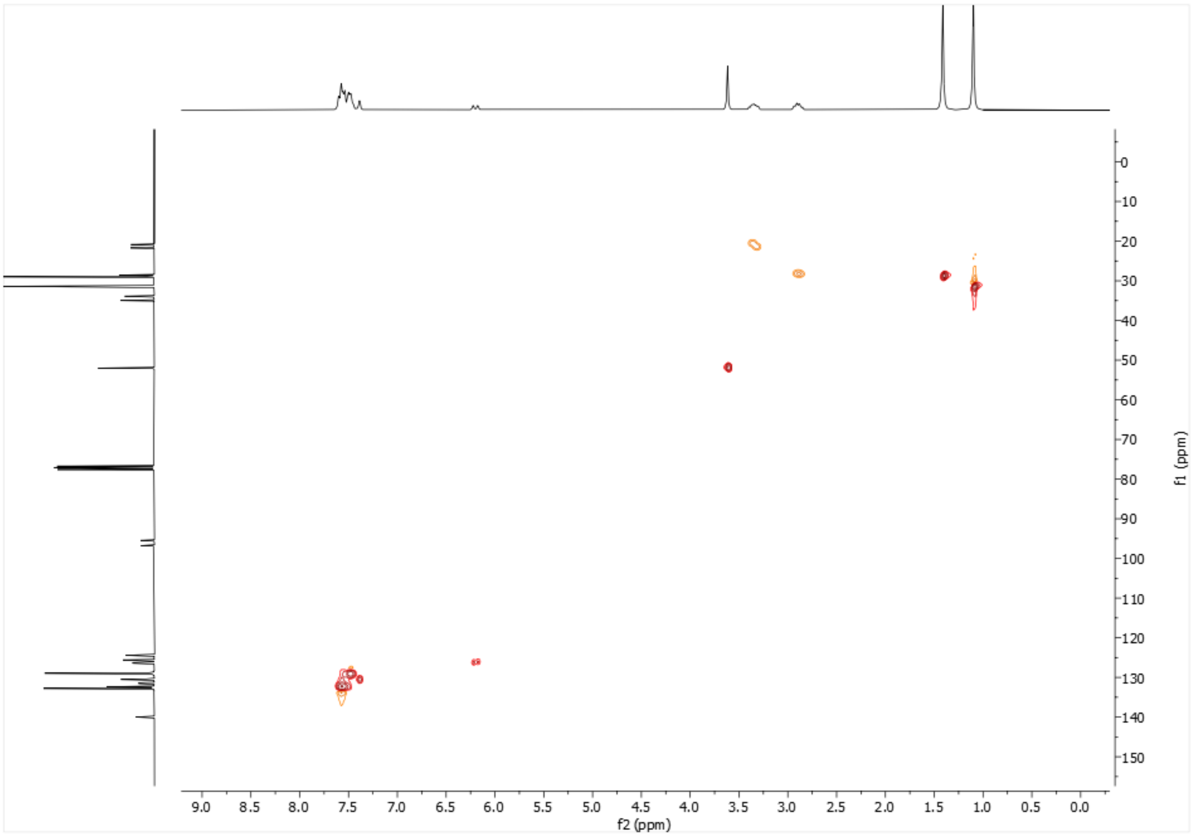

*Figure S20: ^{1}H-^{13}C HSQC NMR spectrum of **2d** recorded from a solution in $CDCl_3$ at 298 K.*

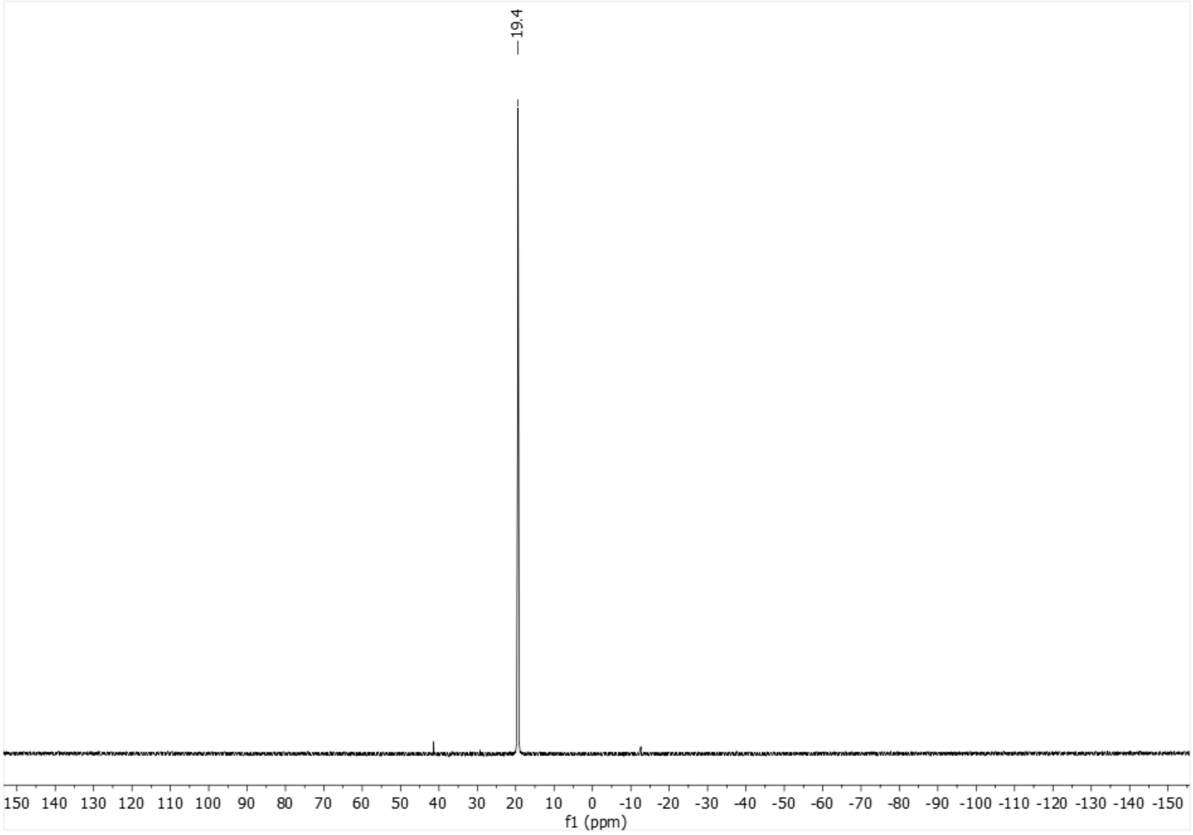

*Figure S21: $^{31}P\{^{1}H\}$ NMR spectrum of **2d** recorded from a solution in $CDCl_3$ at 298 K.*

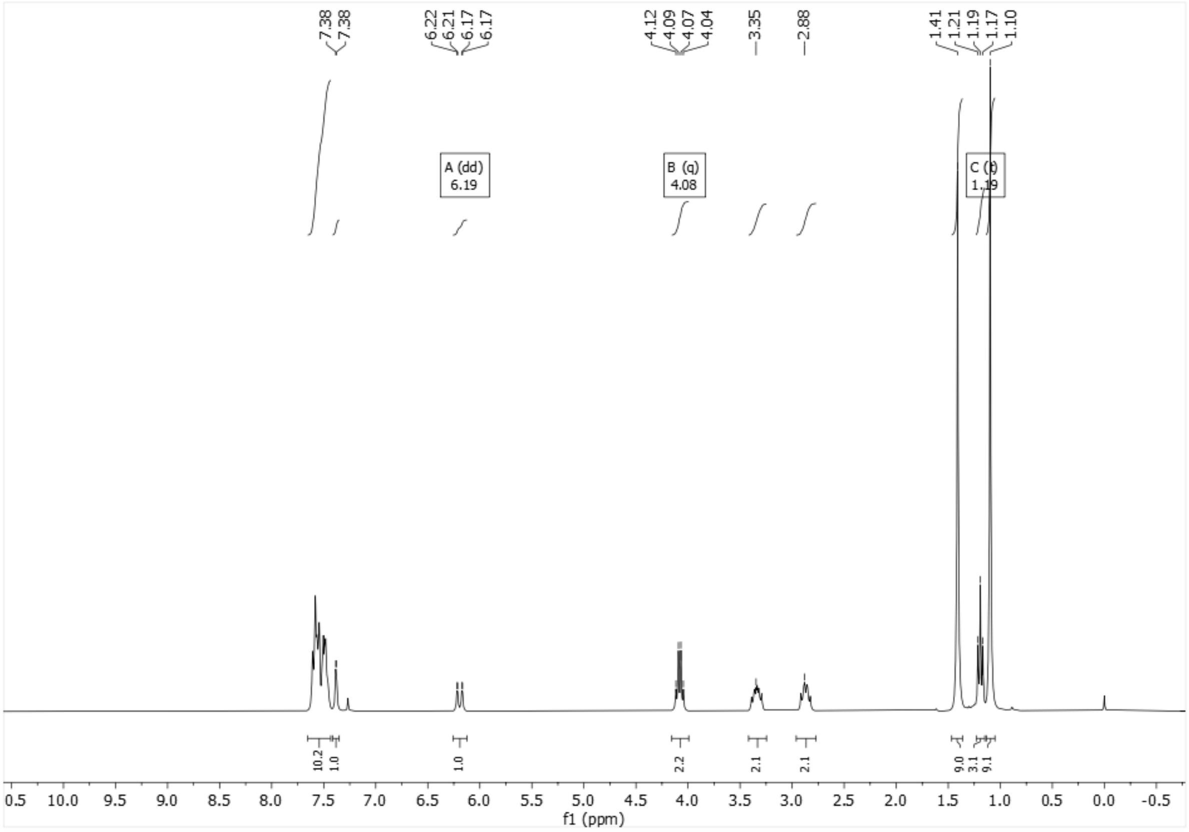

*Figure S22: ^{1}H NMR spectrum of **2e** recorded from a solution in $CDCl_3$ at 298K.*

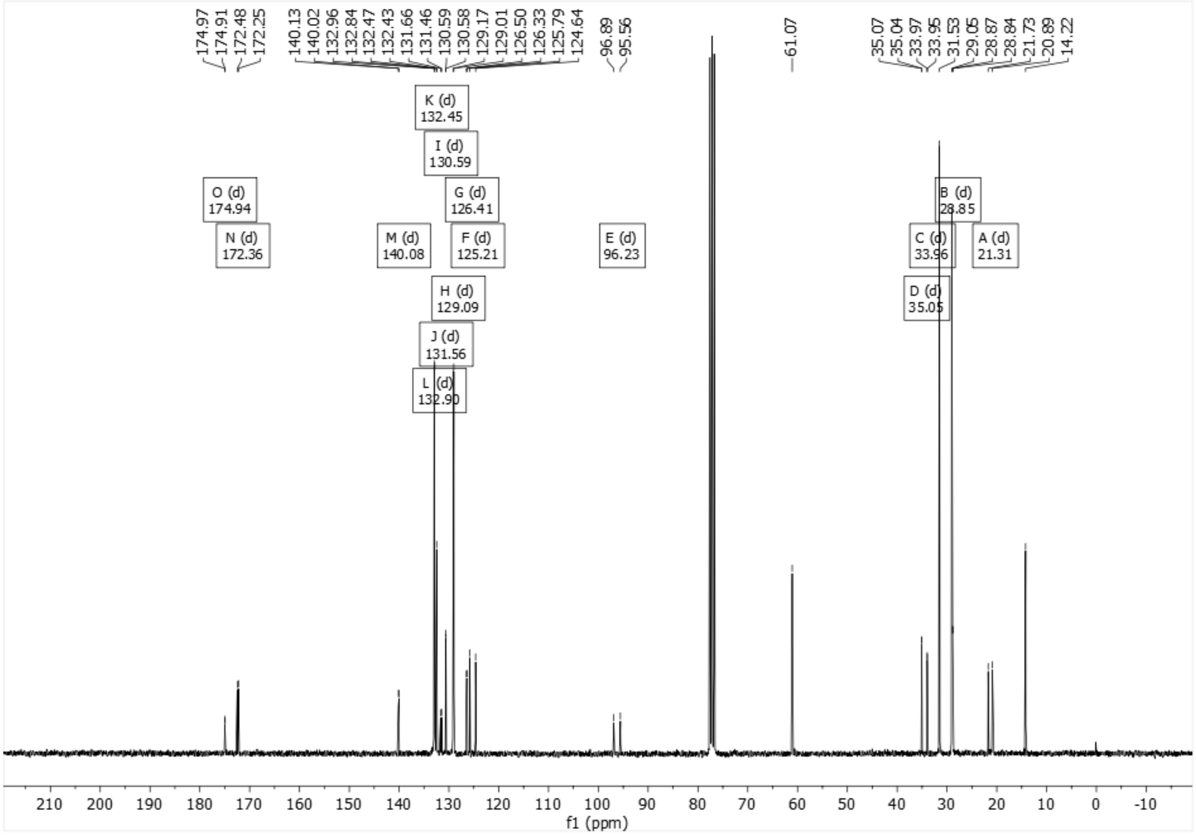

*Figure S23: $^{13}C\{^{1}H\}$ NMR spectrum of **2e** recorded from a solution in $CDCl_3$ at 298 K.*

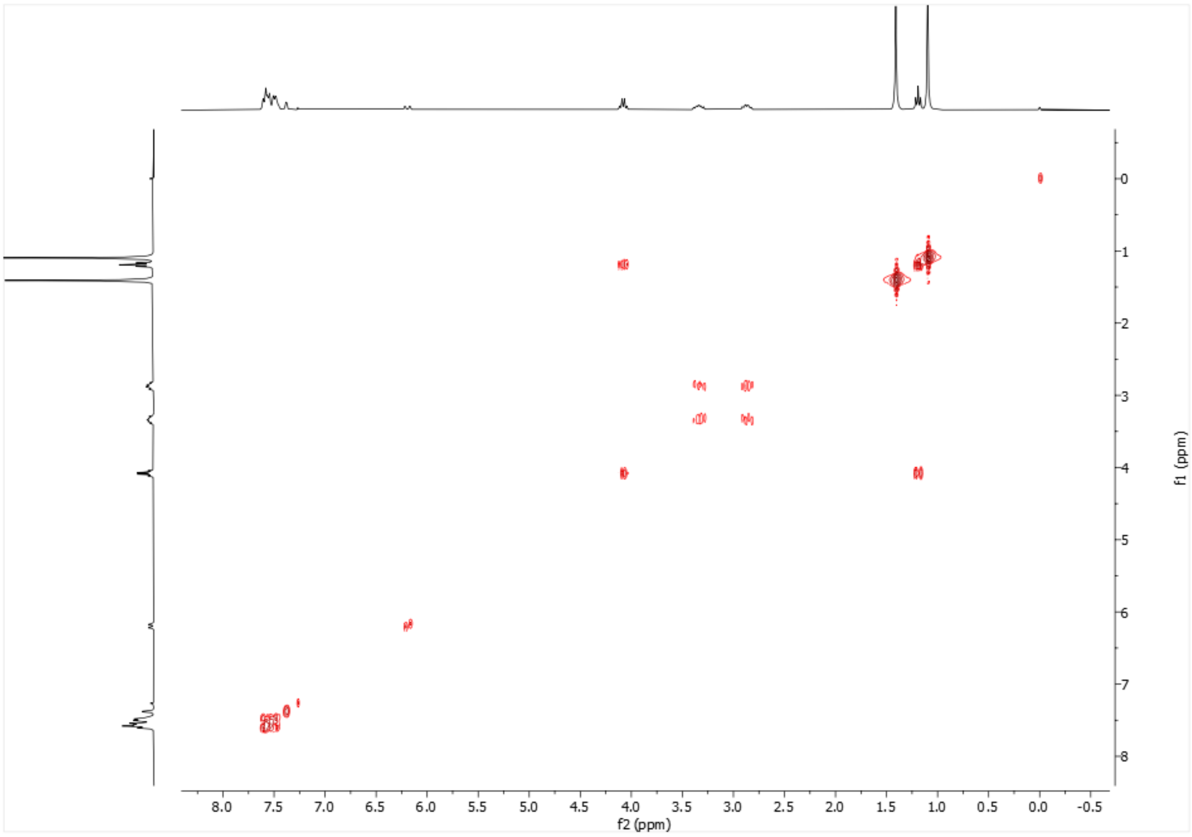

*Figure S24: ^{1}H-^{1}H COSY NMR spectrum of **2e** recorded from a solution in $CDCl_3$ at 298 K.*

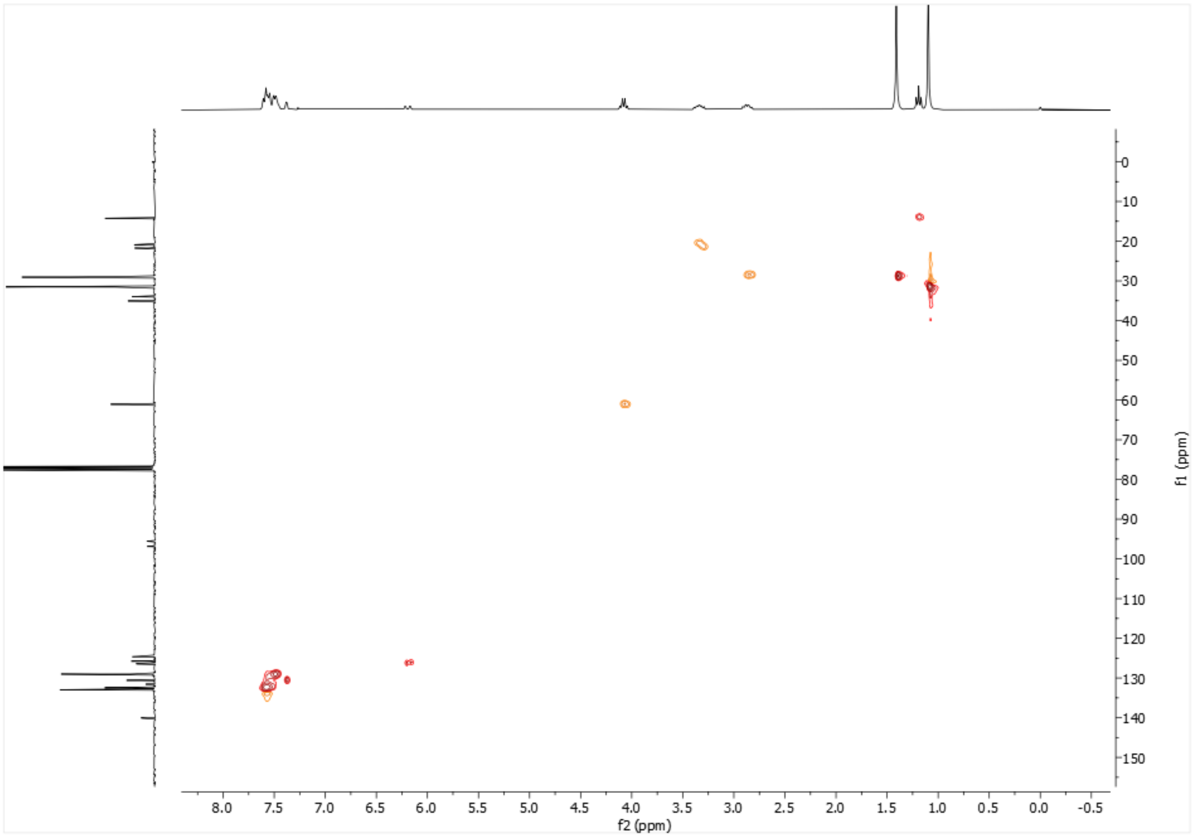

*Figure S25: ^{1}H-^{13}C HSQC NMR spectrum of **2e** recorded from a solution in $CDCl_3$ at 298 K.*

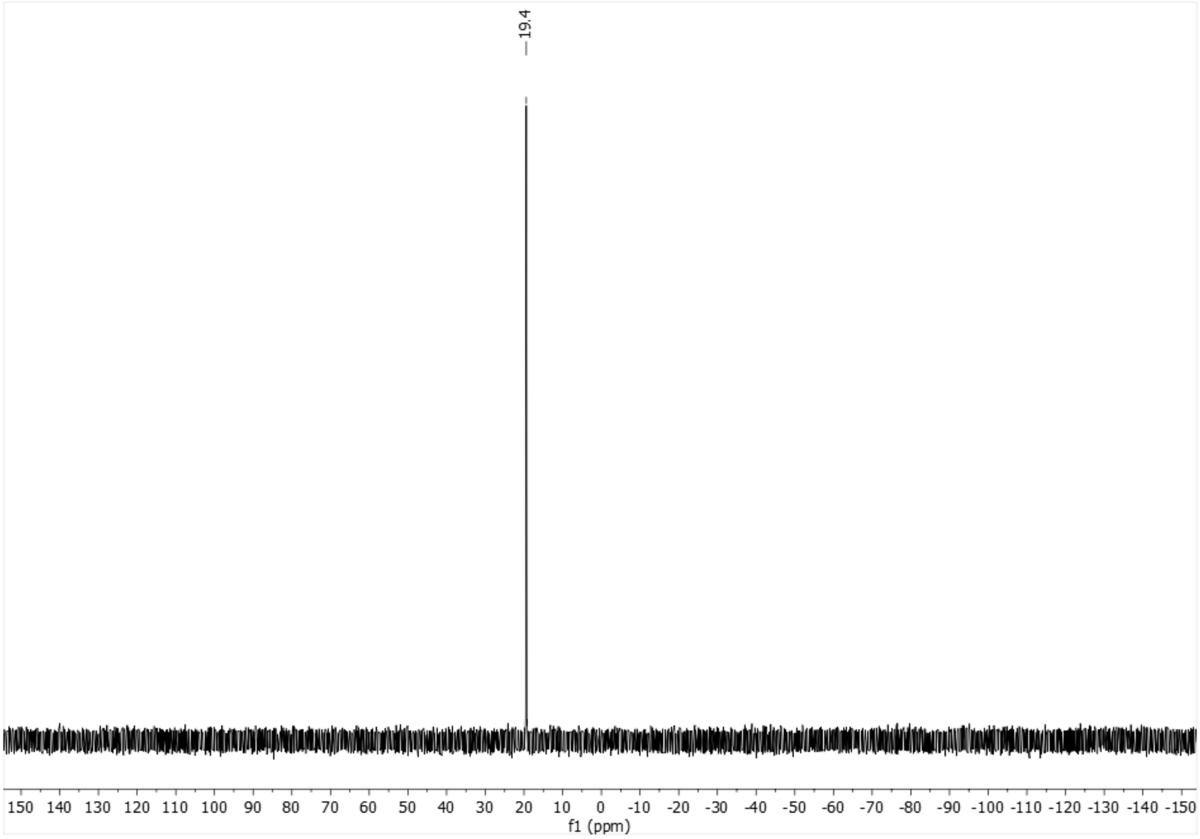

*Figure S26: $^{31}P\{^{1}H\}$ NMR spectrum of **2e** recorded from a solution in $CDCl_3$ at 298 K.*

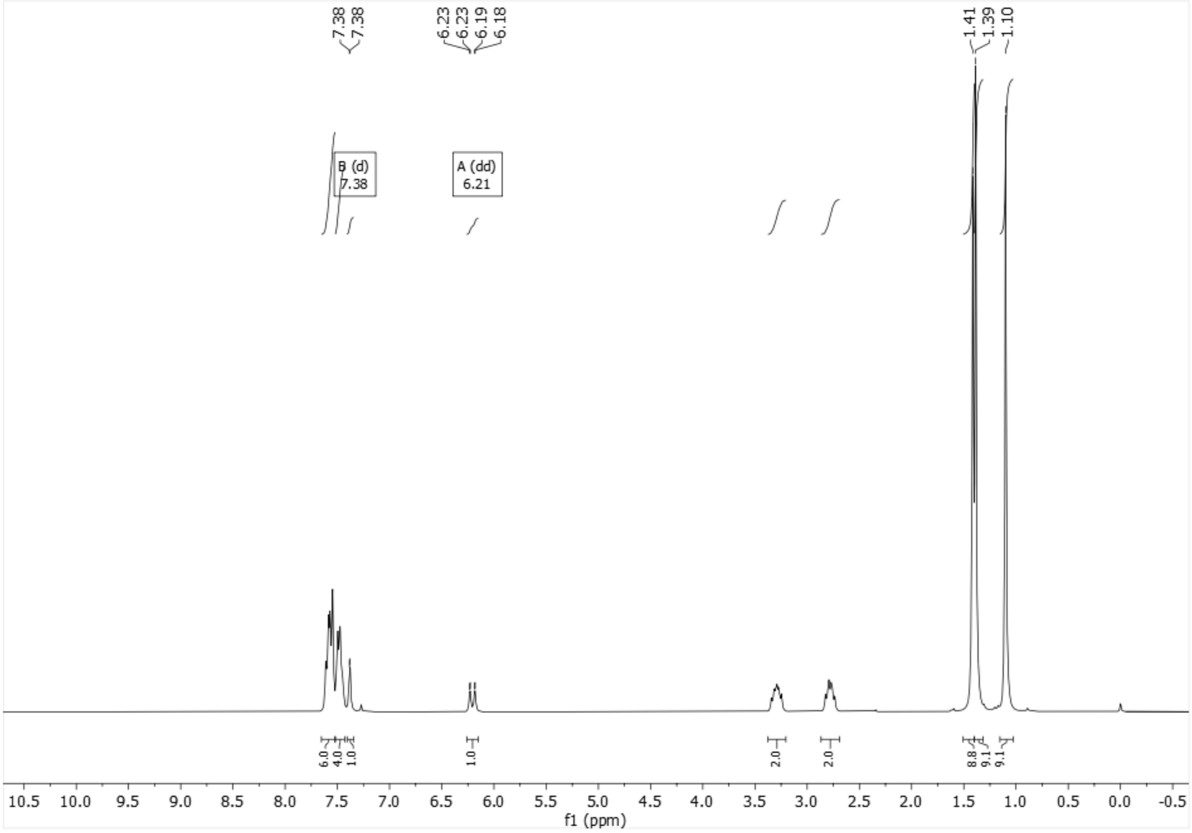

*Figure S27: ^{1}H NMR spectrum of **2f** recorded from a solution in $CDCl_3$ at 298 K.*

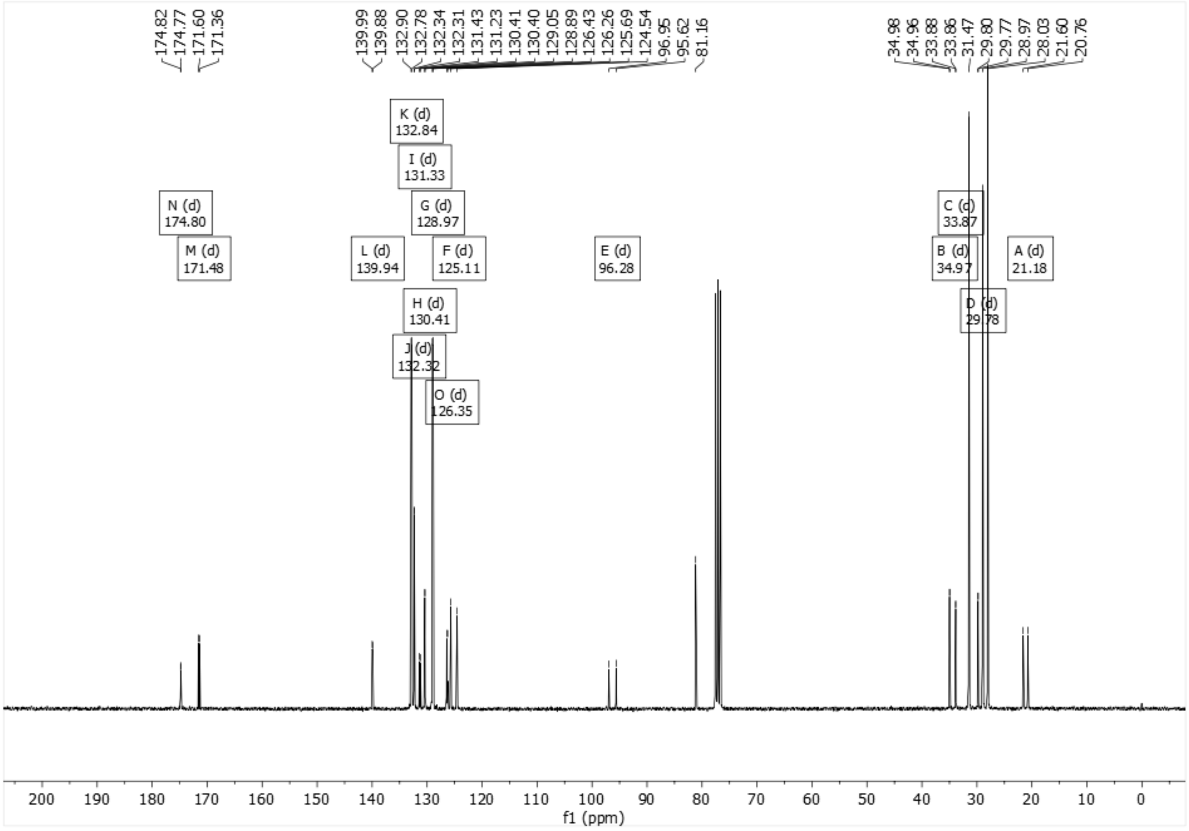

*Figure S28: $^{13}C\{^{1}H\}$ NMR spectrum of **2f** recorded from a solution in $CDCl_3$ at 298 K.*

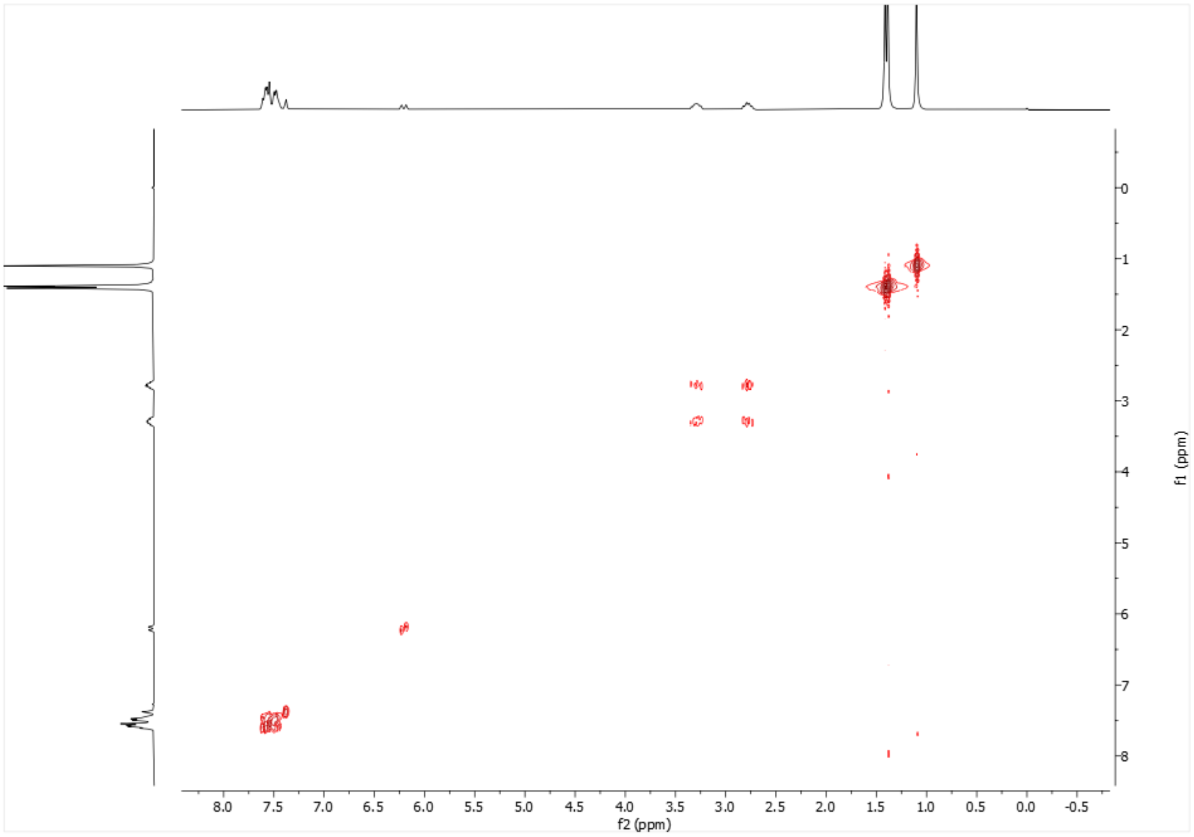

*Figure S29: ^{1}H-^{1}H COSY NMR spectrum of **2f** recorded from a solution in $CDCl_3$ at 298 K.*

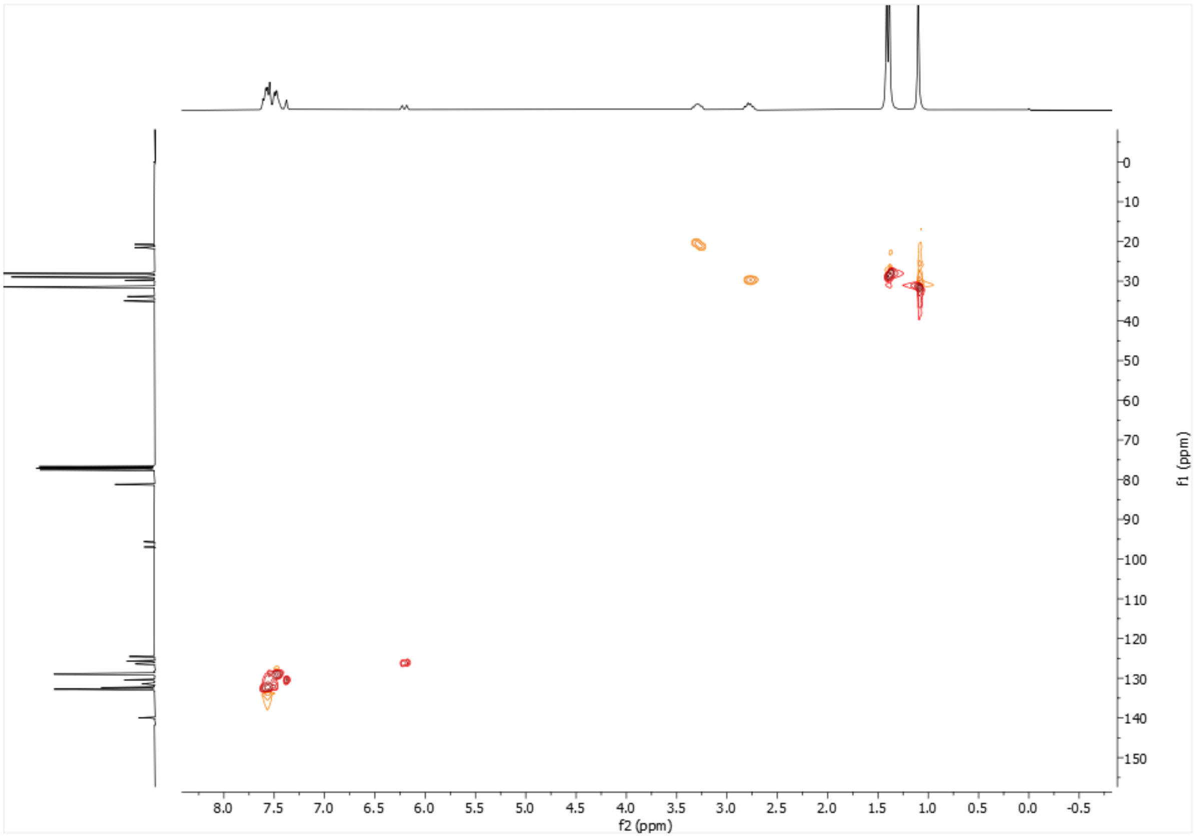

*Figure S30: ^{1}H-^{13}C HSQC NMR spectrum of **2f** recorded from a solution in $CDCl_3$ at 298 K.*

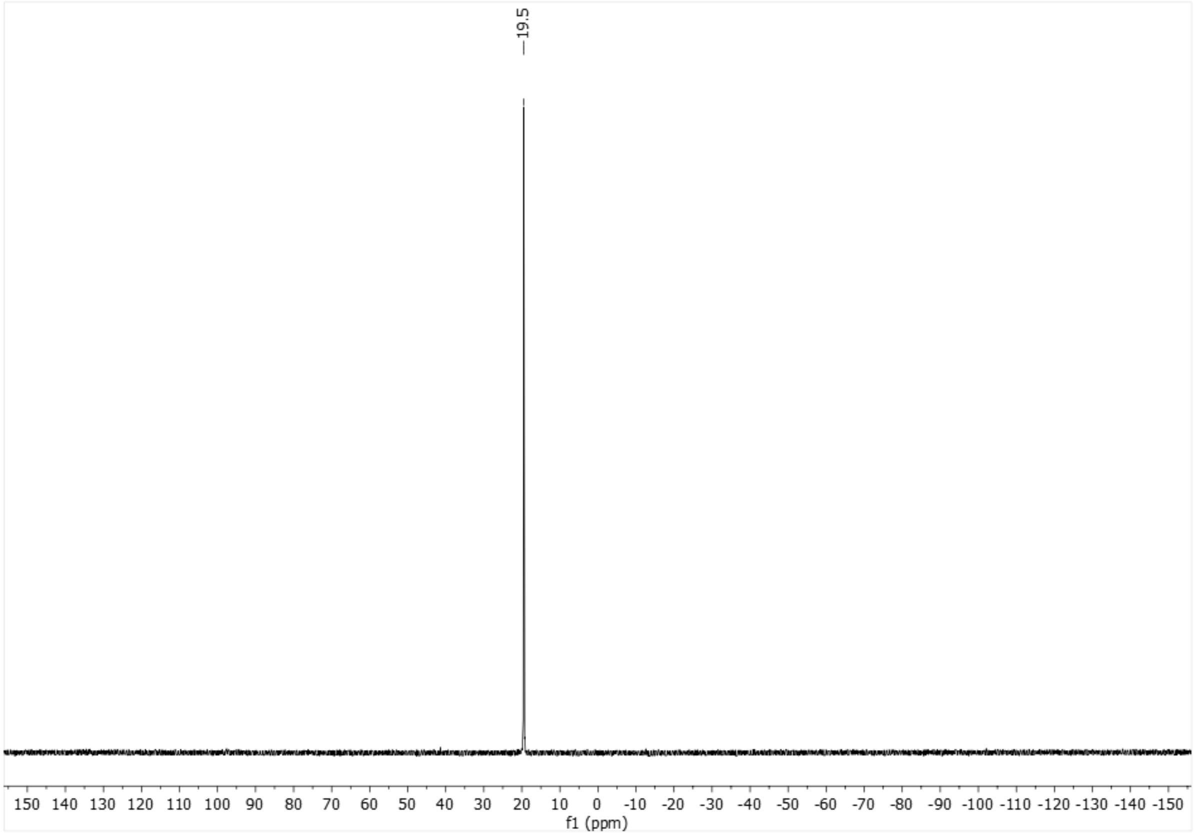

*Figure S31: $^{31}P\{^{1}H\}$ NMR spectrum of **2f** recorded from a solution in $CDCl_3$ at 298 K.*

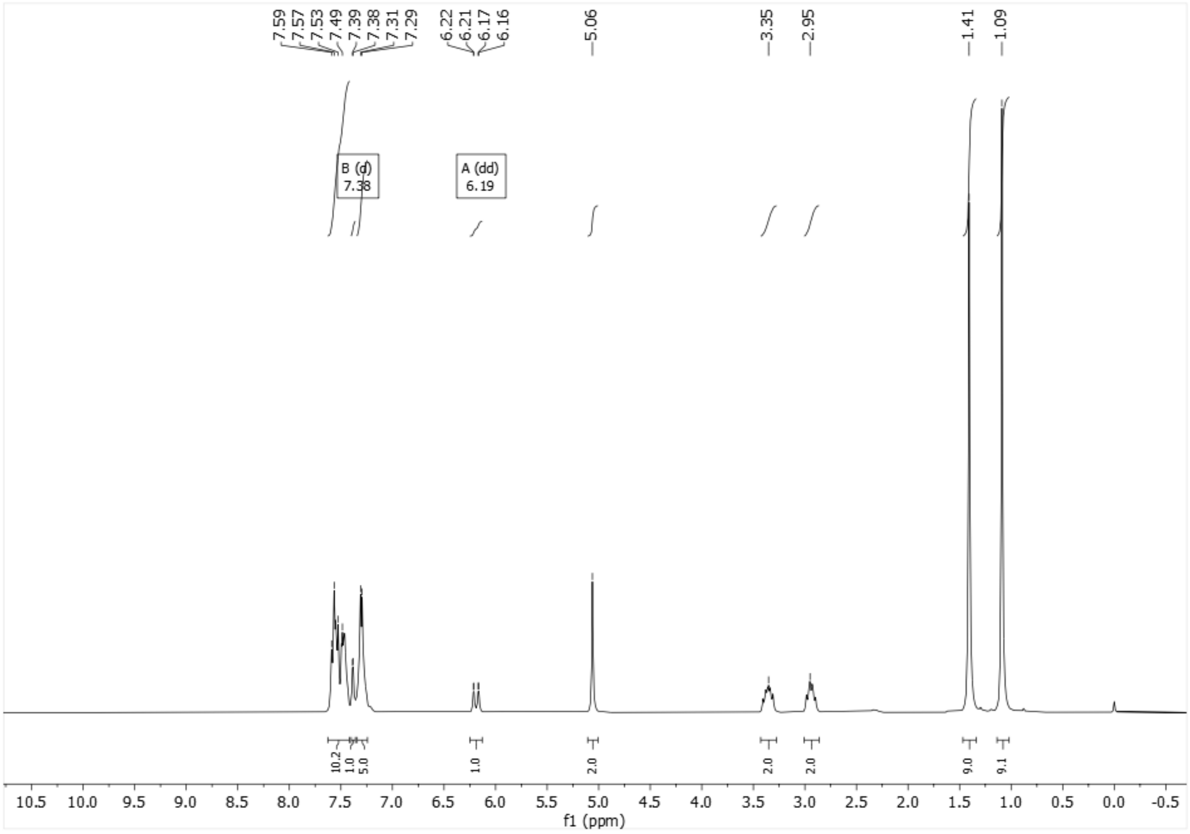

*Figure S32: ¹H NMR spectrum of **2g** recorded from a solution in $CDCl_3$ at 298 K.*

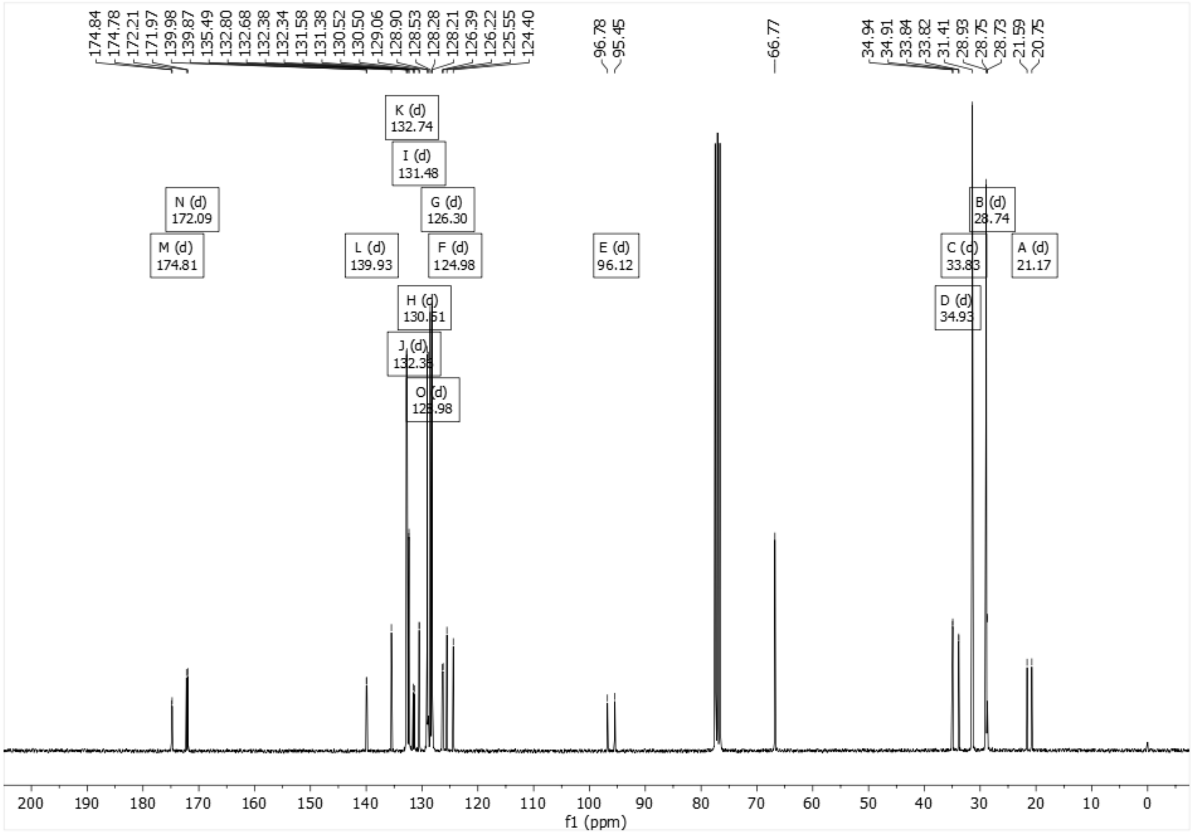

*Figure S33: ¹³C{¹H} NMR spectrum of **2g** recorded from a solution in $CDCl_3$ at 298 K.*

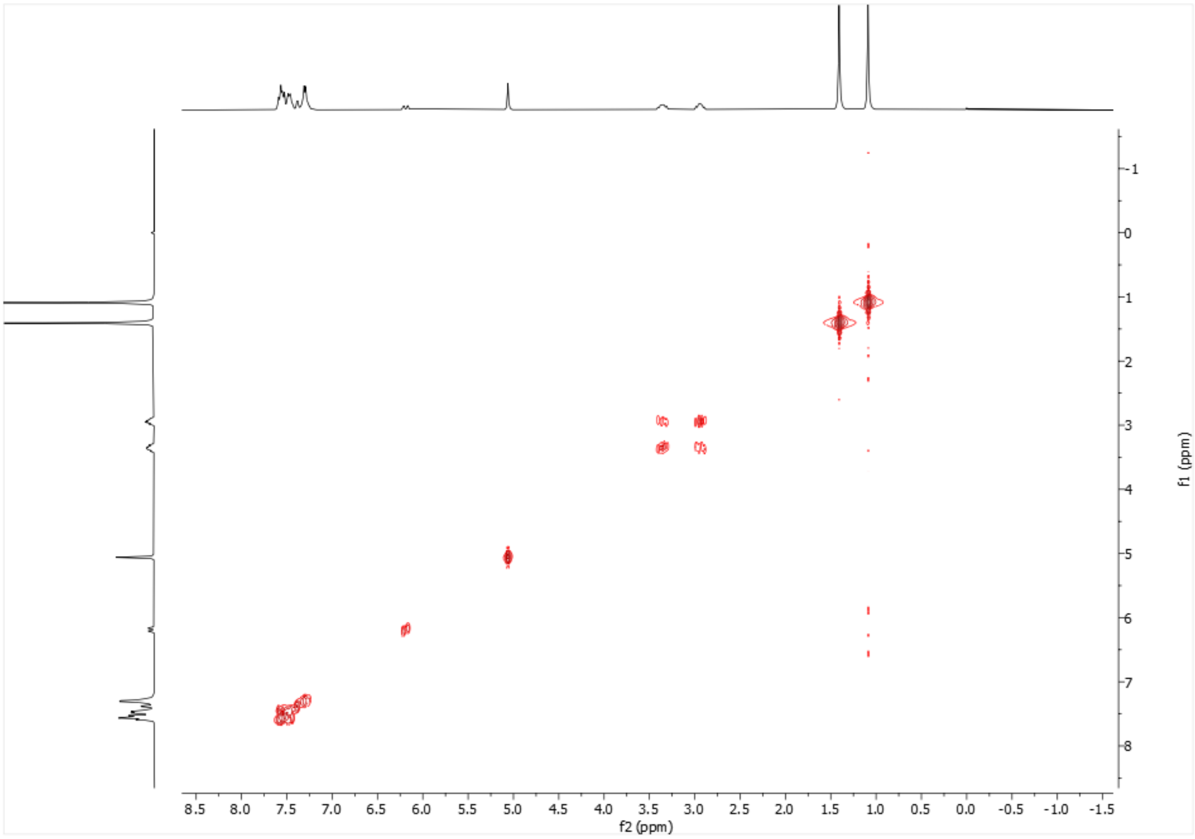

*Figure S34: ^{1}H-^{1}H COSY NMR spectrum of **2g** recorded from a solution in $CDCl_3$ at 298 K.*

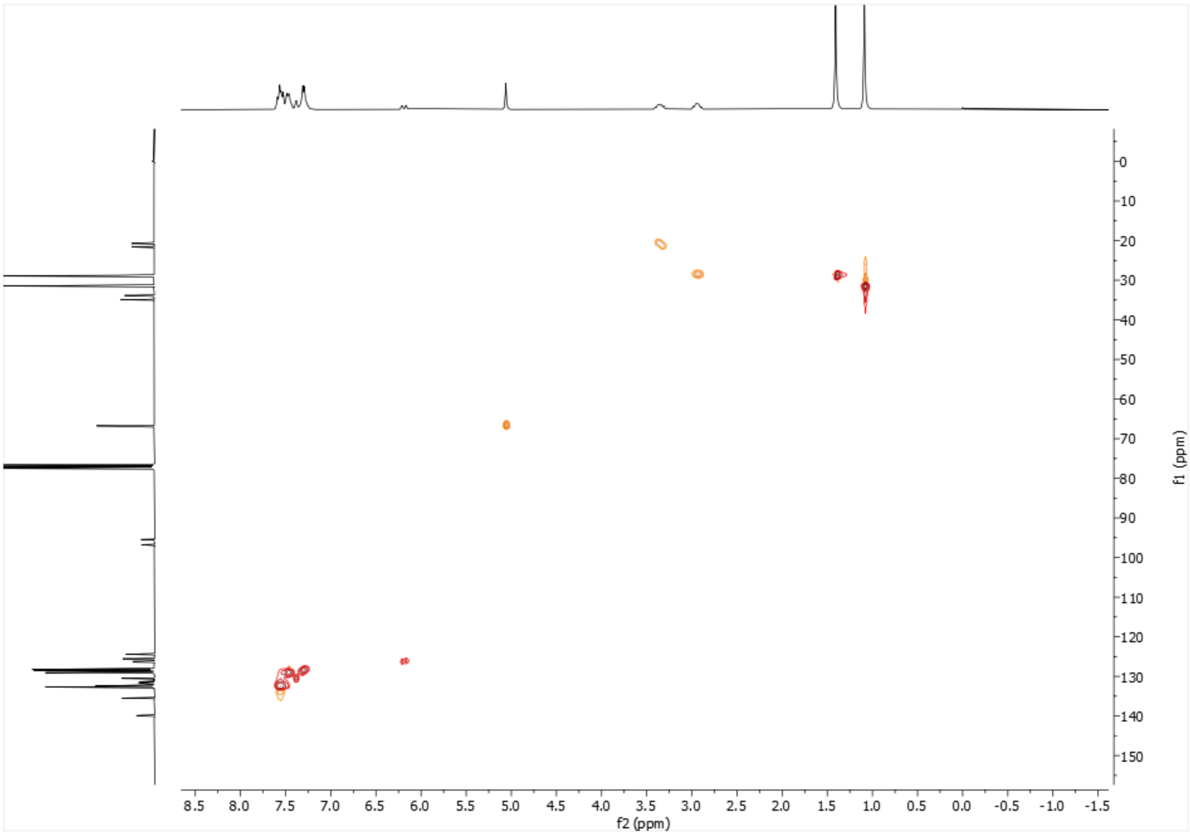

*Figure S35: ^{1}H-^{13}C HSQC NMR spectrum of **2g** recorded from a solution in $CDCl_3$ at 298 K.*

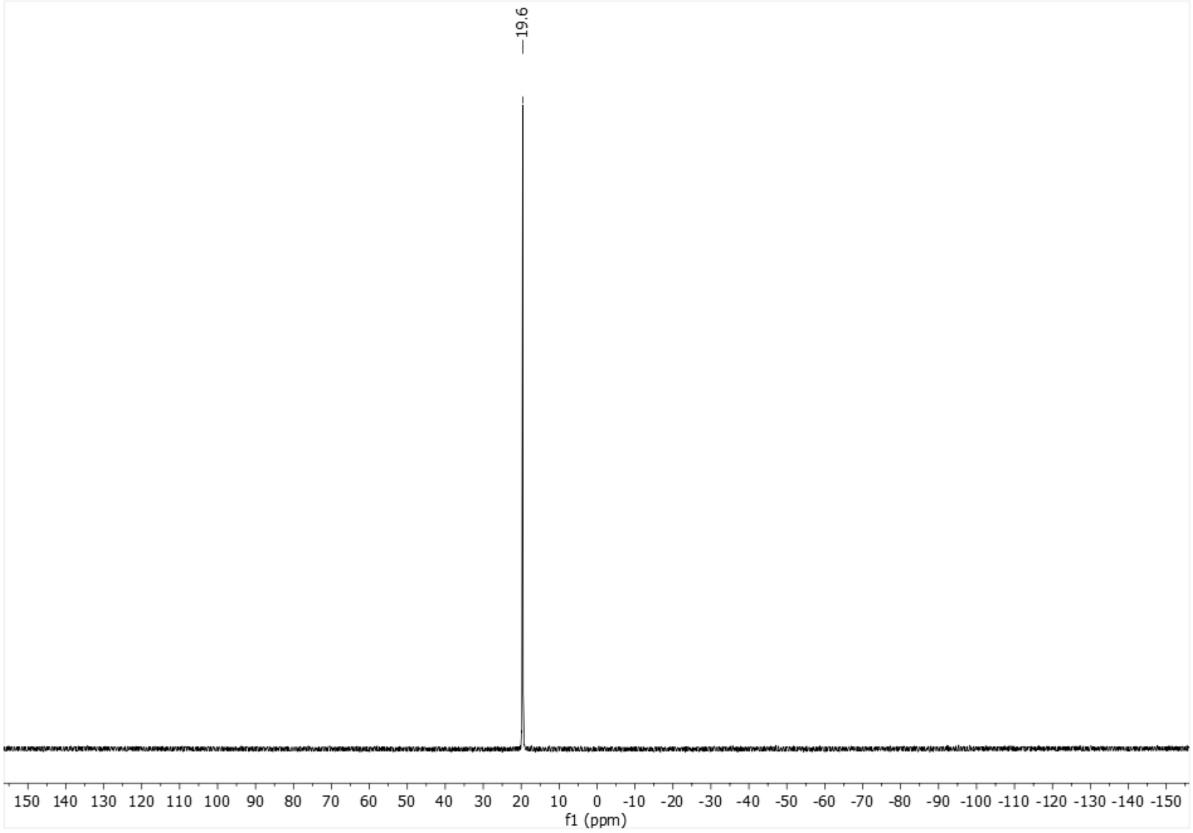

*Figure S36: $^{31}P\{^{1}H\}$ NMR spectrum of **2g** recorded from a solution in $CDCl_3$ at 298 K.*

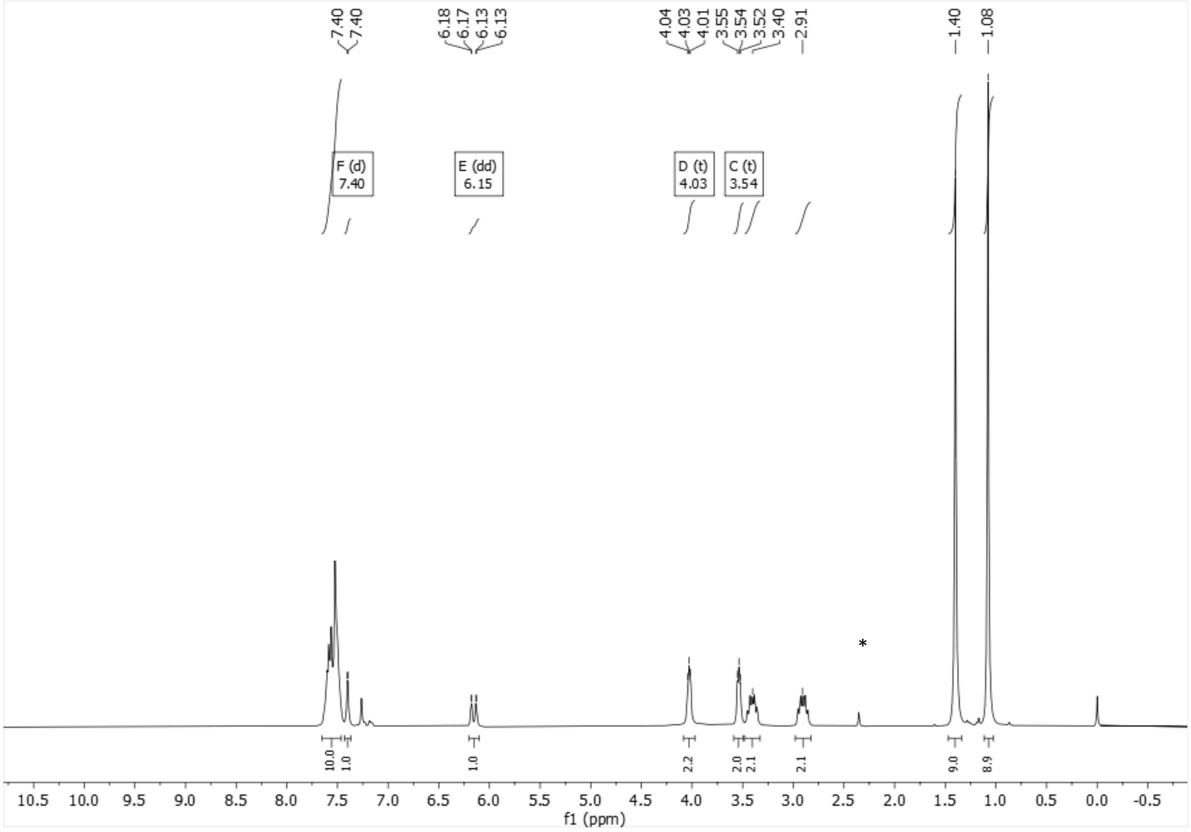

*Figure S37: ^{1}H NMR spectrum of **2h** recorded from a solution in $CDCl_3$ at 298 K. Trace impurities are marked with an asterisk.*

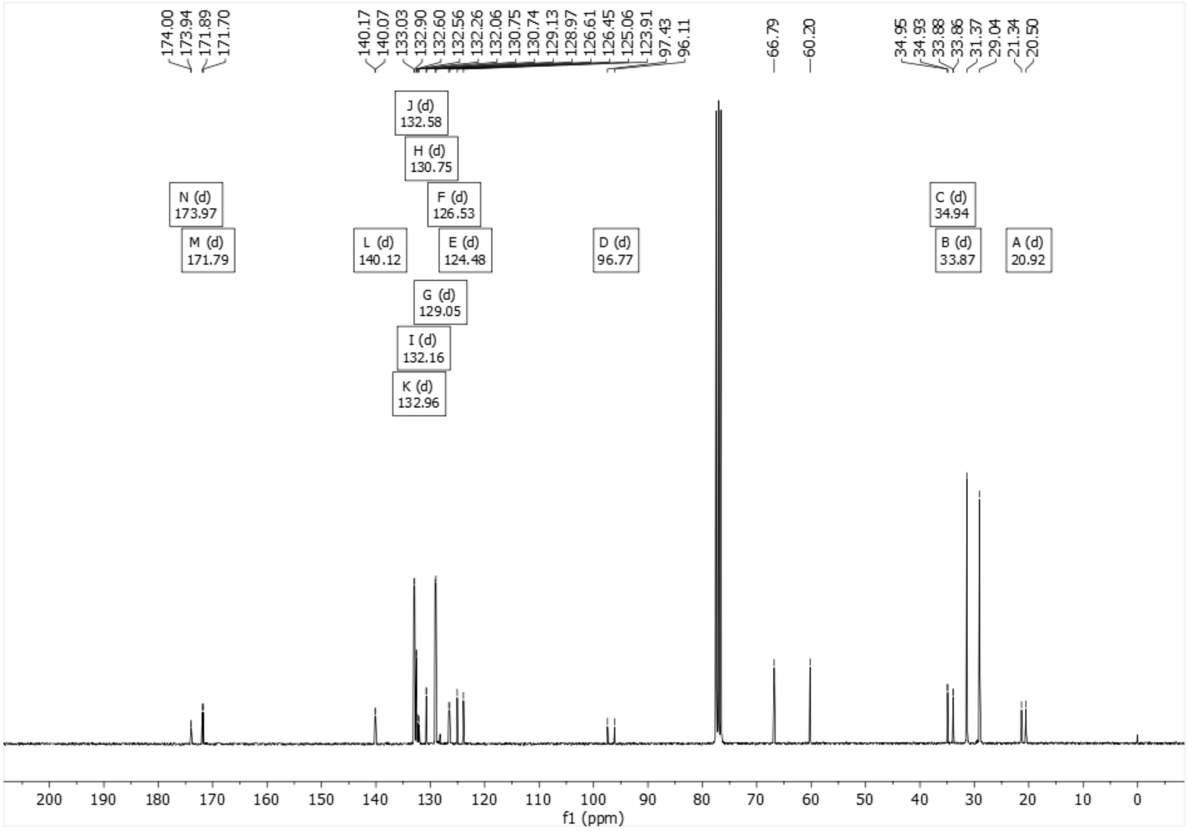

*Figure S38: $^{13}C\{^1H\}$ NMR spectrum of **2h** recorded from a solution in $CDCl_3$ at 298 K.*

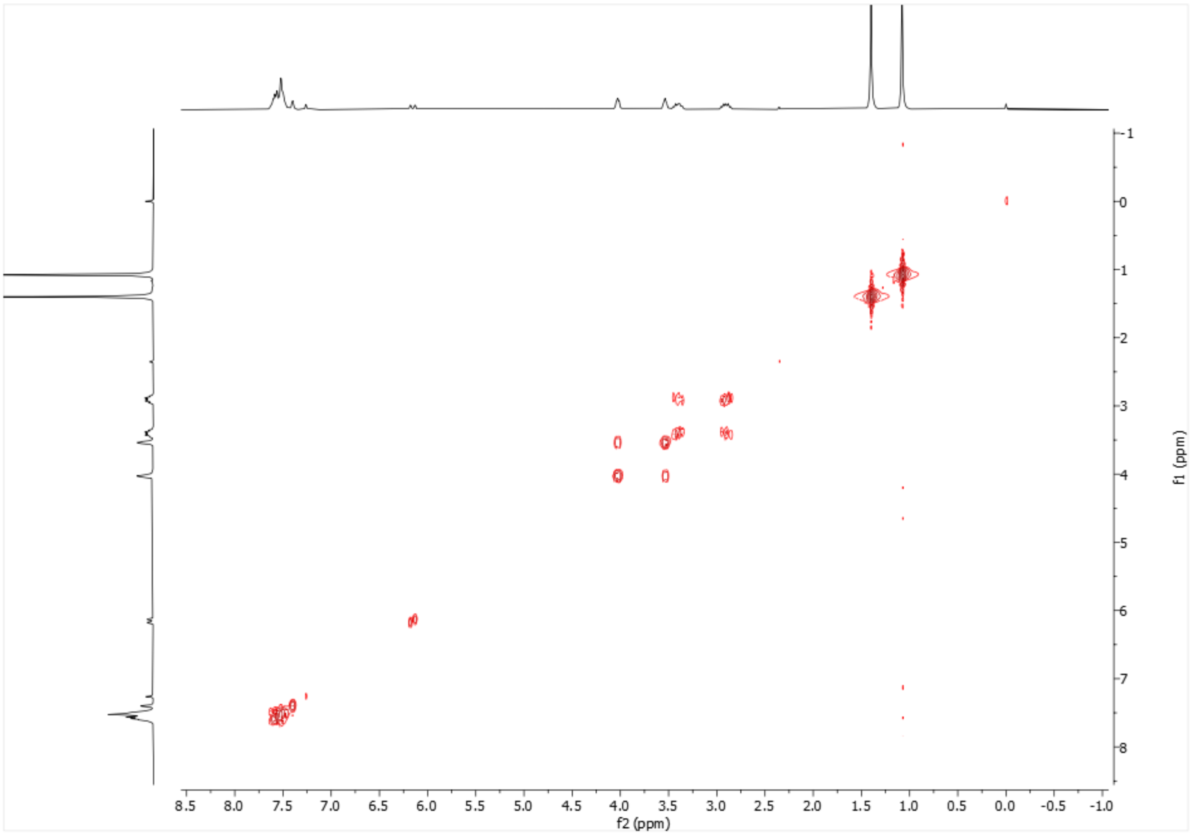

*Figure S39: ^1H-^1H COSY NMR spectrum of **2h** recorded from a solution in $CDCl_3$ at 298 K.*

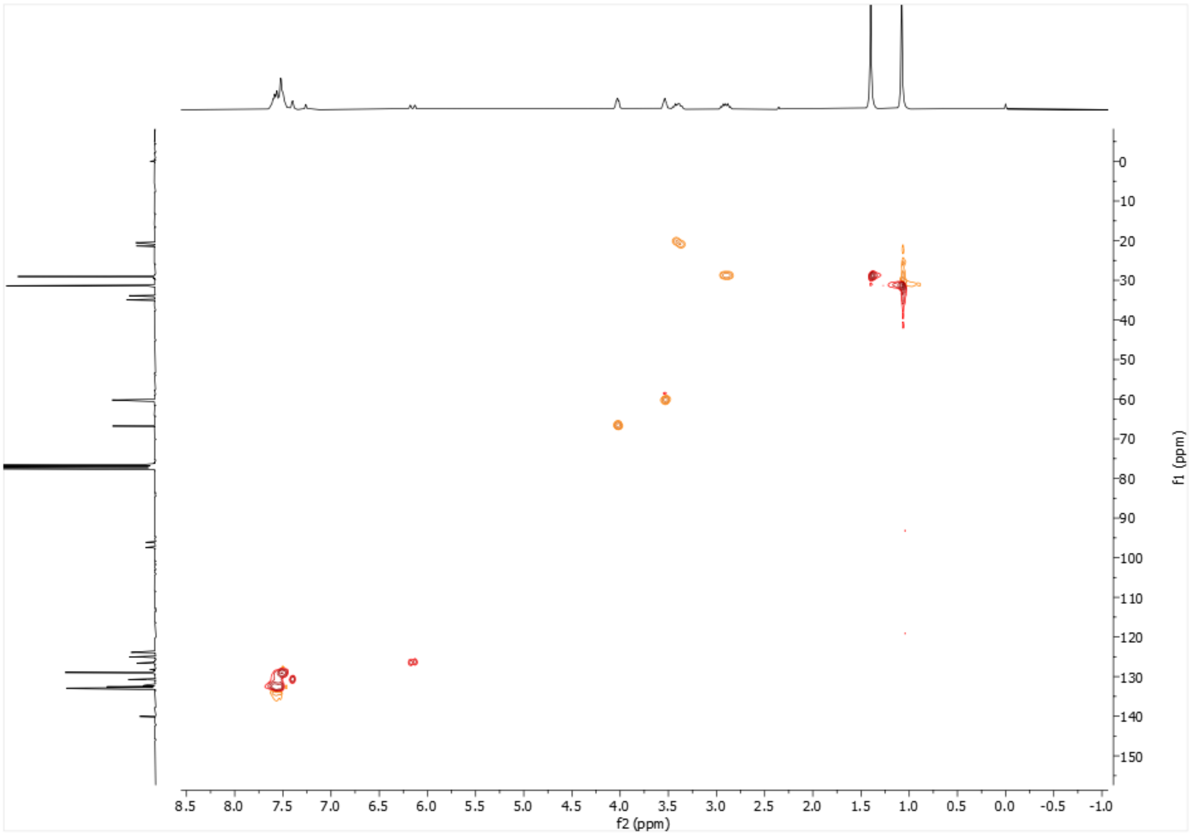

*Figure S40: ^{1}H-^{13}C HSQC NMR spectrum of **2h** recorded from a solution in $CDCl_3$ at 298 K.*

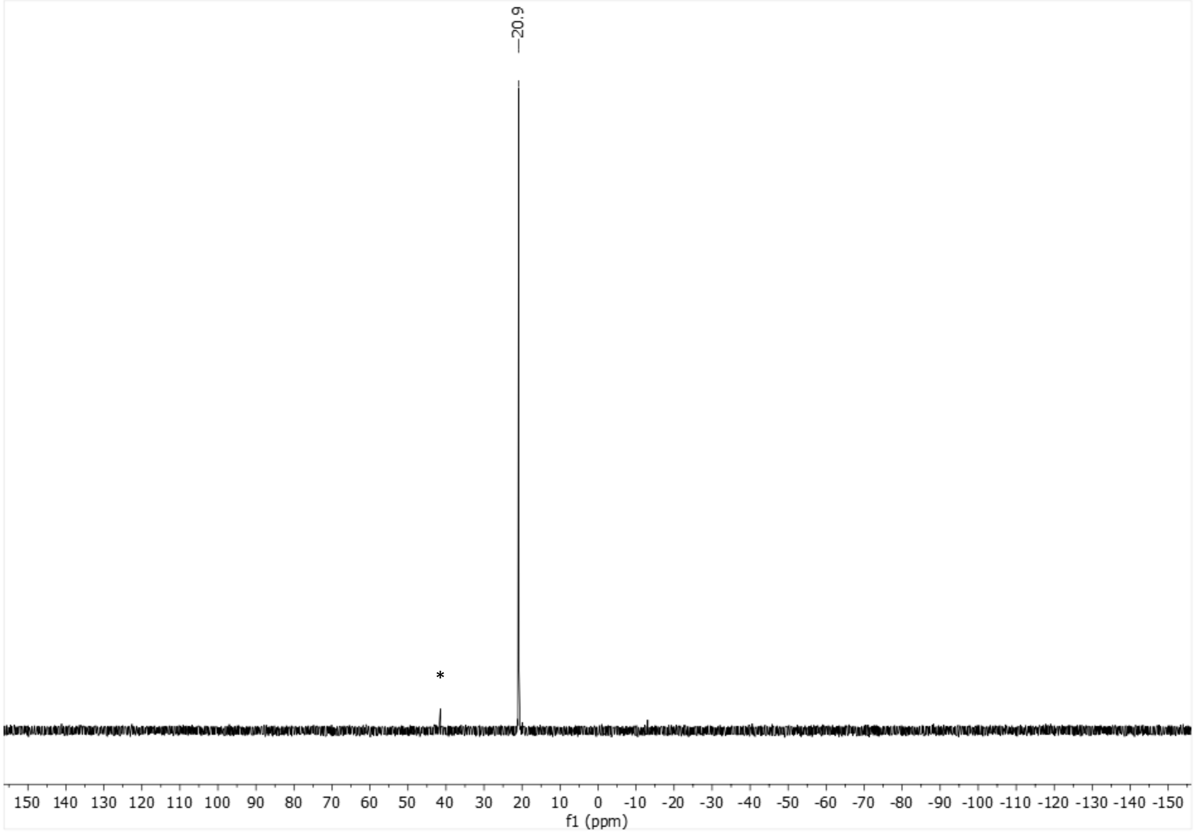

*Figure S41: $^{31}P\{^{1}H\}$ NMR spectrum of **2h** recorded from a solution in $CDCl_3$ at 298 K. Trace impurities are marked with an asterisk.*

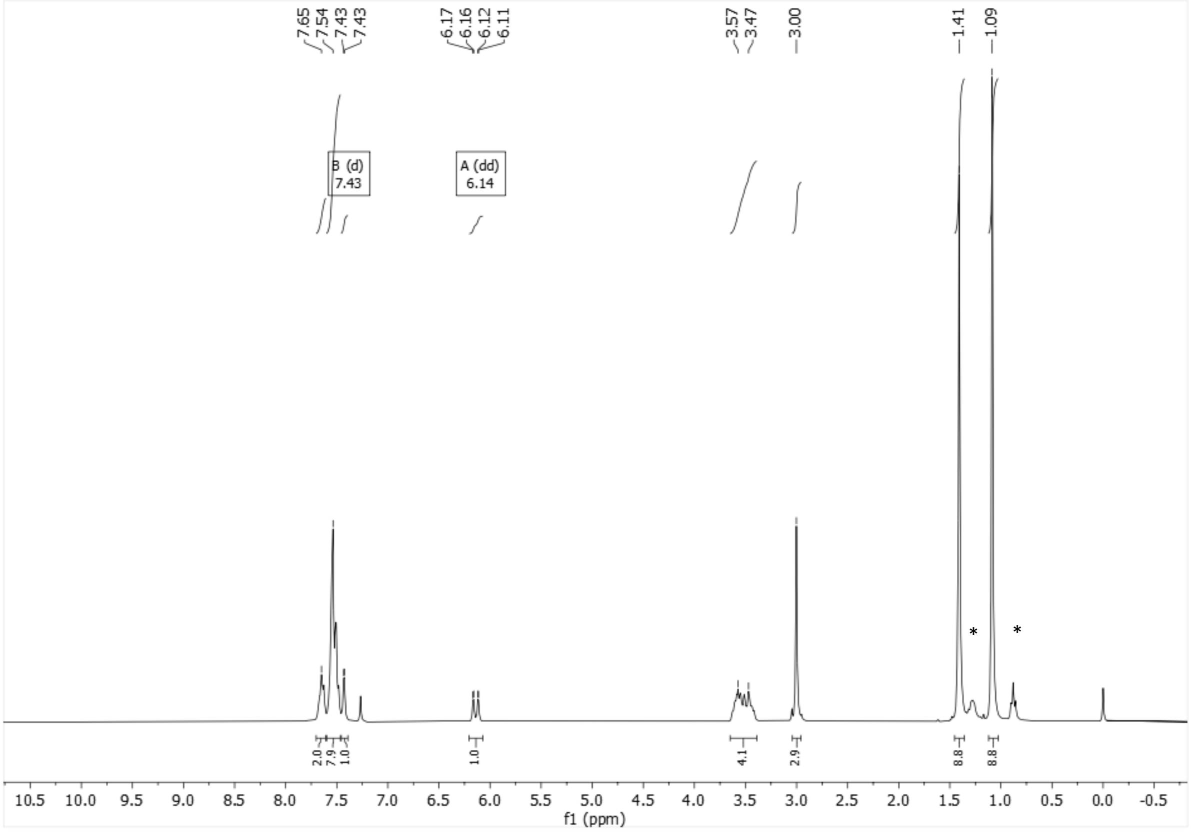

*Figure S42: ^{1}H NMR spectrum of **2i** recorded from a solution in $CDCl_3$ at 298 K. Trace impurities are marked with an asterisk.*

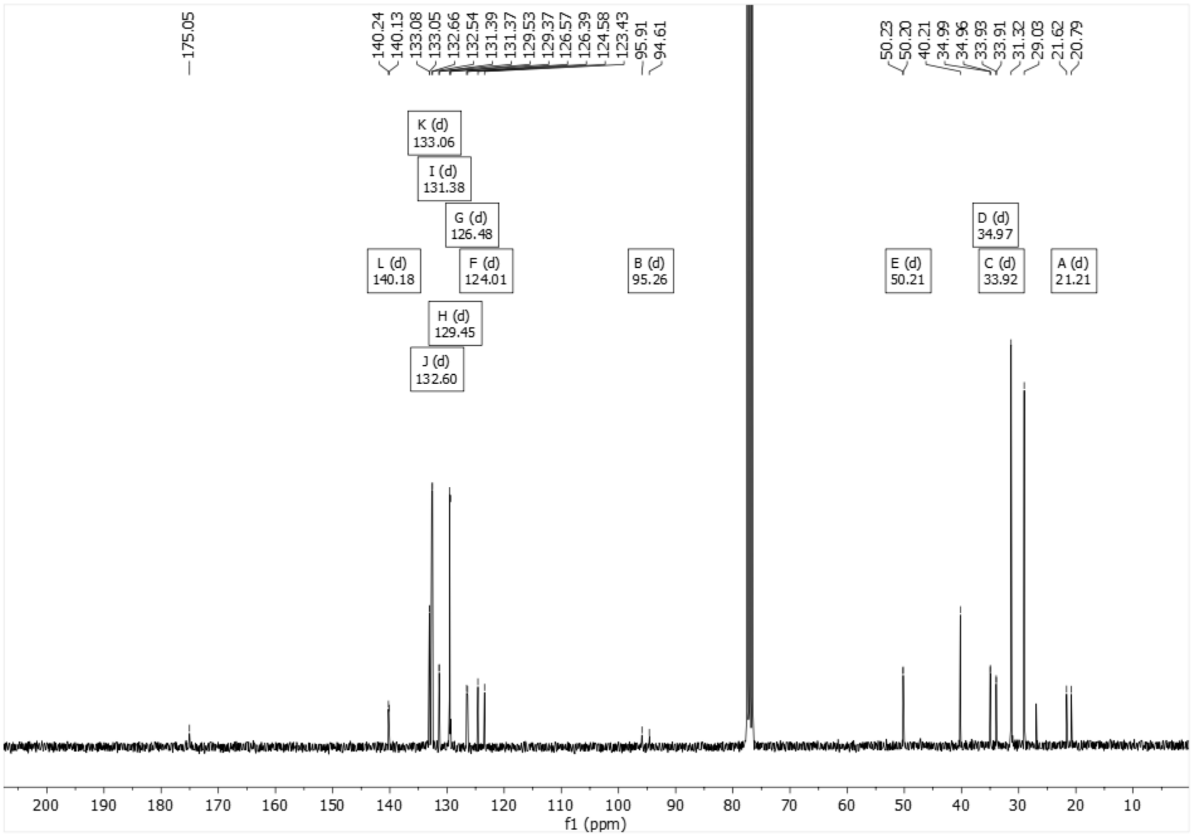

*Figure S43: $^{13}C\{^{1}H\}$ NMR spectrum of **2i** recorded from a solution in $CDCl_3$ at 298 K.*

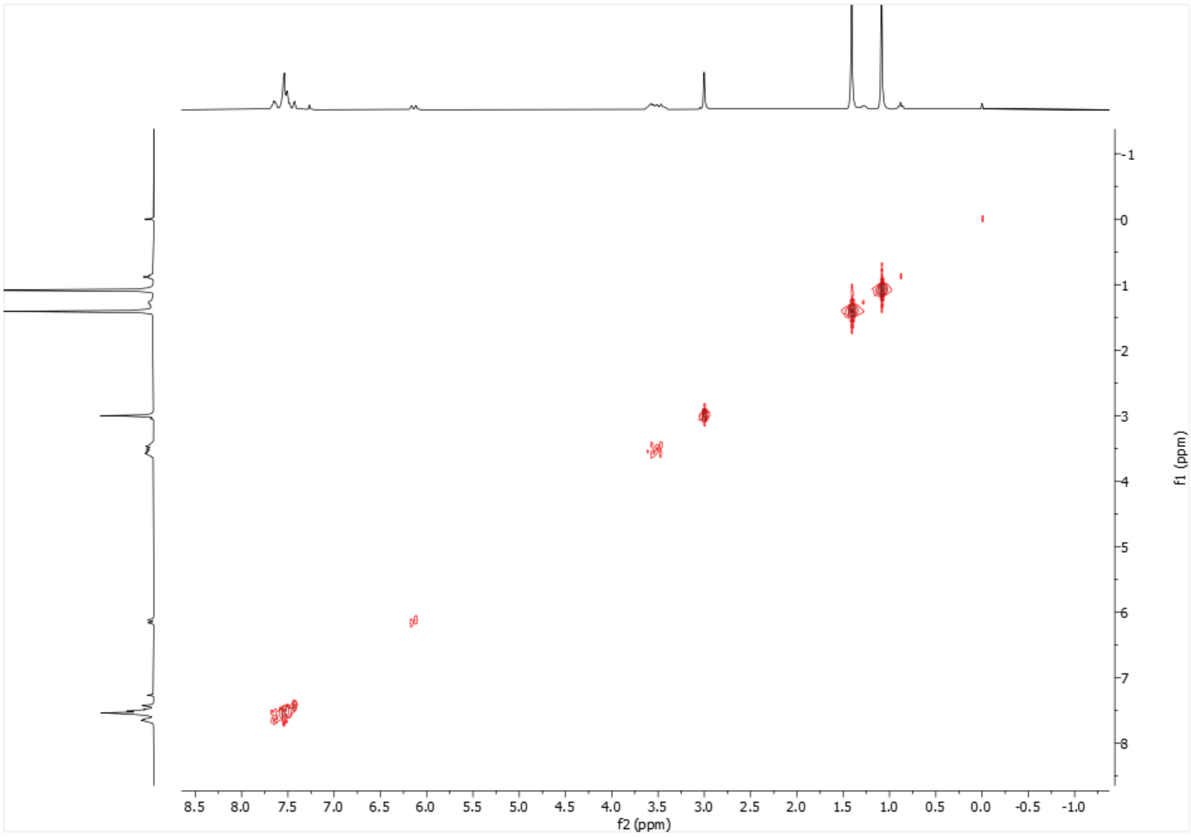

*Figure S44: ^{1}H-^{1}H COSY NMR spectrum of **2i** recorded from a solution in $CDCl_3$ at 298 K.*

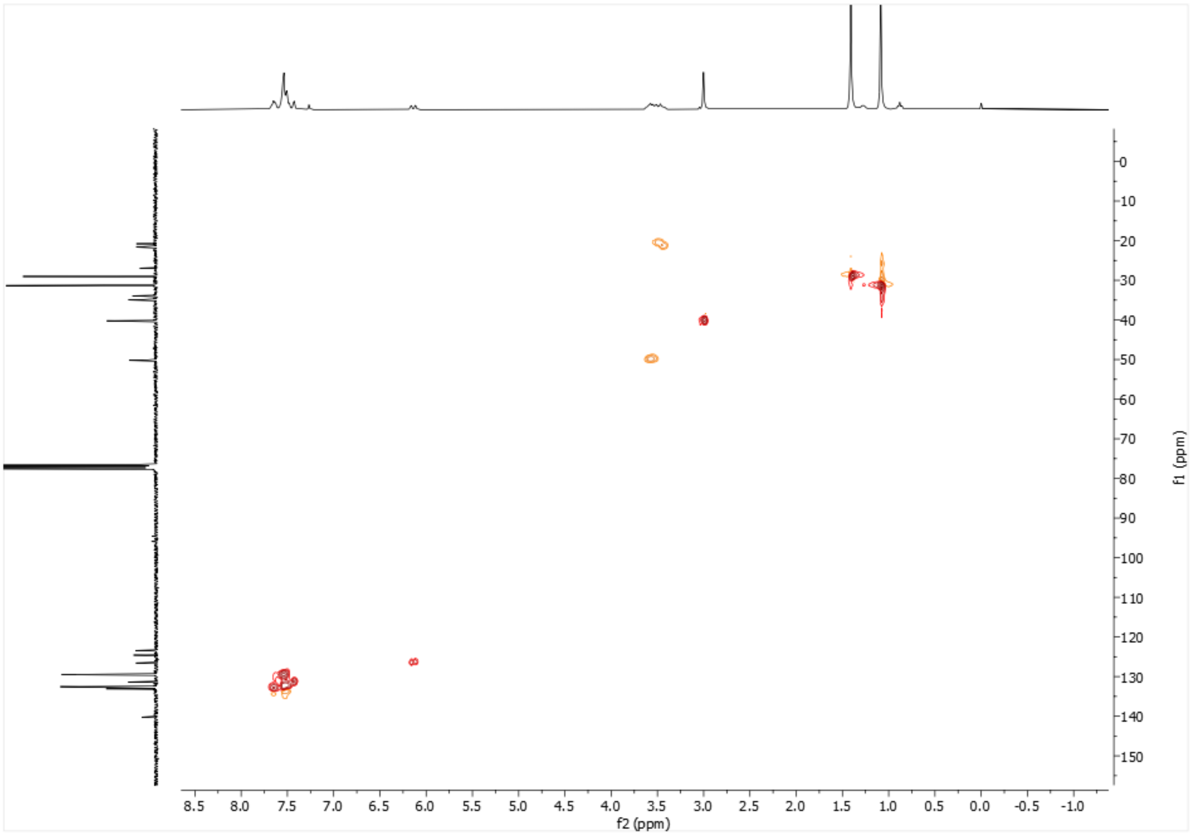

*Figure S45: ^{1}H-^{13}C HSQC NMR spectrum of **2i** recorded from a solution in $CDCl_3$ at 298 K.*

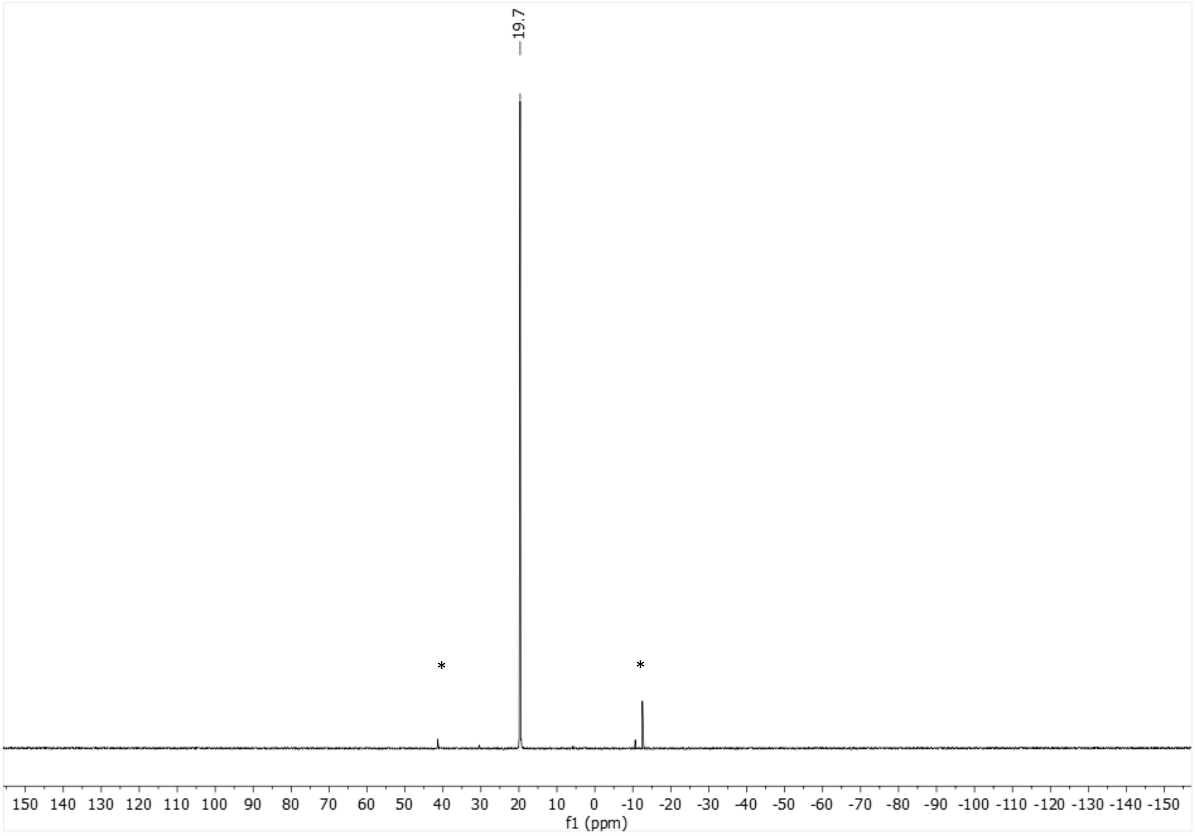

*Figure S46: $^{31}P\{^{1}H\}$ NMR spectrum of **2i** recorded from a solution in $CDCl_3$ at 298 K. Trace impurities are marked with an asterisk.*

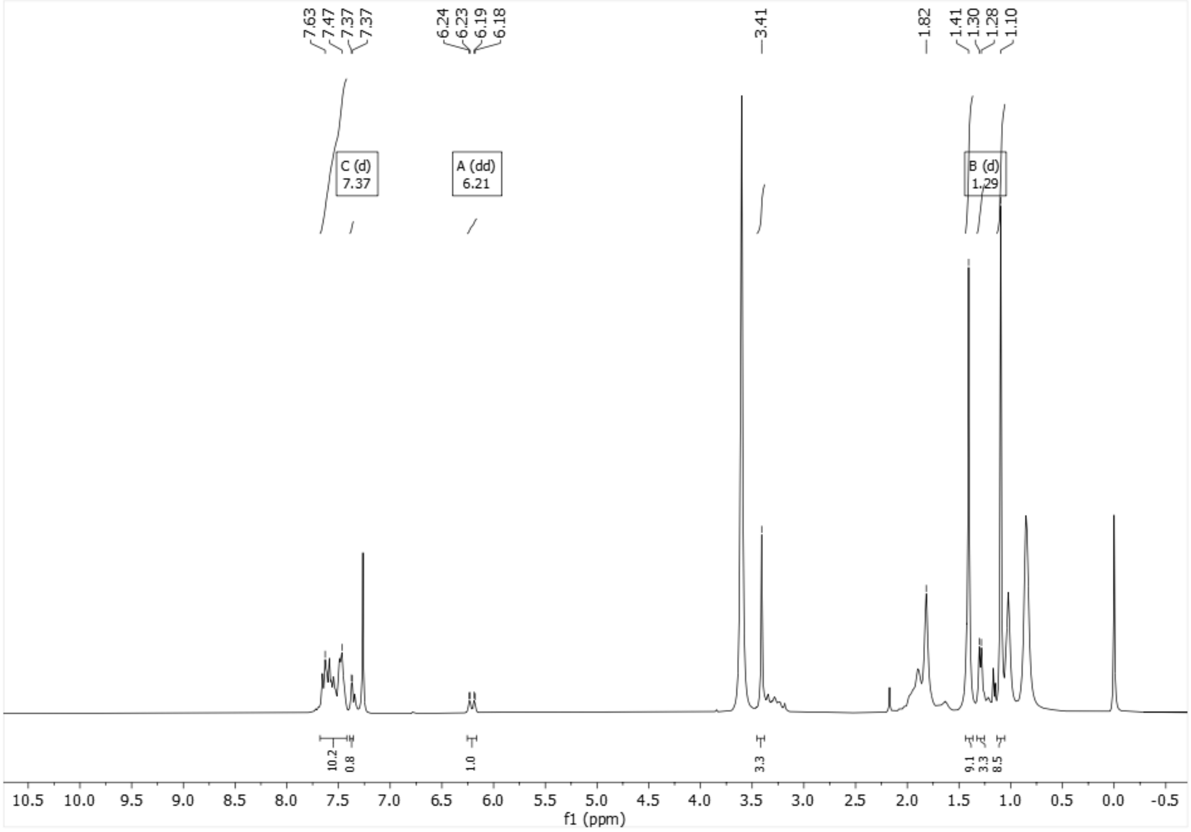

*Figure S47: ^{1}H NMR spectrum of the attempted zwitterion formation from **1** and methyl methacrylate recorded from a solution in $CDCl_3$ at 298 K. Marked peaks indicate the presence of a zwitterionic species.*

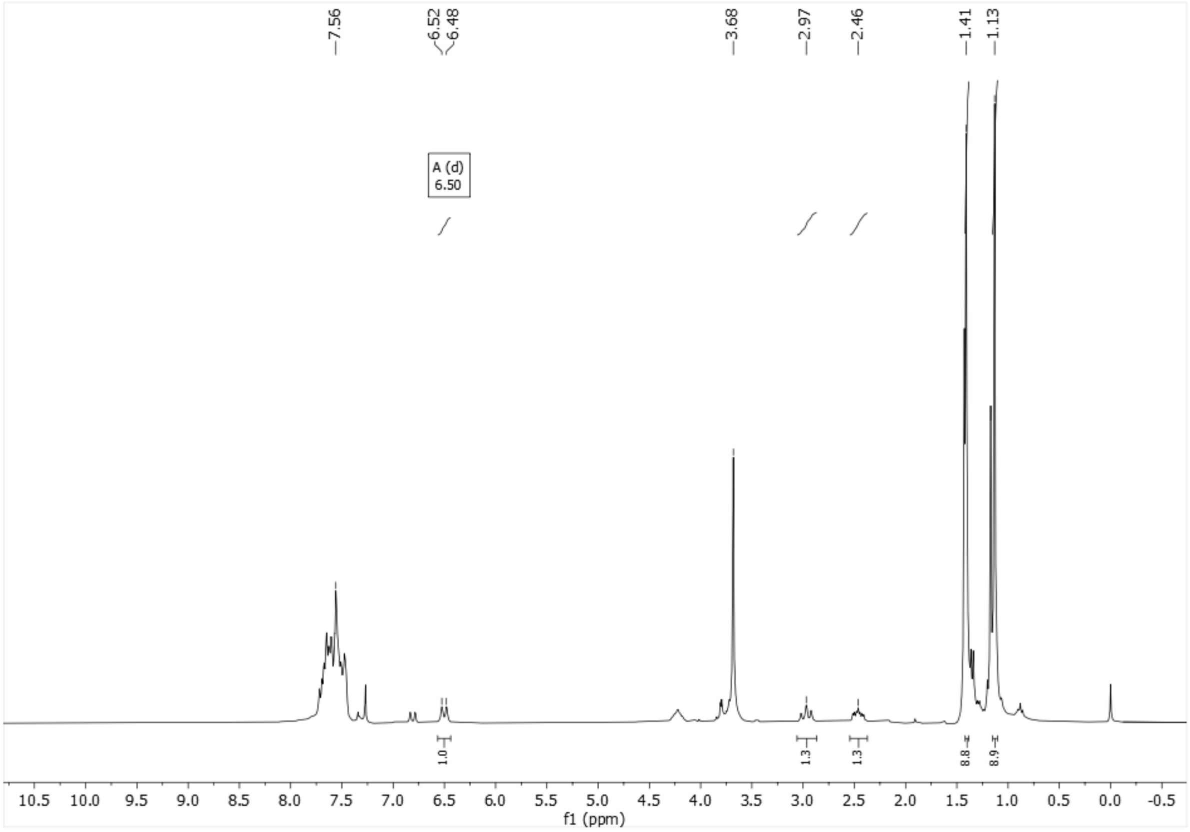

*Figure S48: ^{1}H NMR spectrum of the attempted zwitterion formation from **1** and methyl crotonate recorded from a solution in $CDCl_3$ at 298 K. Marked peaks indicate the presence of a zwitterionic species.*

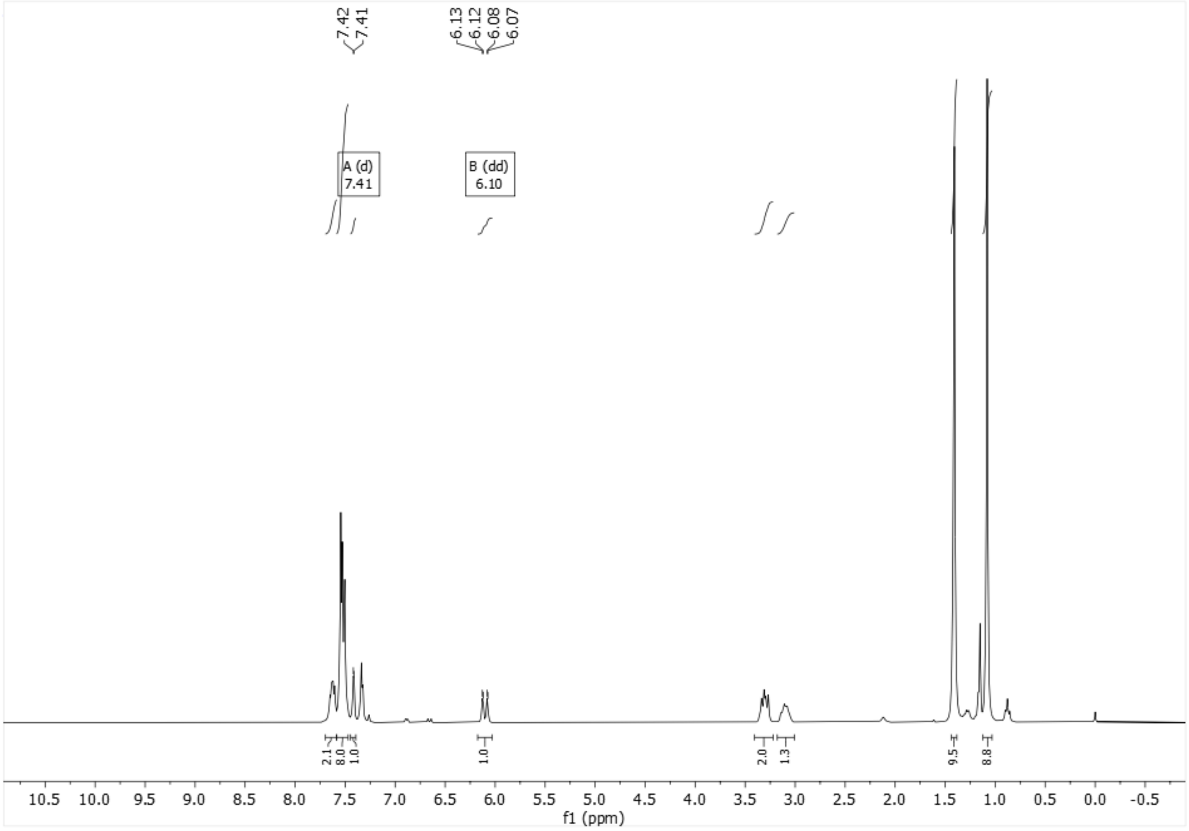

*Figure S49: ^{1}H NMR spectrum of the deuteration experiment for the synthesis of **2a**.*

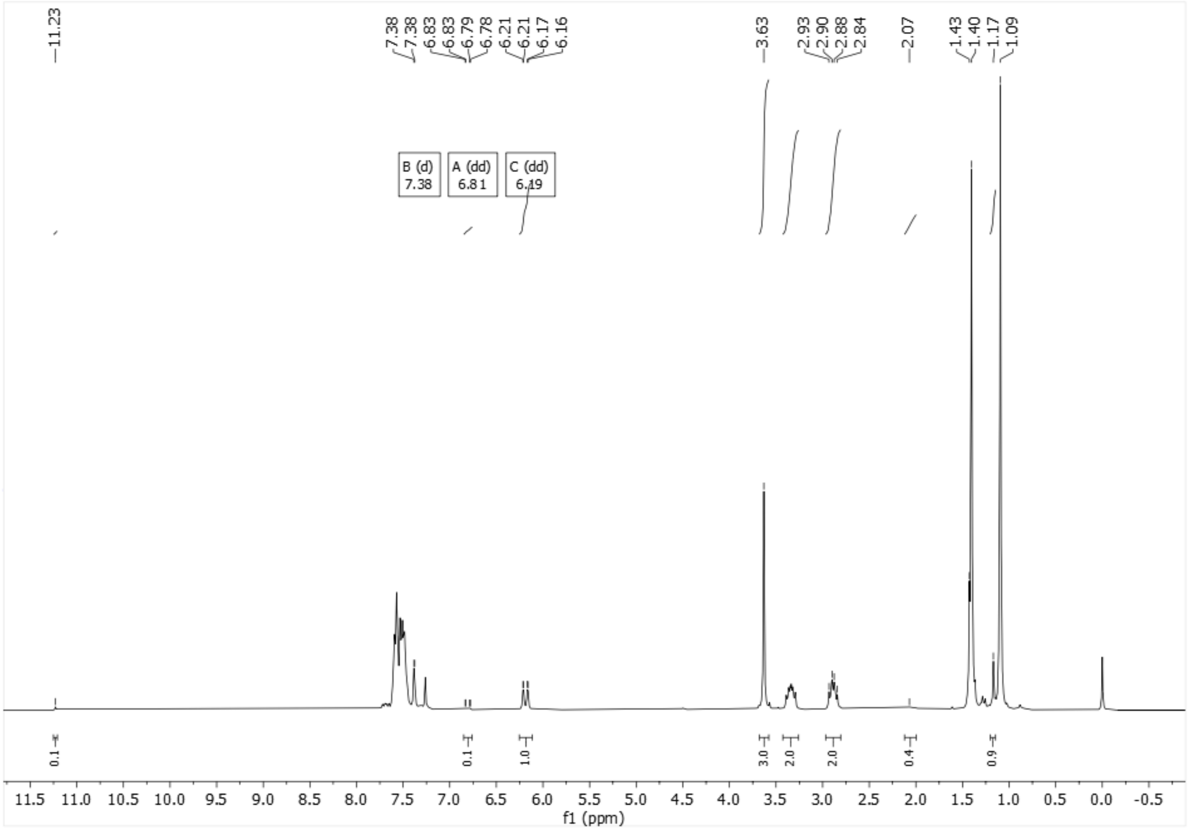

*Figure 50: ^{1}H NMR spectrum of **2d** recorded from a solution in $CDCl_3$ at 298 K after storage as a solid for two months.*

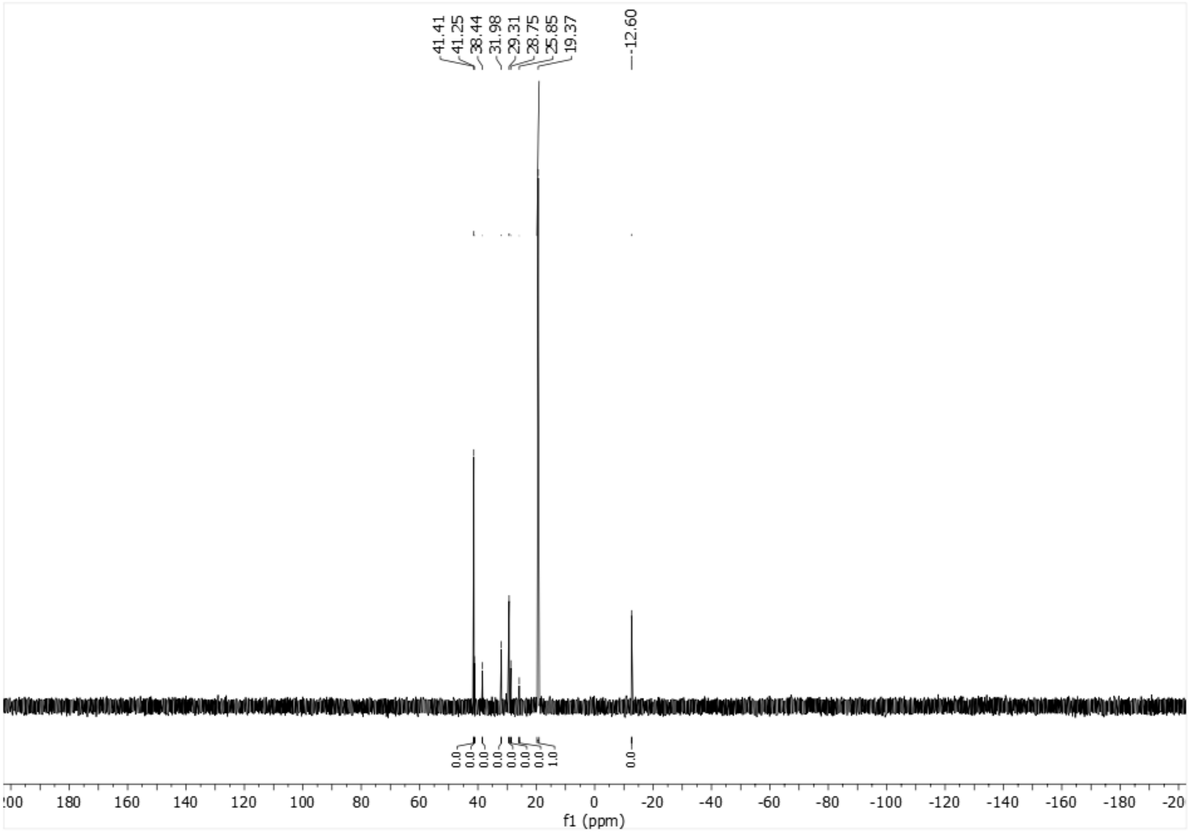

*Figure S51: $^{31}P\{^{1}H\}$ NMR spectrum of **2d** recorded from a solution in $CDCl_3$ at 298 K after storage as a solid for two months.*

For **stability tests**, 20 mg of **2d** (0.042 mmol) were dissolved in 600 µL of the respective solvent ($CDCl_3$ or DMSO-d_6) in an NMR tube. The tubes were sealed with caps and parafilm. The samples were either kept at room temperature or heated to 60 °C. All samples were subjected to ^{1}H and ^{31}P NMR spectroscopy after 24, 48, and 72 h.

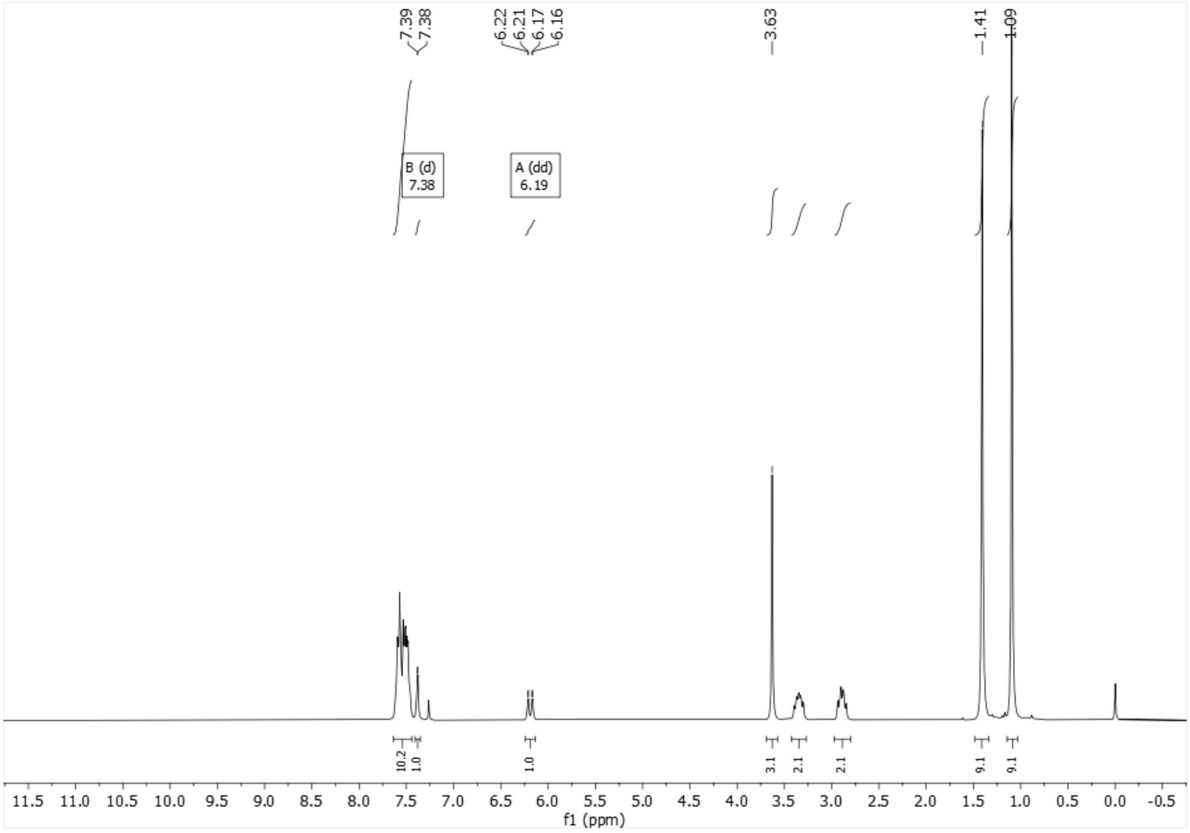

*Figure S52: ^{1}H NMR spectrum of **2d** recorded from a solution in $CDCl_3$ at 298 K after keeping in solution at RT for 24 h.*

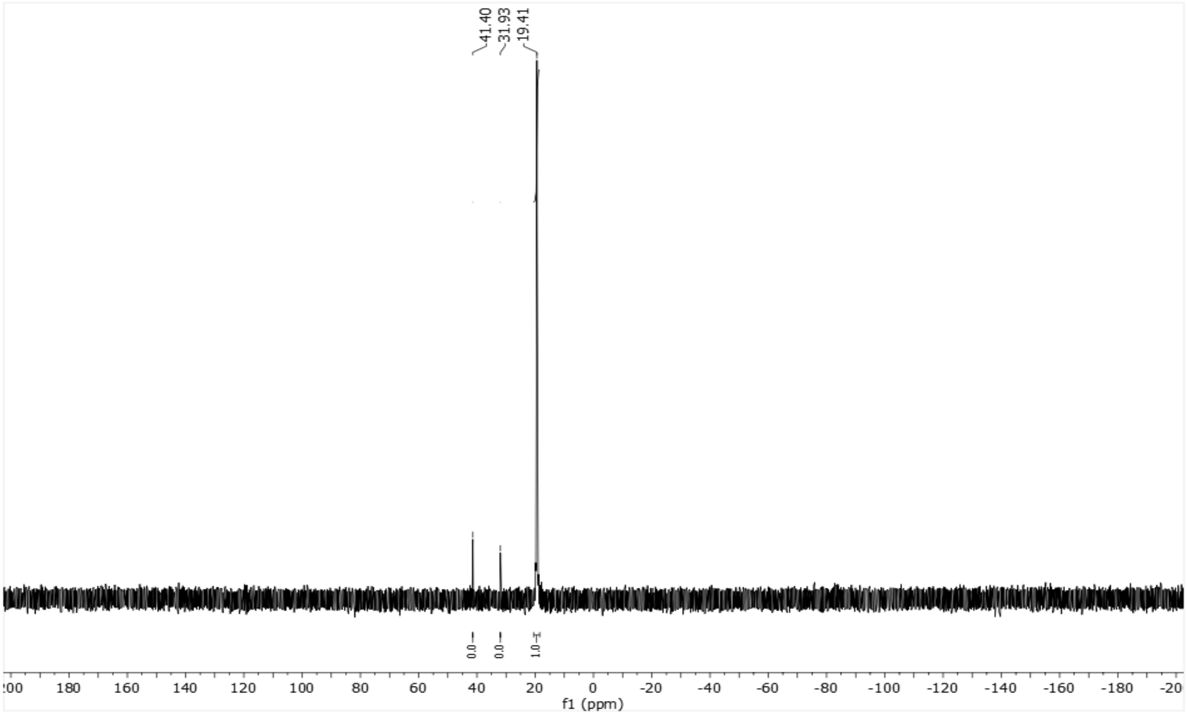

*Figure S53: ^{31}P{^{1}H} NMR spectrum of **2d** recorded from a solution in $CDCl_3$ at 298 K after keeping in solution at RT for 24 h.*

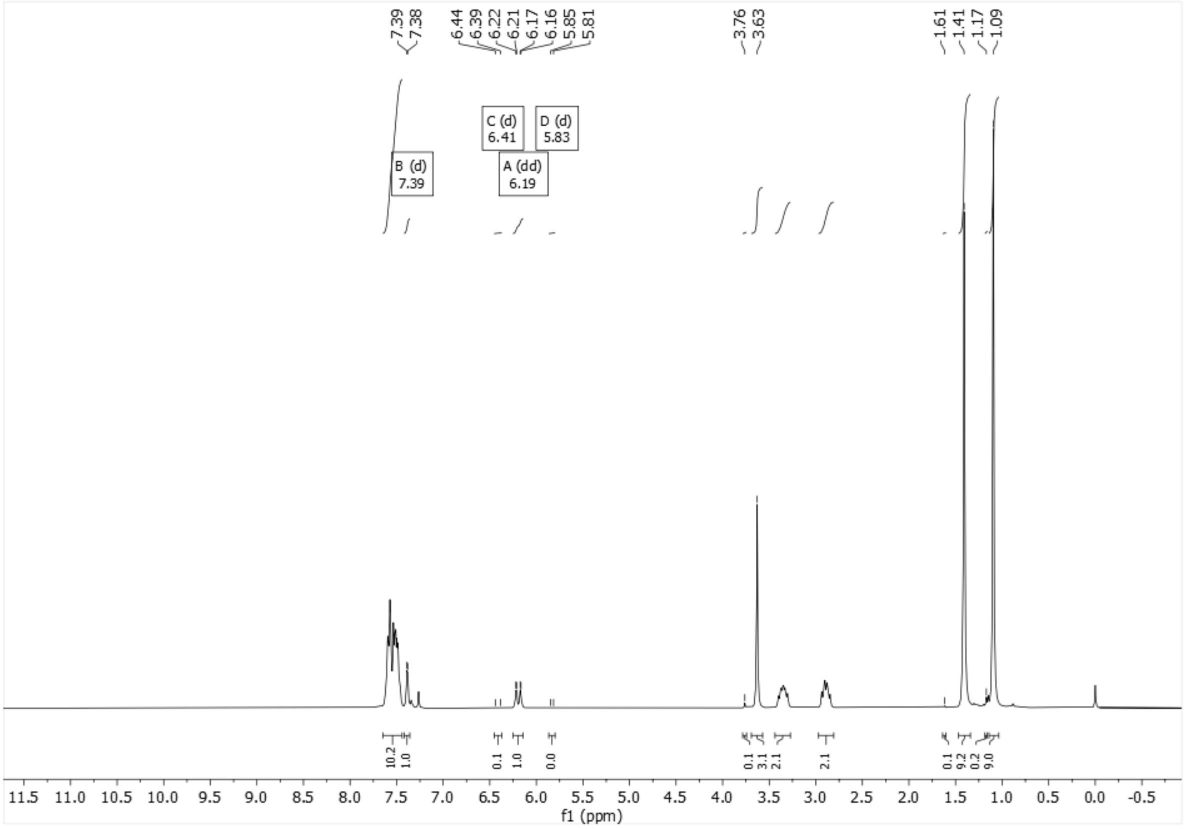

*Figure S54: ${}^{1}H$ NMR spectrum of **2d** recorded from a solution in $CDCl_3$ at 298 K after keeping in solution at 60 °C for 24 h.*

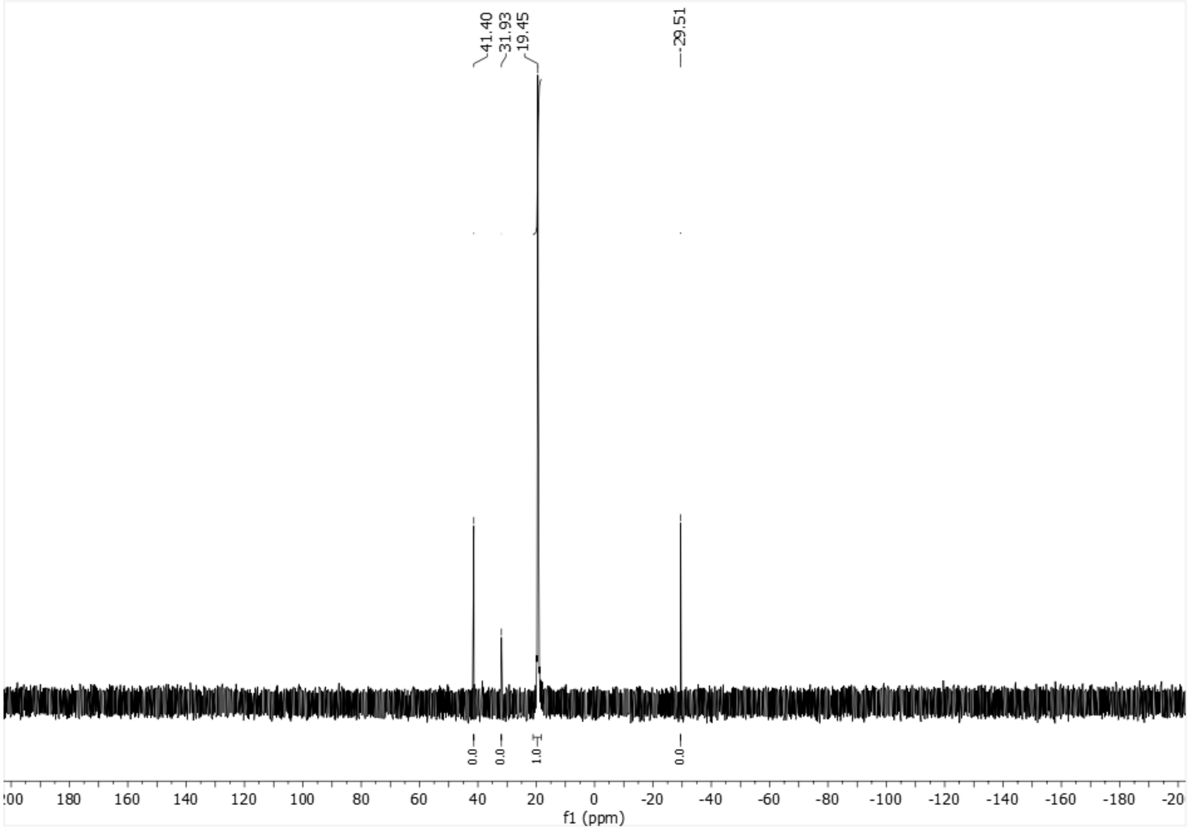

*Figure S55: ${}^{31}P\{{}^{1}H\}$ NMR spectrum of **2d** recorded from a solution in $CDCl_3$ at 298 K after keeping in solution at 60 °C for 24 h.*

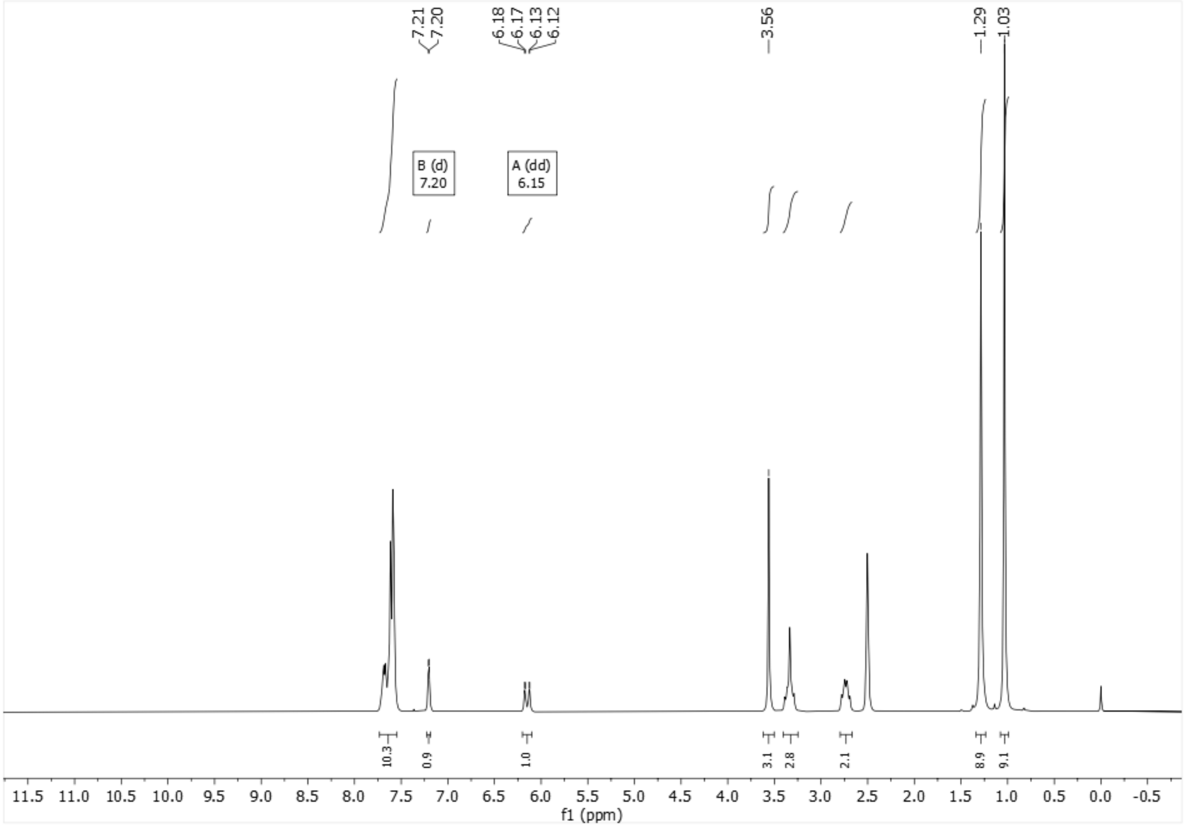

*Figure S56: ^{1}H NMR spectrum of **2d** recorded from a solution in DMSO-d$_6$ at 298 K after keeping in solution at RT for 24 h.*

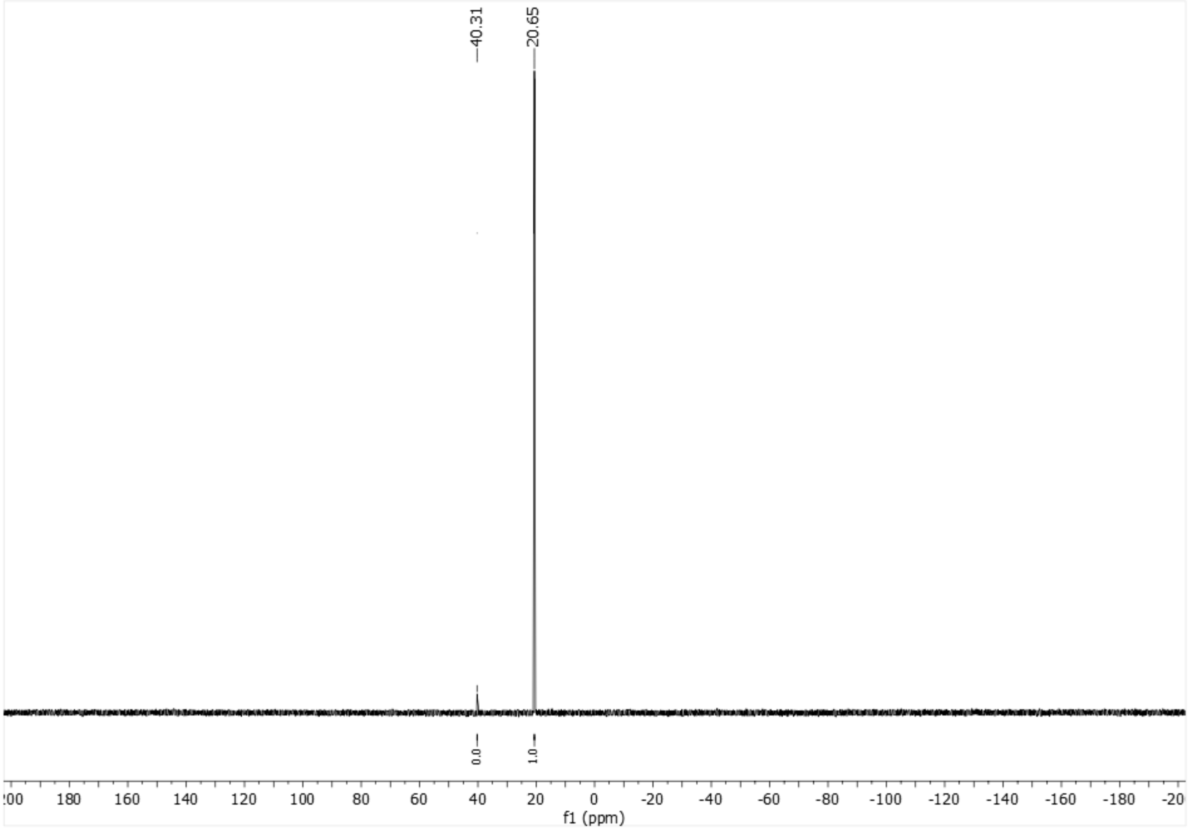

*Figure S57: ^{31}P{^{1}H} NMR spectrum of **2d** recorded from a solution in DMSO-d$_6$ at 298 K after keeping in solution at RT for 24 h.*

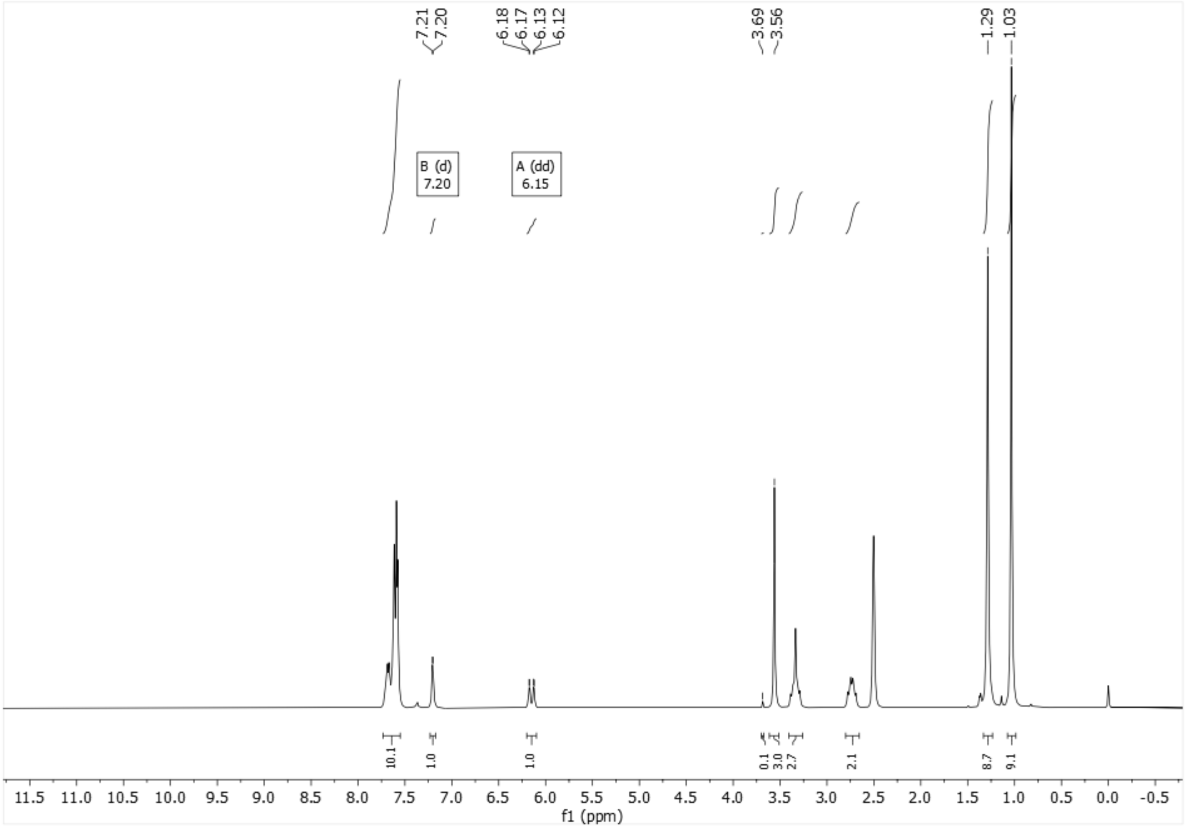

*Figure S58: ^{1}H NMR spectrum of **2d** recorded from a solution in DMSO-d_6 at 298 K after keeping in solution at 60 °C for 24 h.*

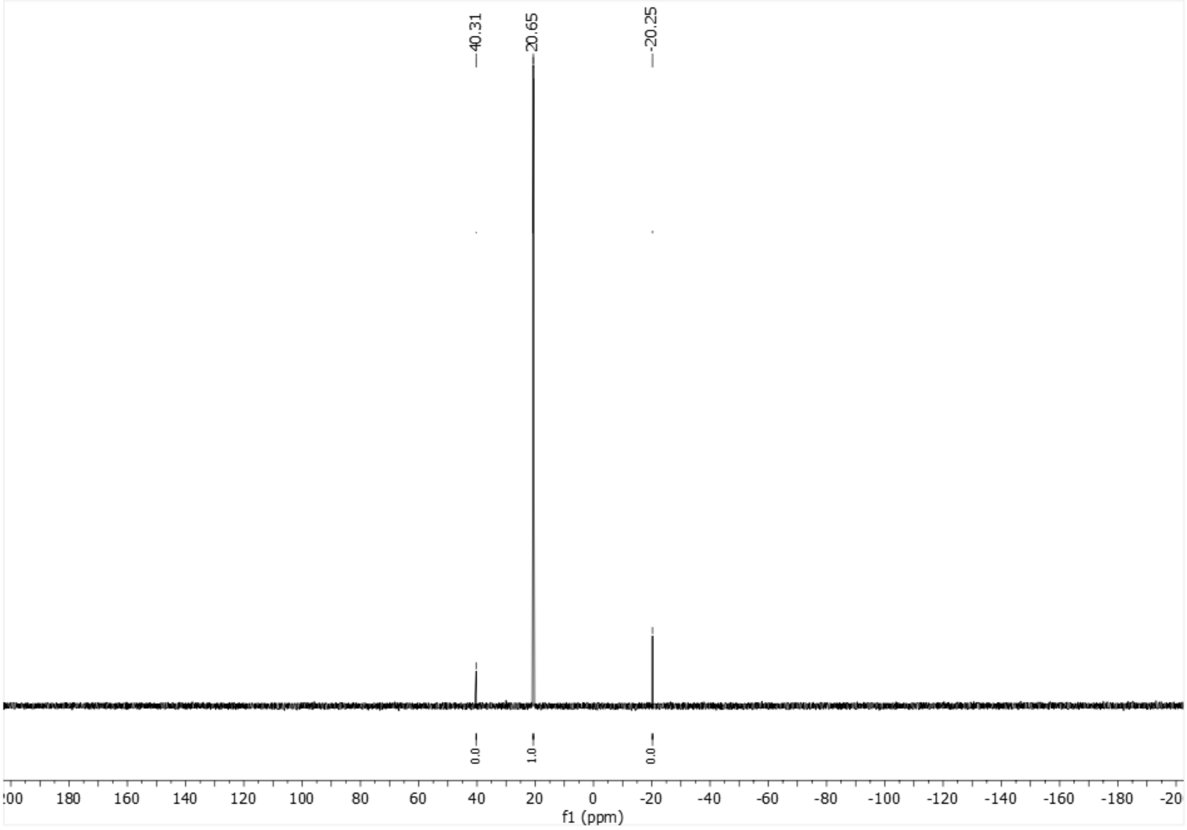

*Figure S59: $^{31}P\{^{1}H\}$ NMR spectrum of **2d** recorded from a solution in DMSO-d_6 at 298 K after keeping in solution at 60 °C for 24 h.*

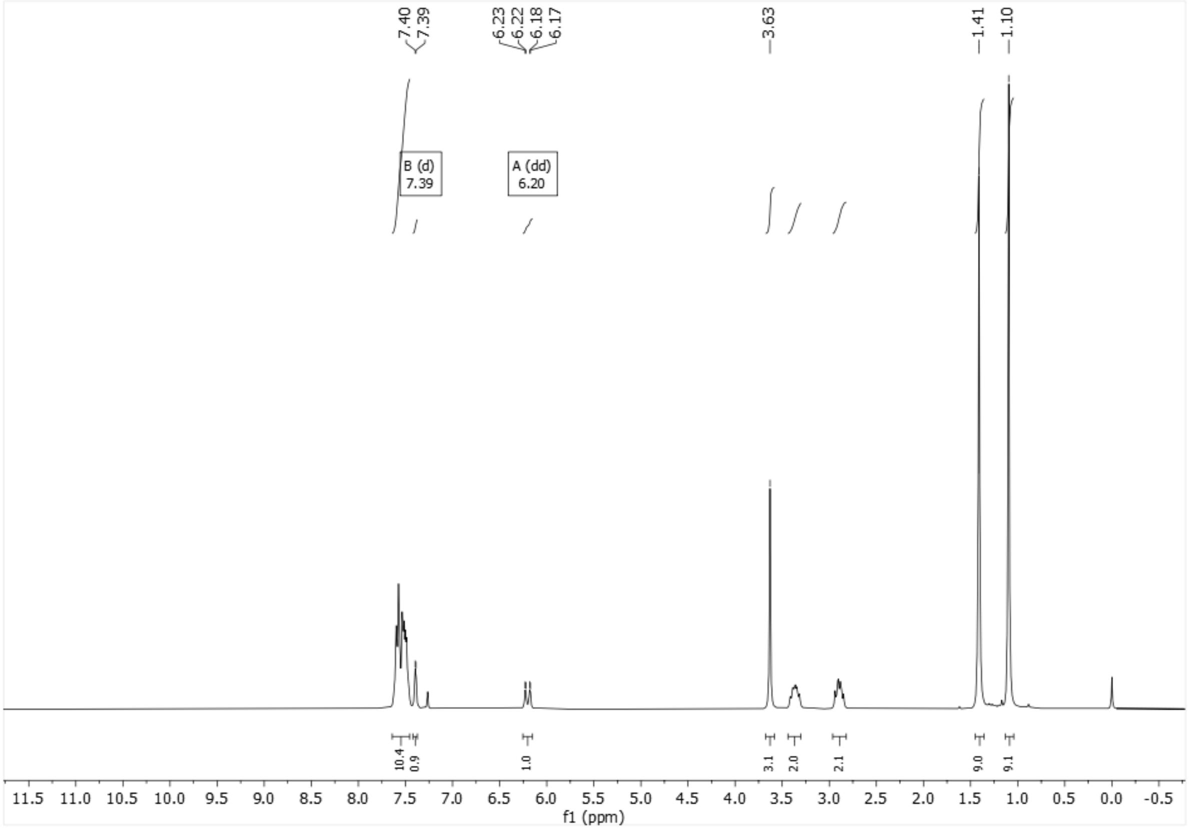

*Figure S60: ^{1}H NMR spectrum of **2d** recorded from a solution in $CDCl_3$ at 298 K after keeping in solution at RT for 48 h.*

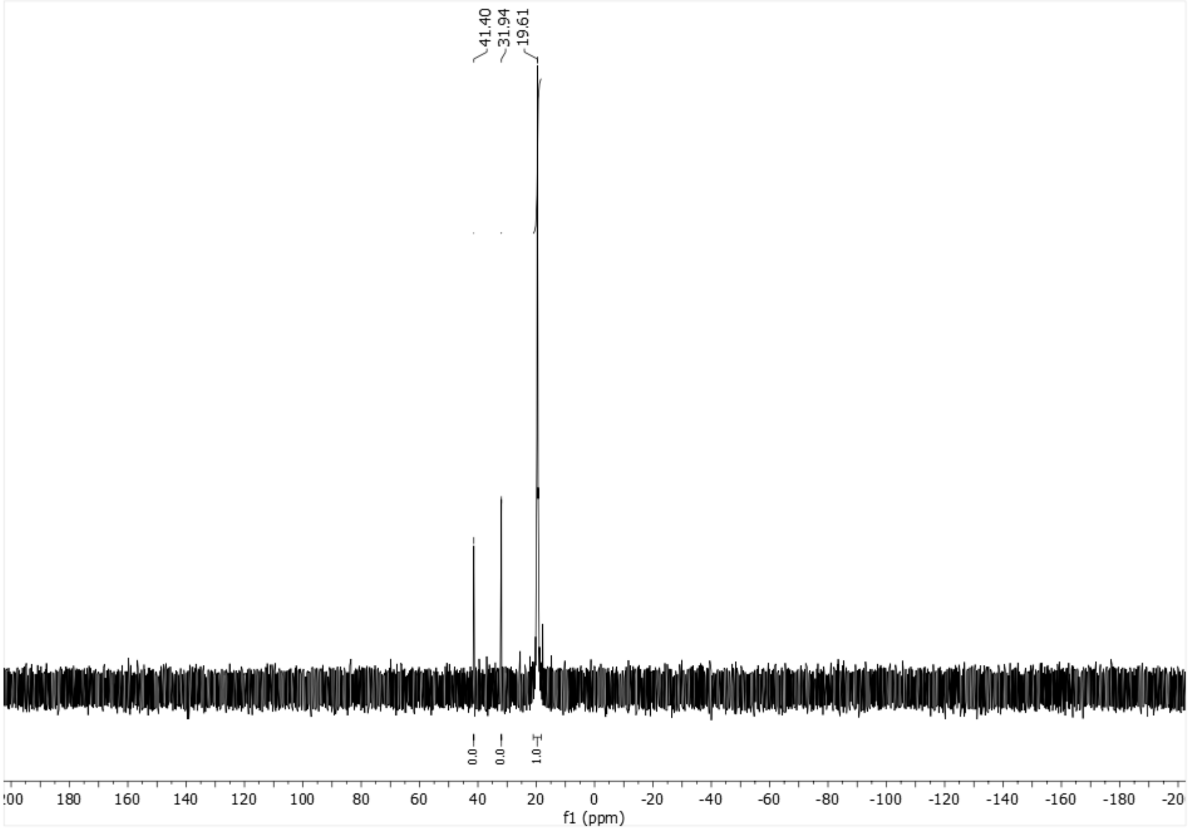

*Figure S61: ^{31}P{^{1}H} NMR spectrum of **2d** recorded from a solution in $CDCl_3$ at 298 K after keeping in solution at RT for 48 h.*

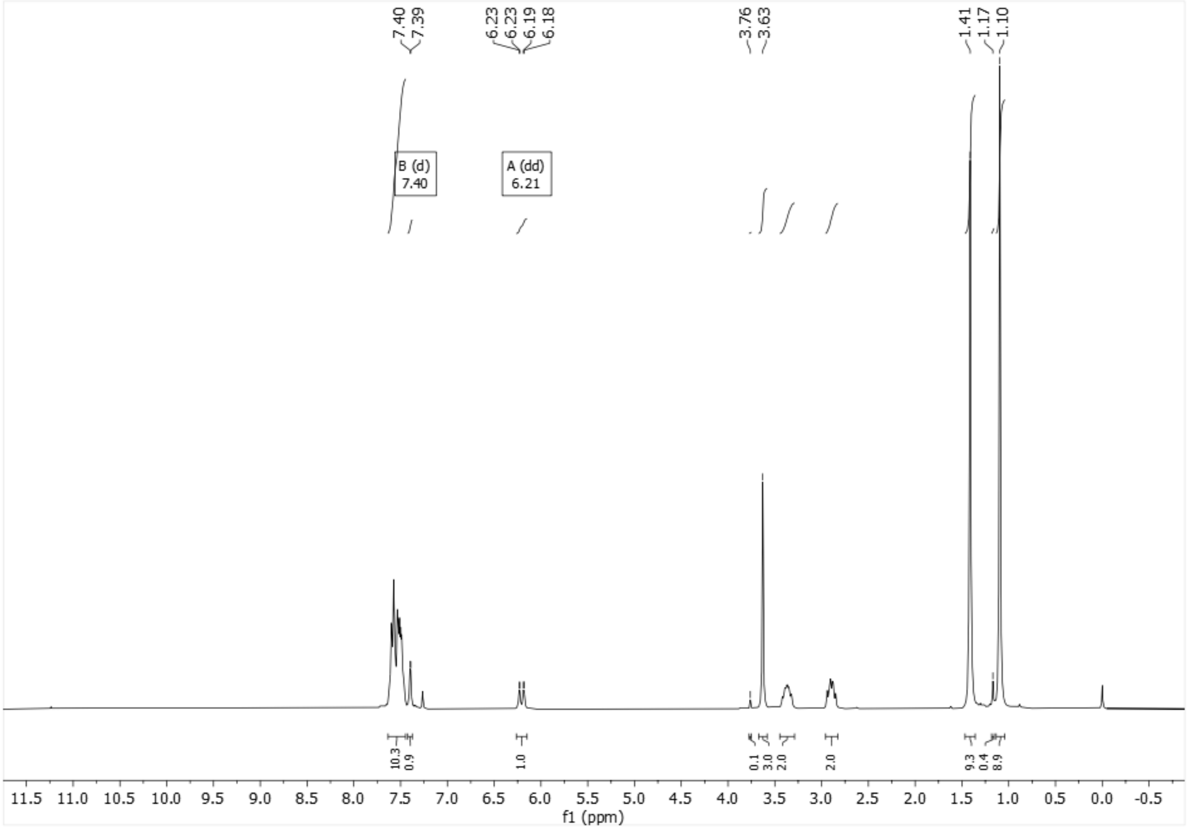

*Figure S62: ¹H NMR spectrum of **2d** recorded from a solution in $CDCl_3$ at 298 K after keeping in solution at 60 °C for 48 h.*

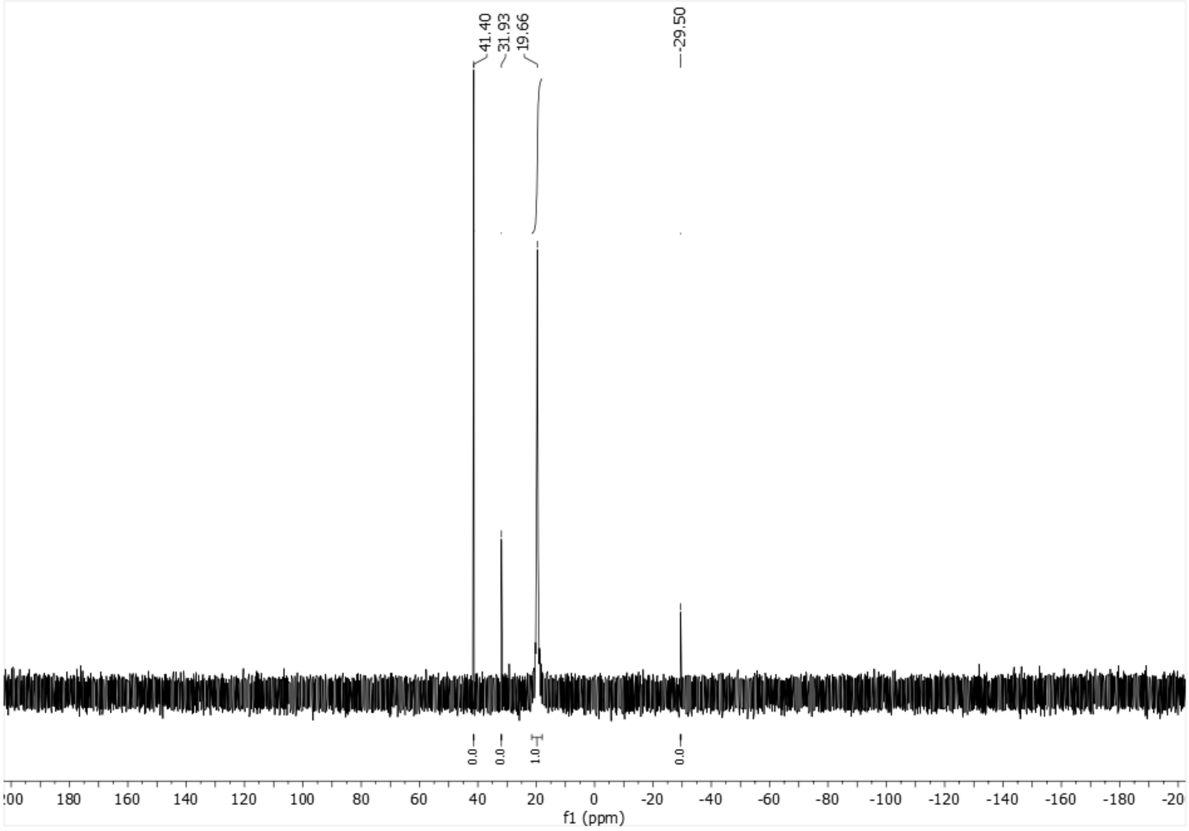

*Figure S63: $^{31}P\{^{1}H\}$ NMR spectrum of **2d** recorded from a solution in $CDCl_3$ at 298 K after keeping in solution at 60 °C for 48 h.*

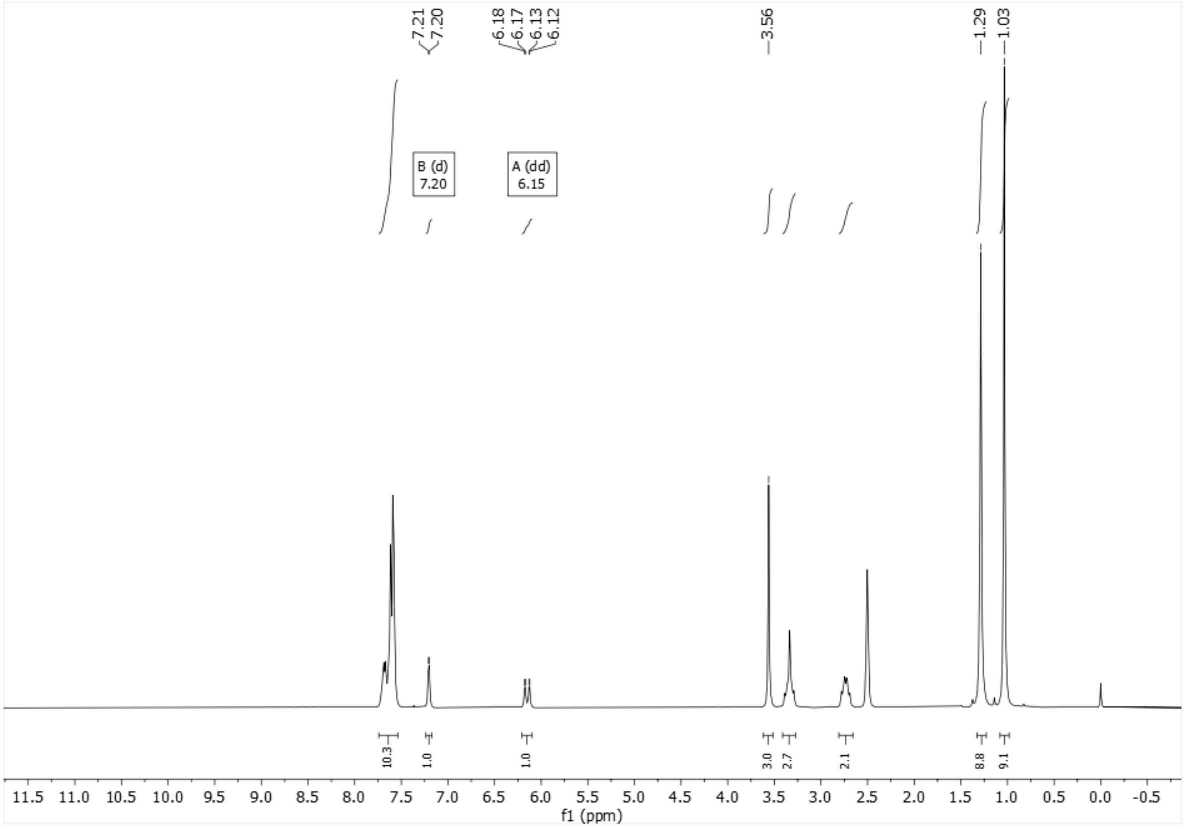

*Figure S64: ^{1}H NMR spectrum of **2d** recorded from a solution in DMSO-d_6 at 298 K after keeping in solution at RT for 48 h.*

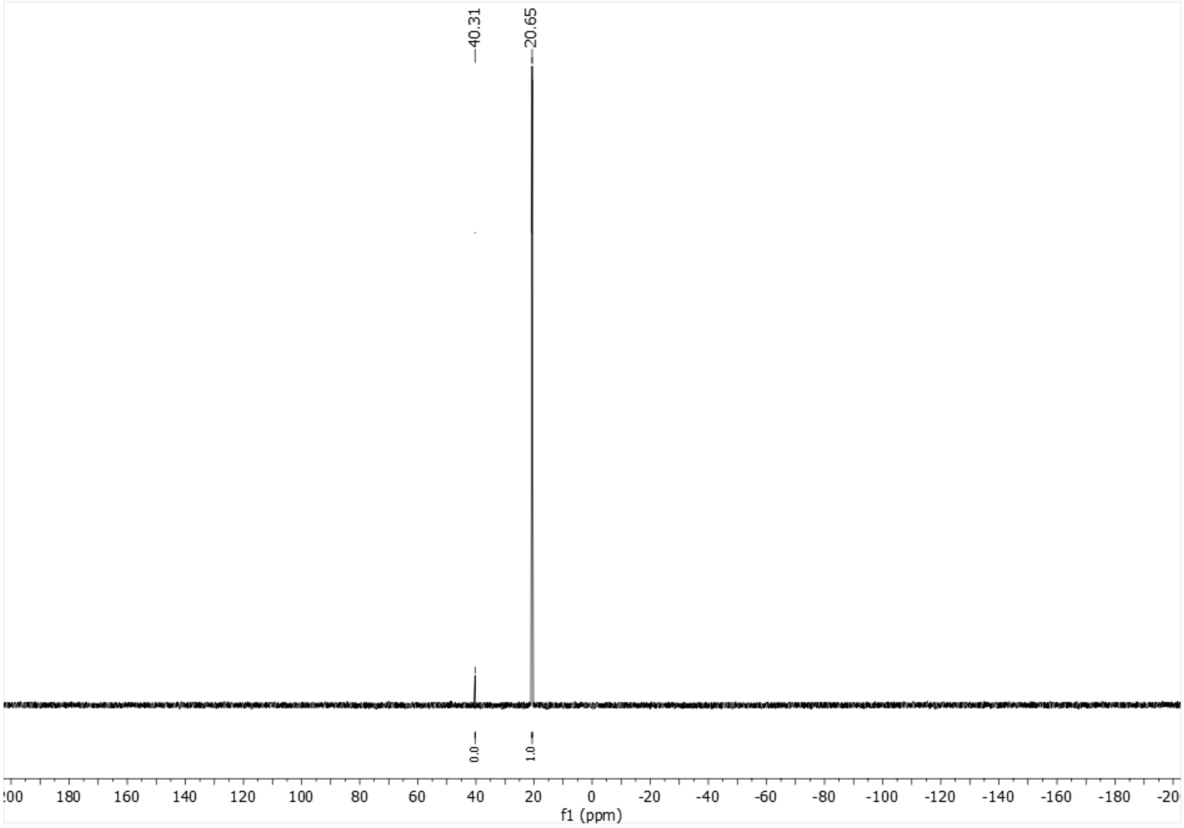

*Figure S65: $^{31}P\{^{1}H\}$ NMR spectrum of **2d** recorded from a solution in DMSO-d_6 at 298 K after keeping in solution at RT for 48 h.*

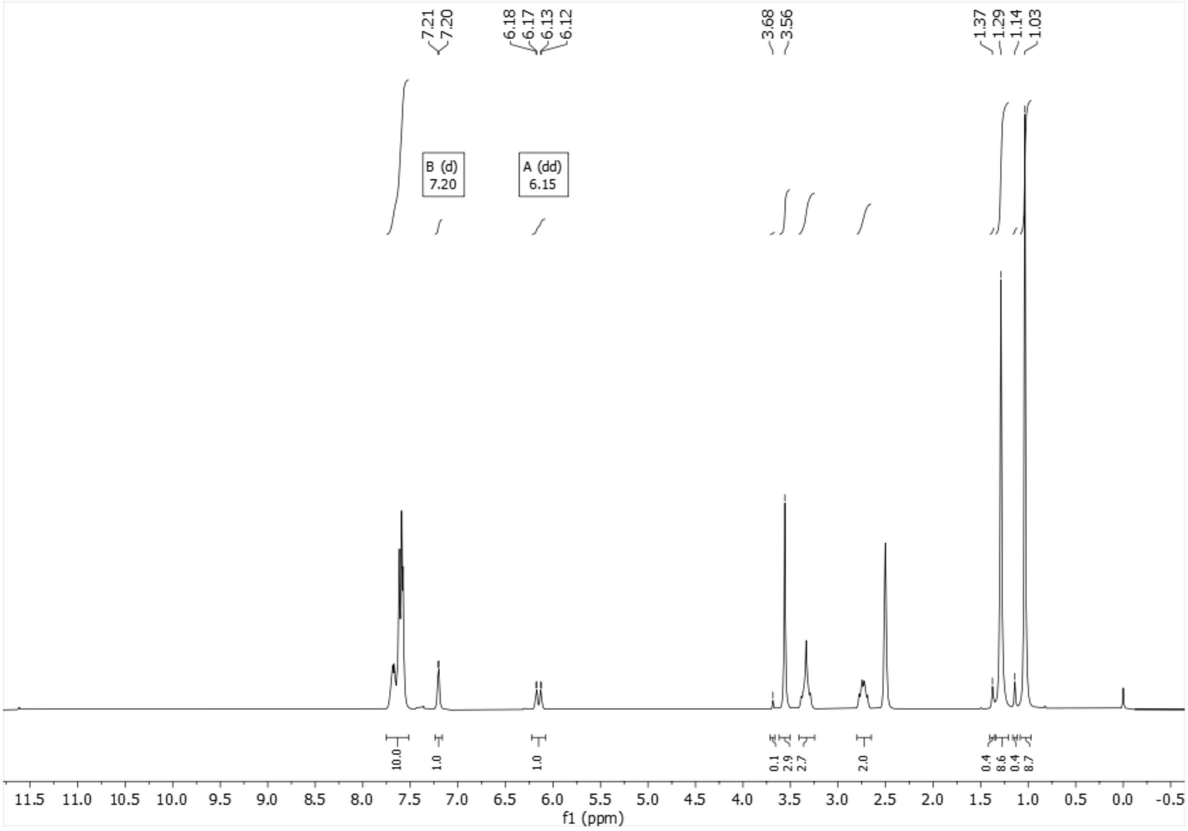

*Figure S66: ^{1}H NMR spectrum of **2d** recorded from a solution in DMSO-d_6 at 298 K after keeping in solution at 60 °C for 48 h.*

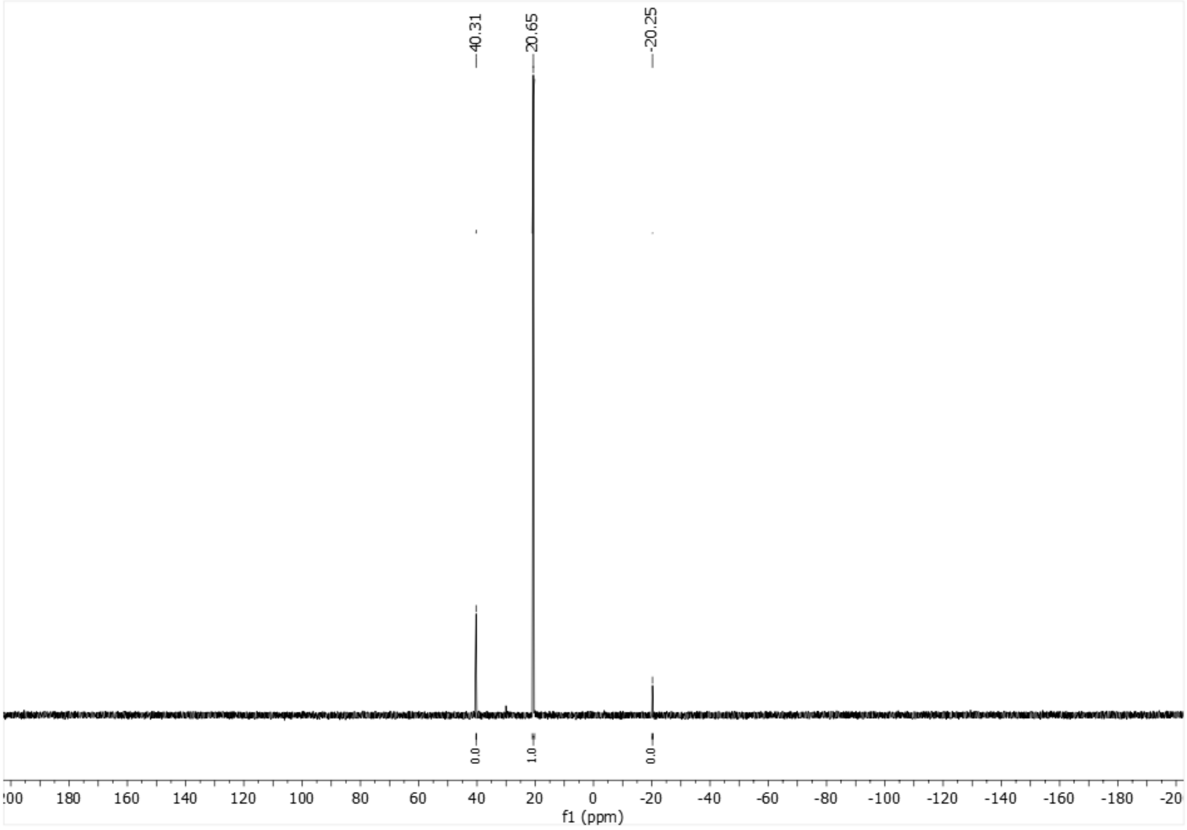

*Figure S67: $^{31}P\{^{1}H\}$ NMR spectrum of **2d** recorded from a solution in DMSO-d_6 at 298 K after keeping in solution at 60 °C for 48 h.*

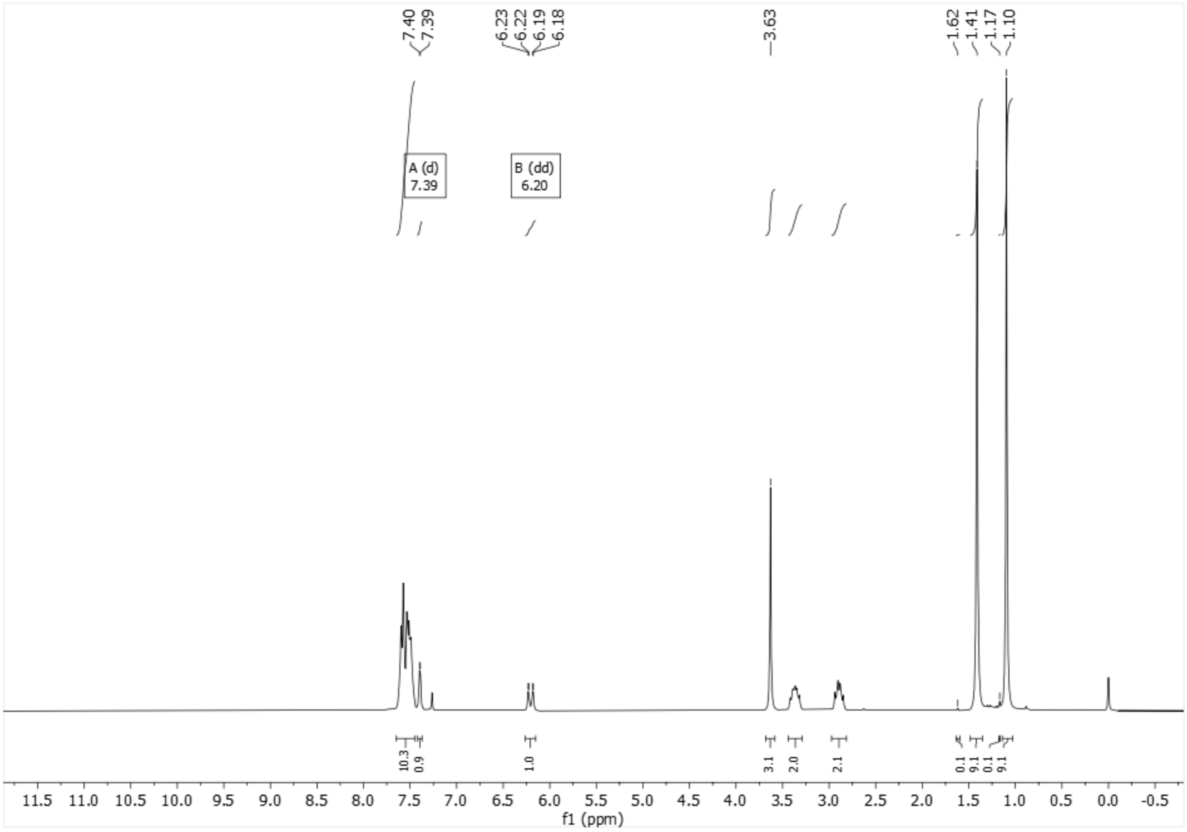

*Figure S68: ^{1}H NMR spectrum of **2d** recorded from a solution in $CDCl_3$ at 298 K after keeping in solution at RT for 72 h.*

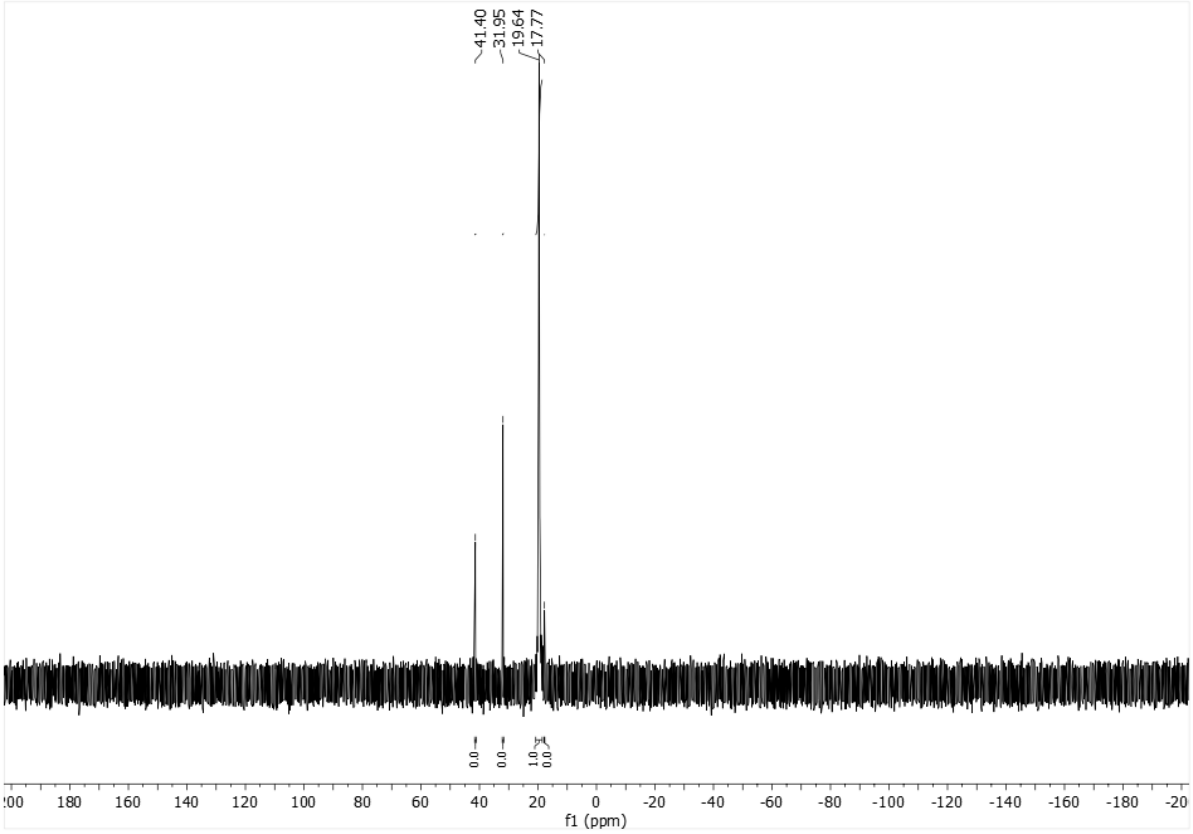

*Figure S69: $^{31}P\{^{1}H\}$ NMR spectrum of **2d** recorded from a solution in $CDCl_3$ at 298 K after keeping in solution at RT for 72 h.*

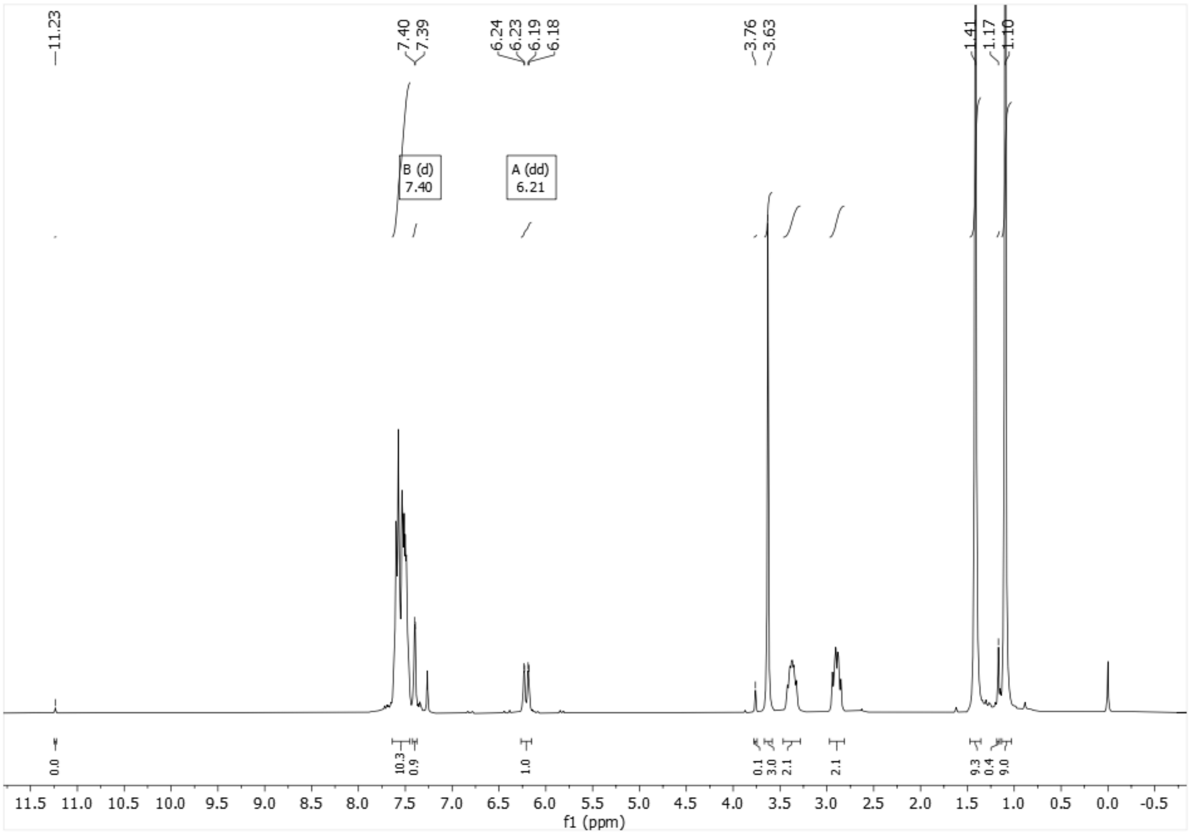

*Figure S70: ¹H NMR spectrum of **2d** recorded from a solution in $CDCl_3$ at 298 K after keeping in solution at 60 °C for 72 h.*

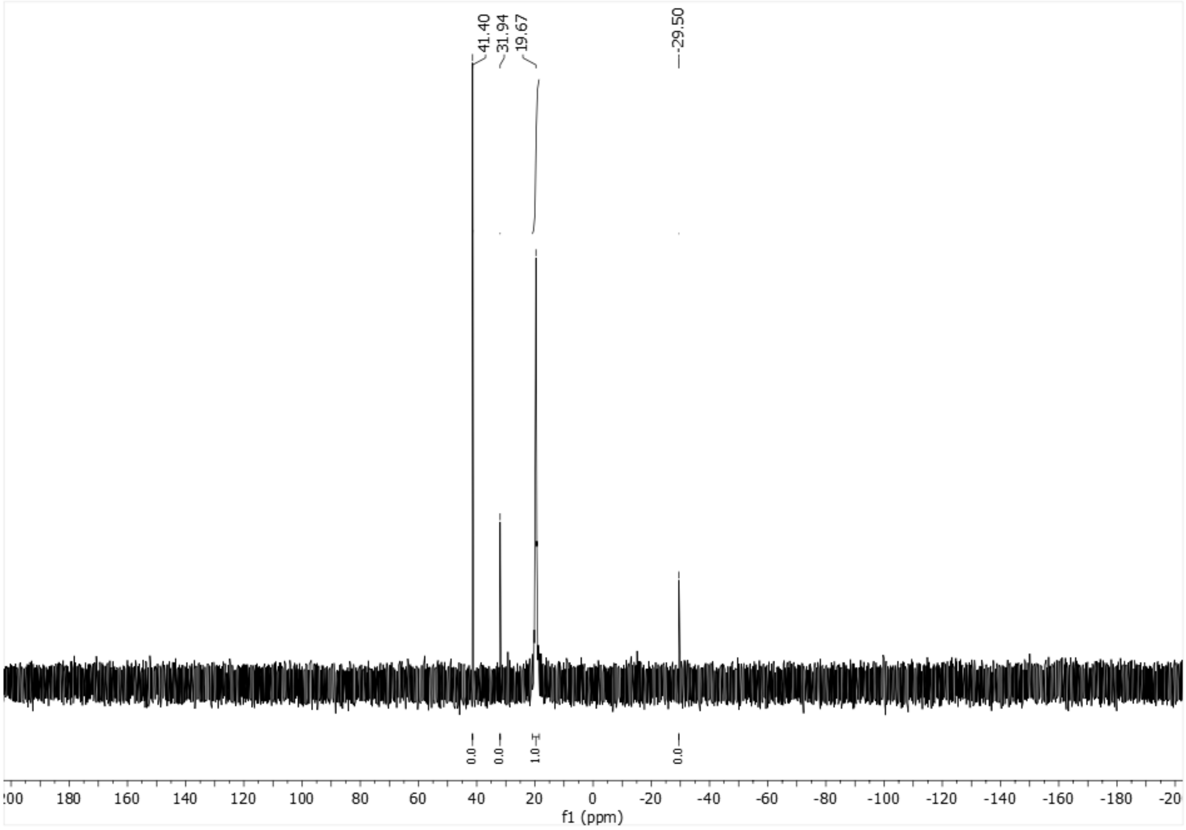

*Figure S71: ³¹P{¹H} NMR spectrum of **2d** recorded from a solution in $CDCl_3$ at 298 K after keeping in solution at 60 °C for 72 h.*

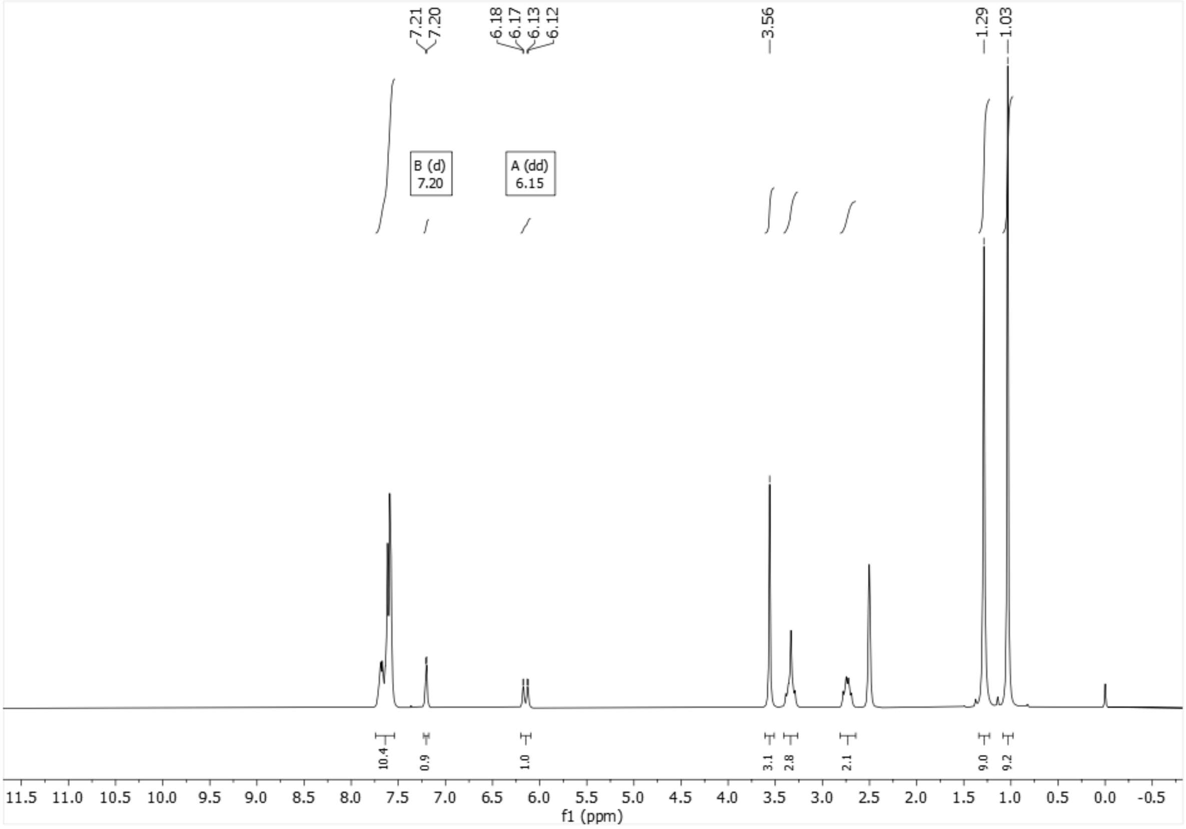

*Figure S72: ^{1}H NMR spectrum of **2d** recorded from a solution in DMSO-d_6 at 298 K after keeping in solution at RT for 72 h.*

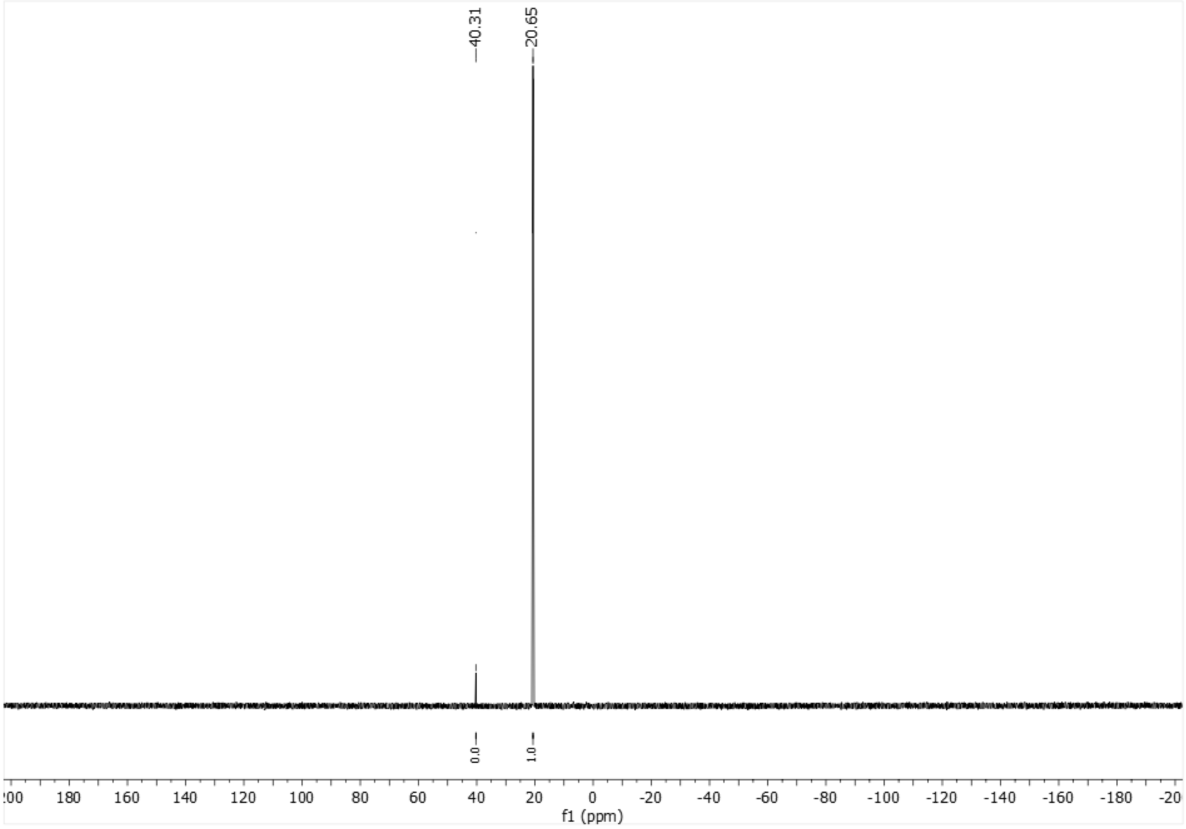

*Figure S73: $^{31}P\{^{1}H\}$ NMR spectrum of **2d** recorded from a solution in DMSO-d_6 at 298 K after keeping in solution at RT for 72 h.*

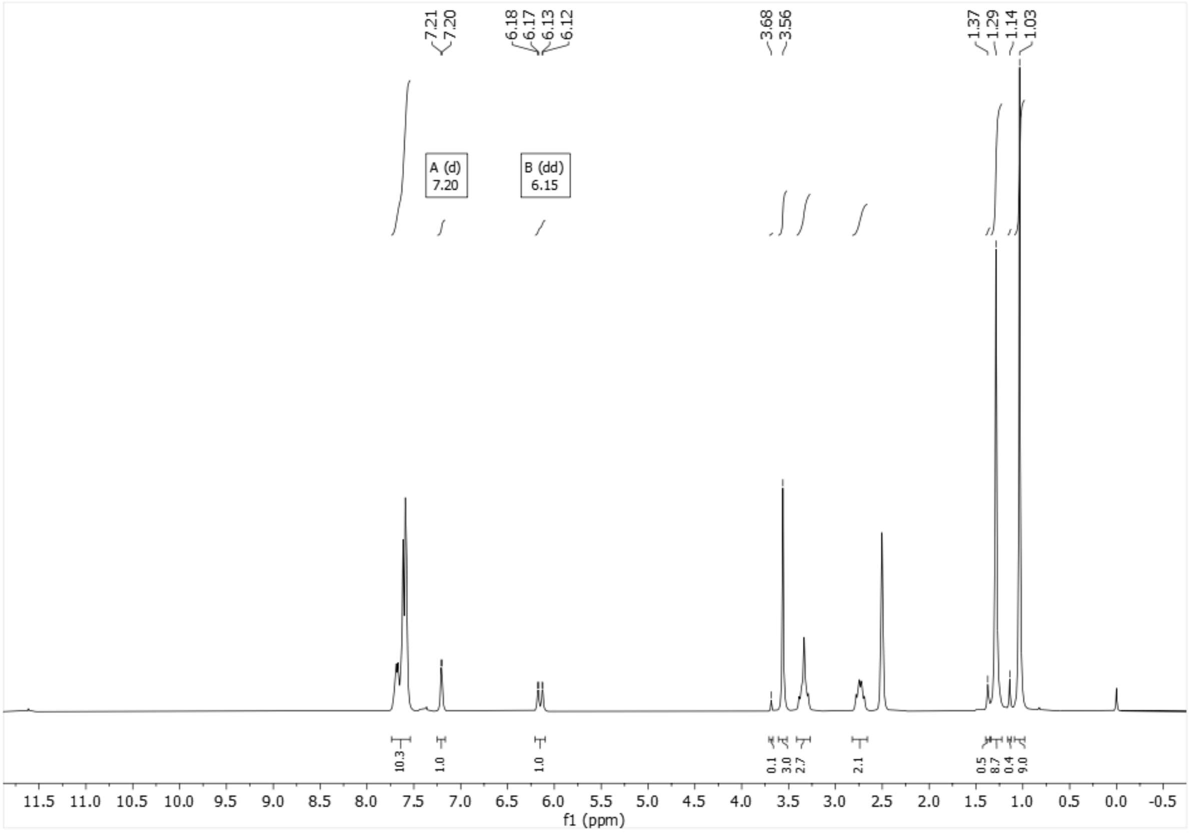

*Figure S74: ^{1}H NMR spectrum of **2d** recorded from a solution in DMSO-d_6 at 298 K after keeping in solution at 60 °C for 72 h.*

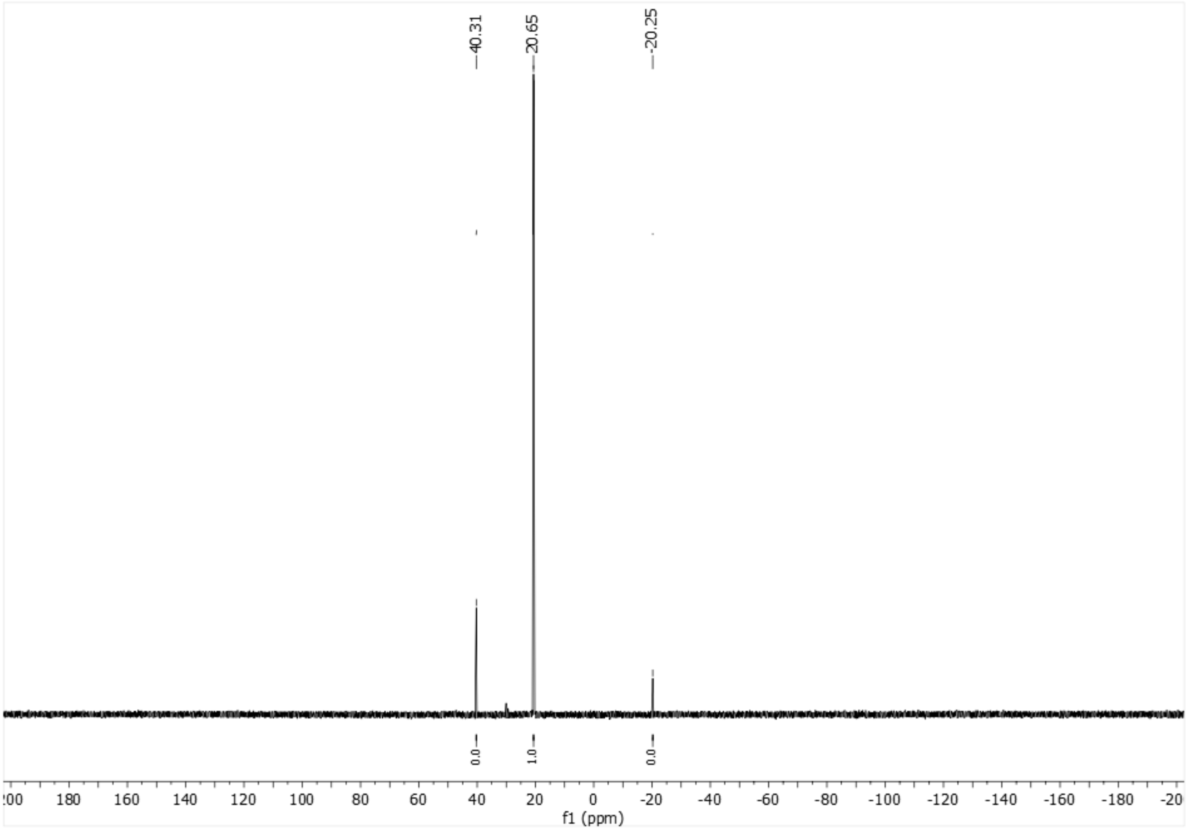

*Figure S75: $^{31}P\{^{1}H\}$ NMR spectrum of **2d** recorded from a solution in DMSO-d_6 at 298 K after keeping in solution at 60 °C for 72 h.*

Kinetic investigations

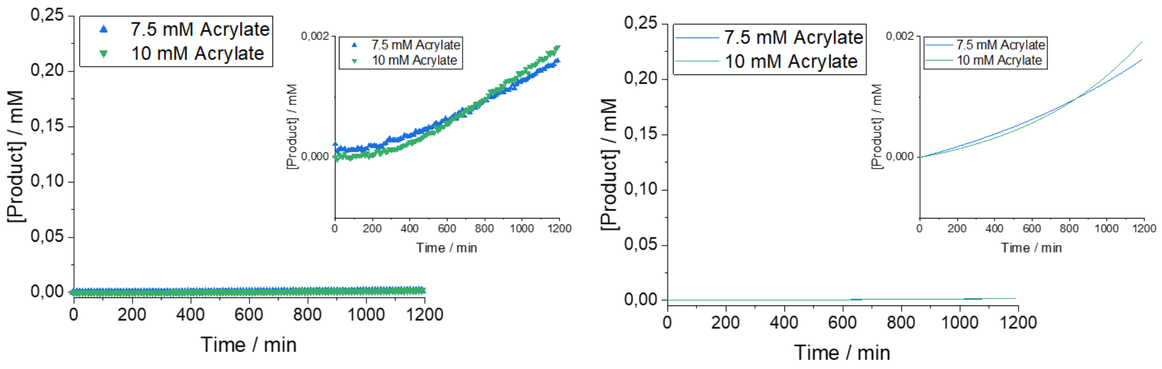

*Figure S76: Experimental (left) and simulated (right) time conversion plots for the reaction of **1** and acrylonitrile in chloroform*

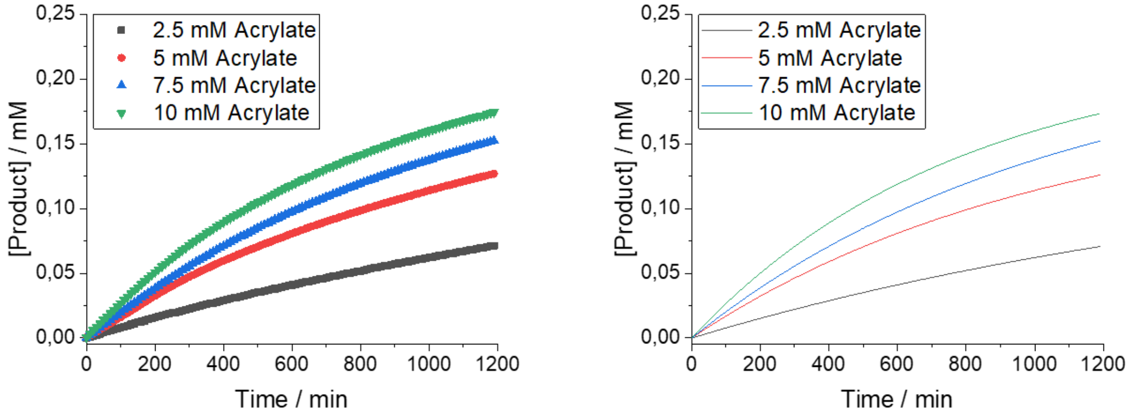

*Figure S77: Experimental (left) and simulated (right) time conversion plots for the reaction of **1** and acrylonitrile in methanol*

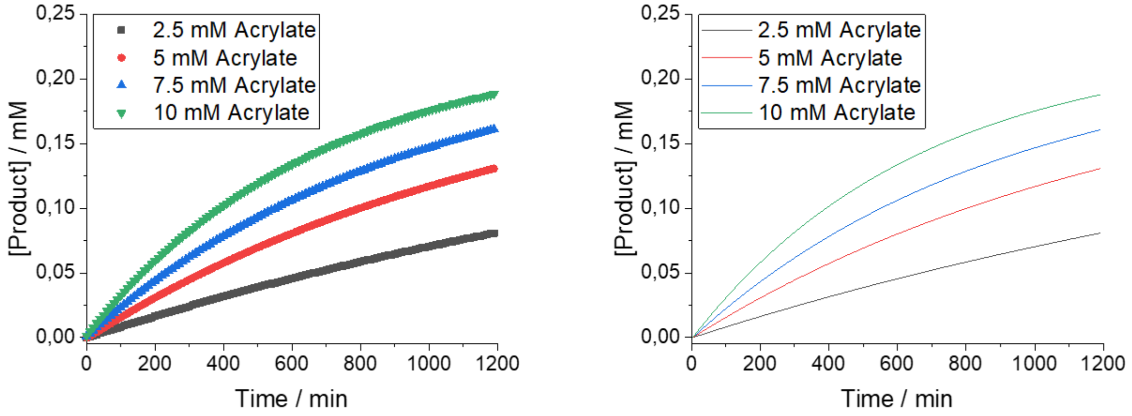

*Figure S78: Experimental (left) and simulated (right) time conversion plots for the reaction of **1** and acrylamide in chloroform*

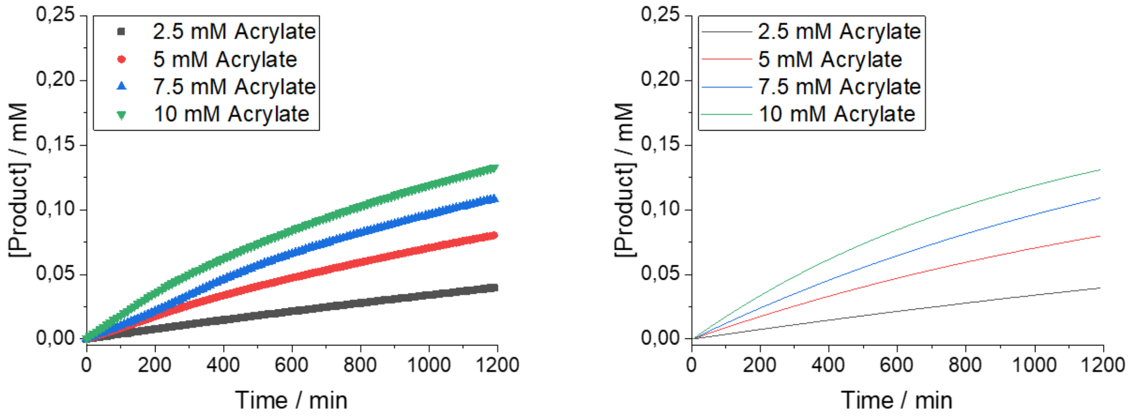

*Figure S79: Experimental (left) and simulated (right) time conversion plots for the reaction of **1** and acrylamide in methanol*

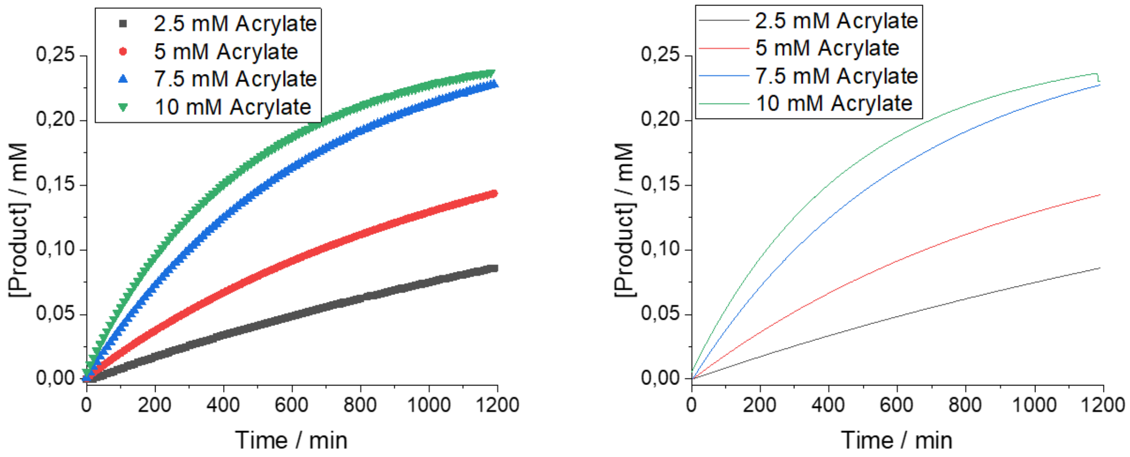

*Figure S80: Experimental (left) and simulated (right) time conversion plots for the reaction of **1** and methyl acrylate in chloroform*

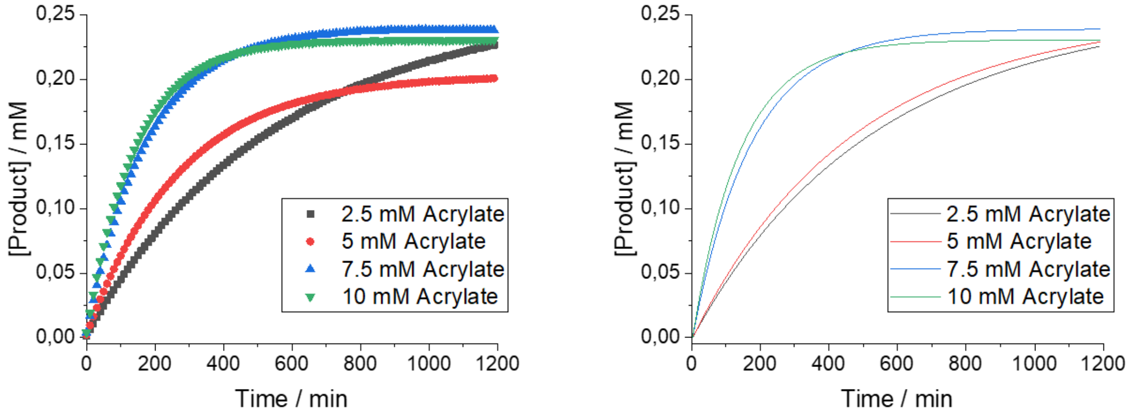

*Figure S81: Experimental (left) and simulated (right) time conversion plots for the reaction of **1** and methyl acrylate in methanol*

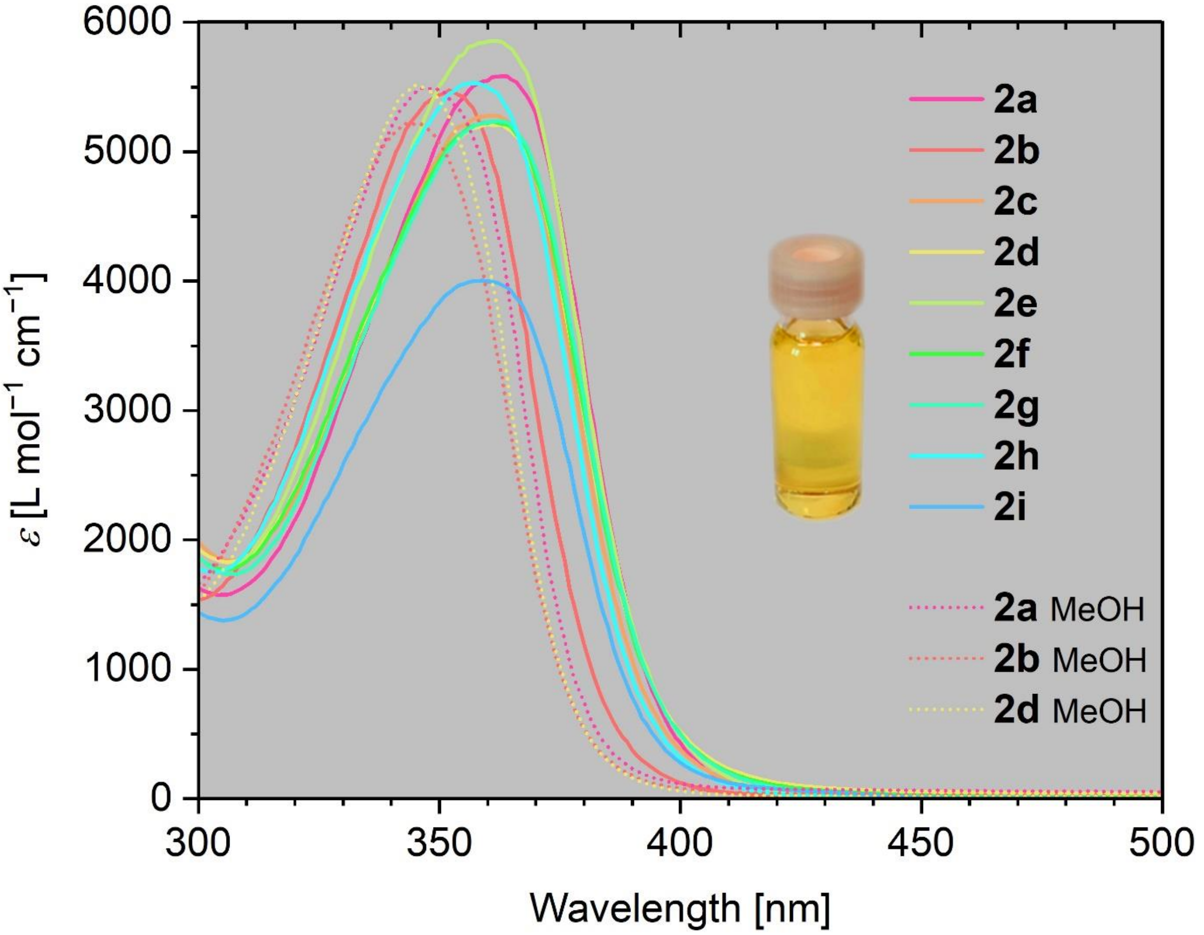

*Figure S82: UV–vis spectra of **2a**–**i** in chloroform (straight lines) and **2a**, **2b** and **2d** in methanol (dotted lines); the inset shows a photograph of a vial containing a solution of **2a** in chloroform.*
